# Measures of decision-making ability and functional outcomes in older adults: results from a scoping review in the ARMCADA study

**DOI:** 10.3389/fpsyg.2025.1540493

**Published:** 2025-03-21

**Authors:** Tatiana Karpouzian-Rogers, Elizabeth M. Dworak, Patricia Bucko, Emily H. Ho, Berivan Ece, Molly A. Mather, Miriam A. Novack, Sarah Pila, Zahra Hosseinian, LaToya Hall, Tarry Mkhize, Adrianna M. Bassard, Richard C. Gershon, Sandra Weintraub

**Affiliations:** ^1^Department of Psychiatry and Behavioral Sciences, Northwestern University Feinberg School of Medicine, Chicago, IL, United States; ^2^Department of Medical Social Sciences, Northwestern University Feinberg School of Medicine, Chicago, IL, United States; ^3^Mesulam Center for Cognitive Neurology and Alzheimer's Disease, Northwestern University Feinberg School of Medicine, Chicago, IL, United States; ^4^Department of Gerontology, Wayne State University, Detroit, MI, United States

**Keywords:** activities of daily living, daily functioning, decision-making, aging, cognitive impairment, functional outcomes

## Abstract

**Background:**

Though often not routinely assessed in older adults, declines in decision-making (DM) abilities are often observed in aging and may lead to adverse outcomes in multiple aspects of daily functioning. The Advancing Reliable Measurement in Cognitive Aging and Decision-making Ability (ARMCADA) research initiative seeks to address these issues. This scoping review investigates the current published literature on existing DM measures in aging samples, with emphasis on the domain of functional outcomes, defined as skills or behaviors related to one's ability to live independently.

**Methods:**

We identified studies published between 2018–2023 using key words related to DM abilities and functional outcomes in aging populations through multiple databases. Titles and abstracts were first reviewed by two reviewers, full texts were then screened, and data were extracted from included articles.

**Results:**

The scoping review identified 16,278 articles across domains with adults aged 45 and older. After screening and extraction, 705 total articles were included; 301 were related to functional outcomes and, from these, 231 distinct measures were identified. Mode of administration of most measures were self-administered with supervision, followed by examiner-administered, and most were conducted with clinical samples (e.g., MCI/AD, chronic health conditions, and Parkinson's disease, or clinical samples and a control group).

**Discussion:**

The goal of the current scoping review is to provide a comprehensive examination of the current DM measures in older adults; this article focuses on the domain of functional outcomes. This scoping review guides a project to create and validate measures that can efficiently assess DM abilities in older adults across the cognitive aging spectrum.

## 1 Introduction

Decision-making is a fundamental behavior that involves the synthesis of multimodal sensory inputs, autonomic and emotional responses, past associations, and future goals, which are integrated with information regarding uncertainty, cost-benefit, and risk and then applied to an action (Fellows, 2004). Successful decision-making requires multiple skills, including the ability to understand and integrate information, identify what information may be relevant during the decision process, and inhibit impulsive responses (Finucane and Gullion, 2010). In older adults, decision-making (DM) abilities may decline over time, potentially due to reductions in fluid cognitive abilities (i.e. reasoning and problem-solving), errors in comprehension, and increased inconsistency in decision-making (Finucane et al., 2002). These changes can be observed across medical, neurologic, or psychiatric conditions (Boettger et al., 2015), though are particularly pronounced in older adults with cognitive impairment, including individuals with clinical diagnoses of Mild Cognitive Impairment (MCI) or dementia, such as of the Alzheimer type (DAT) (Han et al., 2015). For example, individuals with MCI may have greater difficulty in medical decision-making, specifically in reasoning and understanding, and these deficits in DM may continue to worsen over time (Okonkwo et al., 2008).

Declines in DM may be particularly salient in later stages of progressive cognitive decline caused by neurodegenerative diseases such as Alzheimer's, as these impairments affect multiple aspects of cognitive functioning (Gaubert and Chainay, 2021). Most importantly, deteriorating DM abilities can lead to adverse outcomes in daily living, including financial exploitation, mismanagement of financial resources, inability to make informed decisions about healthcare and treatment, and challenges in performing essential tasks, like driving or household management (Lai et al., 2008; Lichtenberg, 2016; Okonkwo et al., 2007).

There are several forms of decision-making, most commonly including financial decision-making, or the ability to independently manage financial tasks, and healthcare decision-making, which refers to the ability to make choices regarding one's health-related matters. The role of DM in the context of functional outcomes is of great importance as these skills are fundamental in one's ability to perform daily tasks independently. Functional outcomes refer to one's ability to carry out both basic and complex everyday tasks and are a critical metric of wellbeing and safety. They also encompass smaller-scale daily decision-making practices that require planning, reasoning, and problem-solving (Lai et al., 2008), such as preparing a meal, driving, employment skills, and engaging in social relationships. Effective measurement of DM in the context of functional outcomes is essential for clinicians looking to evaluate disease severity in adults with cognitive impairment, and assessment of complex functional skills that require intact DM abilities may help identify those with more subtle cognitive decline (Marson, 2015). It also aids in recommendations about appropriate care settings or assessing the level of caregiver support (Johnson et al., 2004). Therefore, examining the current literature to identify instruments suitable for effective measurement of functional outcomes in older adults could be of great utility for clinicians and researchers.

This scoping review is part of the larger Advancing Reliable Measurement in Cognitive Aging and Decision-making Ability (ARMCADA) study, which aims to develop and validate a suite of measures that assess multiple DM domains in older adults. The scoping review was used as a tool to identify and evaluate the most commonly used measures in research and clinical settings, and to determine the gaps in the field, with the goal of developing an efficient tool for assessing a wide range of decision-making abilities. This scoping review focused specifically on the functional outcomes domain.

## 2 Methods

This scoping review was guided by the Arksey and O'Malley (2005) methodological framework. The review methodology and results are reported in accordance with the PRISMA Extension for Scoping Reviews (PRISMA-ScR; Tricco et al., 2018). Details of the methodology are publicly available in the published protocol of the multi-domain scoping review (Ho et al., 2024). This study is classified as exempt and designated as non-human subjects research at Northwestern University (STU0U0220334).

### 2.1 Eligibility criteria

We identified studies published between January 2018 and November 2023 using keywords related to decision-making abilities and functional outcomes in aging populations. Search terms used were related to decision-making and older adults (see Ho et al., 2024 for the entire search strategy), including the comprehensive list of decision-making terms (e.g., decision-making, decisional capacity, decision quality, decisional impairment, decision process, choice making) as well as domain specific search terms for themes related to functional outcomes (e.g., functional assessment, functional status, daily living activity, independent living, social competence, full list available in Table 1). Functional outcomes were defined as skills or behaviors related to one's ability to live independently, such as competence in completing both basic and complex activities of daily living. Inclusion/exclusion criteria are listed in Table 2. Studies that only measured shared decision-making or decision aids were excluded as they were not relevant to our goal of understanding measures assessing decision -making in the context of functional outcomes. We included manuscripts with samples of adults ages 45 and over to incorporate studies that focused on the earliest signs of aging. We included empirical studies and meta-analyses. Studies that primarily used single-subject research/case studies or focus groups, as well as publications that were narrative reviews, conference proceedings, book chapters, dissertations, commentaries, pre-prints, or other non-research publications were not included. We included only publications written in English, although measures from all languages were considered.

**Table 1:** Domain specific search terms for functional outcomes.

**Search terms**
“functional assessment” “functional status assessment” “functional status” “daily life activity” “disability” “driving ability” “job adaptation” “work capacity” “home for the aged” “independent living” “assisted living facility” “nursing home” “care behavior” “community participation” “interpersonal communication” “prosocial behavior” “social adaptation” “social bonding” “social cognition” “social competence” “social disability” “social interaction” “social participation” “social responsibility” “social value”

**Table 2:** Inclusion and exclusion criteria for articles.

	**Inclusion criteria**	**Exclusion criteria**
Population	Adults over age 45 The assessment was conducted with at least one group of individuals over 45 years.	Adults ≤ 45 year old
Study Characteristics	The study mentions at least one assessment of one or more of the target domains. The domain of interest is an outcome assessed by the study. Study designs: Cohort study Case control study Randomized control trial	Single-subject research/Case studies. Focus group Review articles Narrative reviews Gray literature Conference Proceedings Books and/or book chapters Commentaries Preprints Other non-research publications
Other	Language: Measures could be in any language as long as the article is published in English Location: All geographical locations	Articles that only measure shared decision-making Articles that only measure decision aids

### 2.2 Sources of evidence and search strategy

The scoping review was conducted using *Preferred Reporting Items for Systematic Reviews and Meta-Analyses Extension for Scoping Review* (*PRISMA-Scr*; Tricco et al., 2018). A comprehensive search and article selection procedure was conducted as part of a larger review on multiple domains of DM (Ho et al., 2024) and a search strategy was developed with the help of a medical research librarian at Northwestern University's Galter Health Sciences Library. We reviewed multiple databases including Embase (Elsevier), MEDLINE (Ovid), PsycINFO (EbscoHost), Cochrane Library (Wiley), Web of Science (Clarivate), and Scopus (Elsevier). The search was conducted to capture publications available from January 1^st^, 2018, and November 6^th^, 2023.

### 2.3 Screening

Following the initial search phase, articles were screened in Covidence, a web-based collaboration platform that streamlines the production of systematic reviews (Veritas Health Innovation, 2024) using the inclusion and exclusion criteria presented in Table 2. This process was carried out in multiple stages. First, titles and abstracts were screened by two independent reviewers. Conflicts were resolved by study scientists or a third reviewer. Included articles were then re-screened using the full text to further assess eligibility. All articles that passed both screening phases advanced to the data extraction phase, where reviewers standardized the process by extracting results using a Qualtrics form (an online survey tool to build, collect, and analyze survey data; Copyright © 2024 Qualtrics, 2024). At this stage, additional articles would still be excluded if they did not meet the inclusion criteria.

### 2.4 Data extraction and synthesis

Extraction data included information about article sample characteristics (i.e., sample age group, clinical diagnosis if present, control group if present), specific measures (name used in article and standardized name used more commonly), language of the measure, administration method (remote vs. in-person, self-administered vs. technician administered), materials required for administration (computer, paper/pen, interview), reliability/validity metrics (internal consistency, interrater reliability, test-retest reliability, validity metrics) and the most relevant domain areas assessed. Institutional review board approval was not required for this study.

## 3 Results

### 3.1 Search results and article level metrics

**Figure 1:**
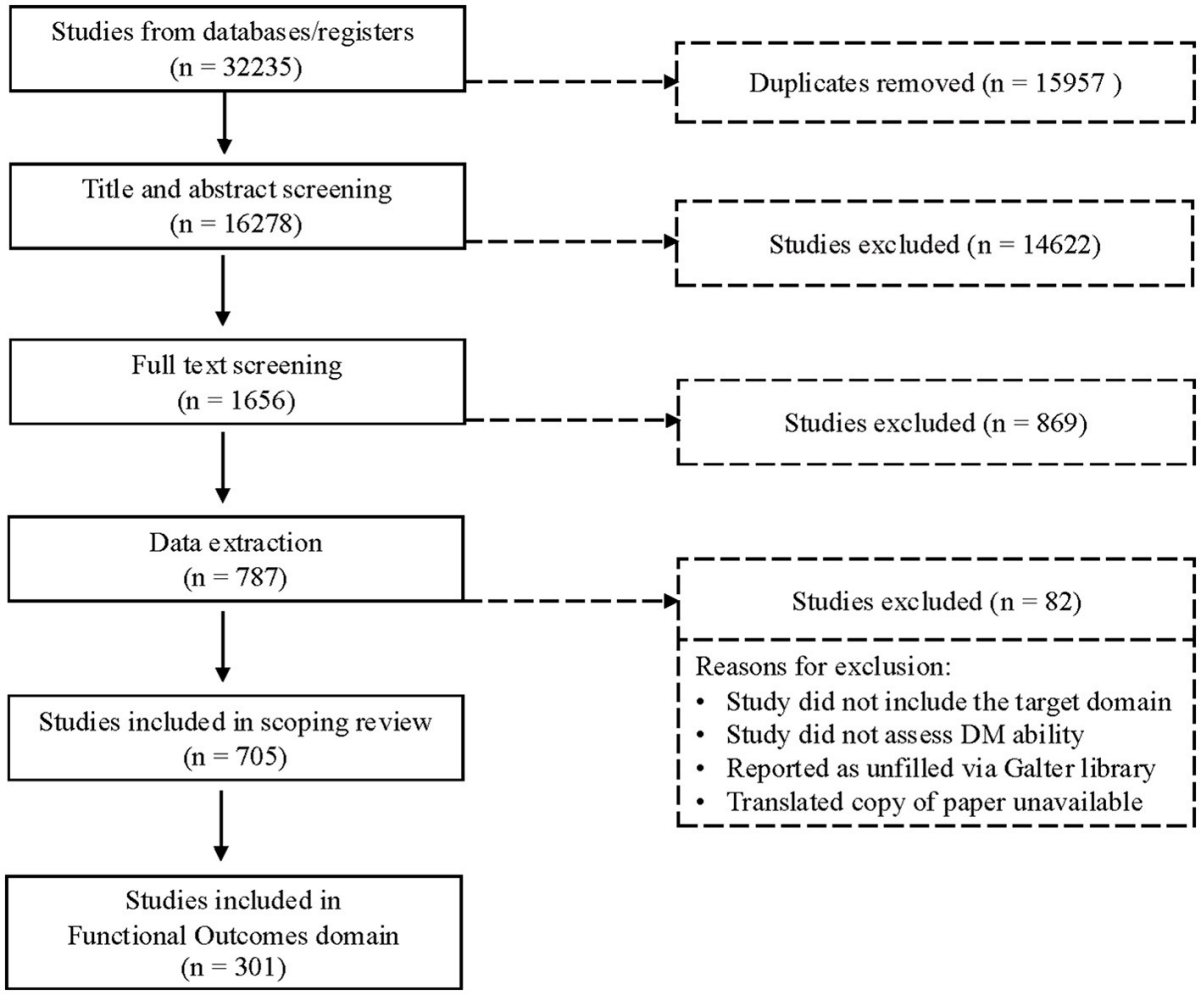
PRISMA flowchart of the article selection process.

The initial search yielded 32,235 articles (see Figure 1 for review process), including 15,957 duplicate articles, which were removed. Of the 16,278 articles left for review in the title and abstract screening, a total of 14,622 articles were excluded, and full-text screening excluded an additional 869 articles. Of the remaining 787 articles that went through extraction phase, 82 were excluded during extraction resulting in a final set of 705 articles. Of these articles, 301 focused on functional outcomes in an aging population and included 231 unique measures (450 measures used in total). The list of all articles included is presented in Supplementary Table S1, and list of all measures included in the scoping review is presented in Supplementary Table S2.

Among articles classified as assessing functional outcomes, a majority of the measures (76.4%) were administered in person. Of these, 39.2% were administered by an examiner, 45.3% were self-administered under supervision, 8.1% were self-administered without supervision, and for the rest of the in-person measures the administration method was not provided (7.3%). Approximately 14.4% of measures were administered remotely, which included 90.8% of remotely administered measures were self-administered without supervision, 4.6% were administered with an examiner, and 3.1% were self-administered with supervision. The remainder of studies (2.9%) included mixed administration (e.g., in-person and remote) or did not specify administration (6.2%). Approximately half of the studies were conducted in English (47.8%), 6% in multiple languages, 5% in Spanish, and the remainder in other languages (e.g., German, French, Dutch, Mandarin, Japanese, and Greek).

With respect to sample characteristics, all the studies included participants that were 45 years and above, though in some cases (8%) information on the age of participants was not provided but could be inferred (e.g., by the type of disease participants had such as MCI/AD, Parkinson's Disease). Approximately 92% of the studies included participants ages 45 and older only, and 64.1% included participants ages 65 and older only. Approximately 39.9% of the articles included primarily a clinical sample, while 33.2% sampled both clinical and control groups, and 26.9% did not include a clinical sample. The most common conditions of the clinical samples were a diagnosis of MCI/AD, chronic health conditions, Parkinson's disease, psychiatric or substance use disorders, and various brain lesions. Specifically, 14.6% of studies included participants along the cognitive aging spectrum of MCI to various dementias (e.g., Alzheimer's disease, frontotemporal dementia, vascular dementia, unspecified form of dementia), 6.6% included participants with Parkinson's disease, 23.9% included participants with psychiatric or substance use disorders (e.g., schizophrenia, major depressive disorder, obsessive-compulsive disorder, hoarding disorder, gambling disorder, alcohol use disorder), 16.9% with various health conditions (e.g., cancer, sleep apnea, chronic pain, heart failure), and 11.3% with neurologic conditions (e.g., epilepsy, multiple sclerosis, stroke, brain lesions).

### 3.2 Functional outcome measures

Of the 231 unique measures extracted, each was evaluated for its direct relevance to functional outcomes. The most common measures typically fell under three umbrella categories: (1) self or informant-report measures of functional abilities, (2) semi-structured interviews assessing skills such as everyday judgment, and (3) performance-based measures of functional abilities. Relevant studies and subsequent measures are listed under Table 3 and Supplementary Table S3. The most common informant-report measures related to functional outcomes included The Lawton Instrumental Activities of Daily Living (IADL) Scale (*n* = 25; Lawton and Brody, 1969) and The Barthel Index for Activities of Daily Living (ADL) Scale (*n* = 12; Mahoney and Barthel, 1965), which are brief informant- or clinician-report questionnaires that assess independence across instrumental activities of daily living (e.g., household chores, transportation, using the telephone, and managing finances) and basic activities of daily living (e.g., toileting, bathing, and mobility) respectively. The Katz Index of Independence in Activities of Daily Living (ADL) Scale (*n* = 6; Katz et al., 1963) and Functional Activities Questionnaire (FAQ; *n* = 5; Pfeffer et al., 1982) are similar measures, and focus on basic and instrumental activities of daily living respectively. The Tokyo Metropolitan Institute of Gerontology Index of Competence (TMIG-IC; Koyano et al., 1991), mentioned twice, is a self- or informant-report measure of higher-level functional capacity that covers activities of daily living, intellectual activities (e.g., reading newspapers), as well as social relationships and communication. The Amsterdam IADL Questionnaire (A-IADL; Sikkes et al., 2012) is a novel adaptive and computerized informant-report questionnaire designed to assess impairments in instrumental activities of daily living (IADL) in dementia. The A-IADL also assesses additional domains, including using technology or appliances, occupation skills, and leisure activities. This measure was included once in the scoping review.

**Table 3:** Commonly used and most relevant measures for assessing functional outcomes identified in the scoping review.

**Measure**	**% cited**	**Functional outcome(s) assessed**	**Clinical group(s) assessed**	**Format**
Lawton Instrumental Activities of Daily Living (IADL; Lawton and Brody, 1969)	8.3%	Ability to use telephone, shopping, food preparation, housekeeping, laundry, mode of transportation, responsibility for own medications, ability to handle finances.	MCI; AD; Various forms of dementia; Stroke; Various cancers (colon, breast, pancreatic, head and neck, prostate); Advanced chronic kidney disease	Semi-structured interview; Paper/pen
Barthel Activities of Daily Living (ADL; Mahoney and Barthel, 1965)	4.0%	Feeding, bathing, grooming, dressing, bowel control, bladder control, toilet use, transfers, mobility on level surfaces, stairs.	MCI; AD; Dementia; Care home residents; Various cancers (colon, head and neck, breast); Stroke; Unspecified chronic conditions	Semi-structured interview; Paper/pen
Katz Activities of Daily Living (ADL; Katz et al., 1963)	2.0%	Bathing, dressing, toileting, transferring, continence, feeding.	AD; Various cancers (head and neck cancer, colon); chronic kidney disease; Home medical care patients	Semi-structured interview
Functional Activities Questionnaire (FAQ; Pfeffer et al., 1982)	1.7%	Bills, paperwork, shopping, hobbies/skills, safety, preparing meals, keeping track of current events, paying attention to and understanding media, remembering appointments and events, traveling.	MCI; AD	Semi-structured interview
Timed Instrumental Activities of Daily Living (TIADL; Owsley et al., 2001)	1.3%	Vision-related (e.g., reading small print, identifying objects in cluttered environment), cognitive (e.g., finding number in directory), and practical (e.g., using a screwdriver) tasks.	MCI-ASD; MCI-AMD; MCI; AD	Semi-structured interview; Performance-based task
Test of Practical Judgment (TOP-J; Rabin et al., 2007)	1.0%	Safety, medical, social/ethical, and financial judgment questions.	Subjective cognitive decline; MCI; Vascular dementia (VaD); Frontotemporal dementia; Primary progressive aphasia; patients referred for neuropsychological assessments; Older adult rehabilitation inpatients	Semi-structured interview
Independent Living Scale (ILS; Loeb, 1996)	1.0%	Memory/orientation, managing money, managing home/transportation, health and safety, and social adjustment.	Various dementias; Myotonic Dystrophy Type 1 (DM1)	Semi-structured interview; Performance-based task
Tokyo Metropolitan Institute of Gerontology- Index of Competence (TMIG-IC; Koyano et al., 1991)	0.7%	Instrumental self-maintenance, intellectual activity, and social roles.	AD; Patients hospitalized with heart failure	Semi-structured interview; Paper/pen
Assessment of Capacity for Everyday Decision-making/Short Portable Version (ACED/SPACED; Lai et al., 2008)	0.7%	Understanding the functional problem, appreciating the problem, understanding the options to solve the problem, understanding the benefits and harms of options, appreciating the benefits and harms of options, and expressing a choice.	MCI	Semi-structured interview
Process Analysis of Daily Activity for Dementia (PADA-D; Tabira et al., 2019)	0.7%	Cooking, housekeeping, shopping, ability to use the telephone, laundry, use modes of transportation, managing medications, and managing finances.	AD	Semi-structured interview
Amsterdam- Instrumental Activities of Daily Living (A-IADL; Sikkes et al., 2012)	0.3%	Household duties, domestic appliances, household budget, work, computer, devices, and leisure time/other.	N/A	Computer
Texas Functional Living Scale (Cullum et al., 2001)	0.3%	Time (ability to use clocks and calendars), money and calculation, communication, and memory.	Patients referred for neuropsychological assessments	Semi-structured interview; Performance-based task
Performance Assessment of Self-Care Skills - Home (PASS-H; Holm and Rogers, 2008)	0.3%	Functional mobility, basic activities of daily living (e.g., hygiene), physical instrumental activities of daily living (e.g., lifting garbage), cognitive instrumental activities of daily living (e.g., shopping, safety)	N/A	Semi-structured interview; Performance-based task
Everyday Decision-Making Competence (EDMC; Rosi et al., 2019)	0.3%	Daily, economic, and healthcare problems.	MCI	Computer

(1) Column 2 indicates the percentage of the 301 papers included in the scoping review that reference each measure. For the frequency of each measure among the 450 total measures identified, refer to Supplementary Table S3. (2) MCI, mild cognitive impairment; AD, Alzheimer's disease; MCI-ASD, mild cognitive impairment-amnestic single domain; MCI-AMD, mild cognitive impairment- amnestic multiple domain.

Several studies included in the scoping review also utilized semi-structured interviews or measures with open-ended responses. The Test of Practical Judgment (TOP-J; *n* = 3; Rabin et al., 2007) is a brief interview-based measure that assesses judgment related to safety, medical, social/ethical, and financial issues, by asking questions that reflect real-world scenarios. Assessment of Capacity for Everyday Decision-making (ACED; *n* = 1; Lai et al., 2008) is a semi-structured interview that measures the capacity to make decisions about solving everyday problems, and assesses four decision-making abilities including understanding, appreciation, reasoning, and expressing a choice. The Everyday Decision-Making Competence task (EDMC; *n* = 1; Rosi et al., 2019) is another measure that assesses decision-making ability in everyday situations, and consists of decision-making problems about daily, economic, and healthcare scenarios.

Lastly, many studies assessed functional outcomes using performance-based measures. For example, the Timed Instrumental Activities of Daily Living (TIADL; *n* = 4; Owsley et al., 2001) assesses participants' ability to complete five different tasks where efficient completion would be advantageous including: telephone communication, financial abilities, nutrition, shopping, and medication usage. The Independent Living Scales (*n* = 3; Loeb, 1996) is a lengthier battery of measures that assesses competence in functional abilities by assessing the areas of memory/orientation, managing money, managing home and transportation, health and safety, and social adjustment; the domains include both performance-based tasks, as well as open-ended questions. The Process Analysis of Daily Activity for Dementia (PADA-D; *n* = 2; Tabira et al., 2019) is used in individuals with cognitive decline, and includes evaluation of both basic and instrumental activity performances by breaking down each activity into smaller processes and larger actions. The Texas Functional Living Scale (*n* = 1; Cullum et al., 2001) is a briefer performance-based measure, and includes domains of time, money and calculation, communication, and memory. Lastly, the Performance Assessment of Self-care Skills (PASS; *n* = 1; Holm and Rogers, 2008) is a comprehensive tool that measures daily life tasks to assist with planning occupation-based interventions across the lifespan; it covers multiple basic and activities of daily living and several mobility tasks.

## 4 Discussion

The purpose of this scoping review was to summarize the literature on existing measures of functional outcomes and aging as part of a larger study examining multiple domains of decision-making in older adults. The goal of the overall ARMCADA study is to create an efficient, comprehensive, and well-validated battery of instruments assessing DM abilities in older adults in various settings, with the ultimate vision of aiding in early identification of possible declines in decision-making and cognition. Early detection can facilitate advance planning efforts, ultimately reducing adverse outcomes and improving quality of life in potentially vulnerable groups. This scoping review examined recent measures of functional outcomes used in adults over the age of 45 with various clinical presentations including neurodegenerative disease, chronic health conditions, psychiatric illness, or neurologic disorders. The scoping review identified many studies within the domain of functional outcomes, although a deeper analysis indicated a smaller proportion contained directly relevant instruments.

The most relevant measures could be categorized into three larger categories, which included self- or informant-report questionnaires, open-ended or semi-structured interviews, and performance-based measures. Among self- or informant-report measures, most instruments assessed basic activities of daily living, including self-care, transportation, meal preparation, managing finances, managing medications, and communication. These measures can be particularly useful for brief and efficient assessment and tend to be more accurate when completed by an informant, as patients with cognitive decline may have distorted perceptions of their ability to complete certain tasks (Campbell et al., 2022; Perfect et al., 2021). While most skills covered by these measures remain relevant, others related to modern technology usage (e.g., automatic bill payment, using a cell phone or computer) are often underassessed, and novel measures should consider incorporating these abilities. Semi-structured or open-ended interview tools varied in their approach, though in general, they assessed participants' ability to solve daily and complex problems that require some degree of decision-making. The advantage of semi-structured interviews is that it allows the examiner to observe the thought processes and appreciate the qualitative subtleties underlying the decision-making process. However, limitations of these tools include inconsistent interview reliability, time burdening administration process, and the fact that responses may not reflect participants' real-time decision-making ability in day-to-day settings (Diefenbach, 2009; Karatsareas, 2022). Lastly, many studies utilized performance-based measures, where participants complete tasks such as using a telephone, paying a bill, preparing meals, or managing medications. Performance-based assessments can be particularly effective as they require participants to demonstrate real-life skills in a controlled setting, when it is not possible to assess these skills in the home or other natural environments (Harvey et al., 2007). This approach provides considerable external validity; however, these assessments are often time-consuming and may require additional materials and equipment that can be cost-prohibitive.

To our knowledge, this is the first scoping review that focuses on functional outcomes across a wide range of older adult populations without limiting the review to a specific cognitive or health condition. Previous scoping or systematic reviews have examined the effects of multimorbidity (i.e. two or more chronic health conditions) and functional decline in community-dwelling adults (Ryan et al., 2015) or specific health conditions affecting future functional decline, such as spinal cord injury (AlHuthaifi et al., 2017), hip fracture (Xu et al., 2019), or multiple sclerosis (Maggio et al., 2020). Other scoping reviews have examined the effects of specific interventions or treatments on functional abilities in older adults, such as exercise programs (Fien et al., 2022) or lifestyle interventions (Porter Starr et al., 2014). While decision-making abilities may be affected differentially across conditions, the goal of our scoping review was to survey all of the recent measures that are used across aging samples. As such, the value of the current review lies in its breadth of focus on multiple aspects of functioning across a variety of clinical presentations, though we acknowledge this approach can present a challenge if greater detail regarding a specific clinical population is needed as various clinical groups may vary dramatically in their cognitive abilities (i.e. dementia or MCI vs. chronic pain or other medical condition).

This scoping review has several limitations to consider. First, we only included published articles, excluding conference abstracts, dissertations, or other non-formally published studies (e.g., gray literature). While this exclusion may increase the potential for publication bias, it was important to focus on formally reviewed studies that have provided sufficient details about each measure, methods, and relevant validation efforts. A second limitation is that the operationalization of functional outcomes can be quite broad, meaning the selected search terms, while extensive, may not be exhaustive. While our approach in being over-inclusive led to the identification of many unrelated measures, our goal was to thoroughly investigate the current literature.

Findings from this scoping review have many important implications. First, they broaden the age range typically sampled in older adults, including participants as young as 45 to encompass the earliest stages of aging. Our review also included a broad range of clinical presentations and all languages of assessment, which increases generalizability to multiple populations. Additionally, while this scoping review focused on functional outcomes, the broader ARMCADA scoping review included multiple aspects of decision-making, resulting in a comprehensive review of key areas of decision-making ability in older adults with and without cognitive impairment.

## Data Availability

The original contributions presented in the study are included in the article/Supplementary material, further inquiries can be directed to the corresponding author.

## References

[B1] AlHuthaifi F. Krzak J. Hanke T. Vogel L. C. (2017). Predictors of functional outcomes in adults with traumatic spinal cord injury following inpatient rehabilitation: a systematic review. J. Spinal Cord Med. 40, 282–294. 10.1080/10790268.2016.1238184 27852160 PMC5472016

[B2] Arksey H. O'Malley L. (2005). Scoping studies: towards a methodological framework. Int. J. Soc. Res. Methodol. 8, 19–32. 10.1080/1364557032000119616

[B3] Boettger S. Bergman M. Jenewein J. Boettger S. (2015). Assessment of decisional capacity: prevalence of medical illness and psychiatric comorbidities. Pall. Supp. Care 13, 1275–1281. 10.1017/S1S478951514001266 25355466

[B4] Campbell R. Ju A. King M. T. Rutherford C. (2022). Perceived benefits and limitations of using patient-reported outcome measures in clinical practice with individual patients: a systematic review of qualitative studies. Qual. Life Res. 31, 1597–1620. 10.1007/s1s1136-021-03003-z 34580822

[B5] Copyright © 2024 Qualtrics (2024). The Extraction for this Paper was Generated Using Qualtrics Software, Version XM of Qualtrics. Available online at: https://www.qualtrics.com/ (accessed December 8, 2023).

[B6] Cullum C. M. Saine K. Chan L. D. Martin-Cook K. Gray K. F. Weiner M. F. (2001). Performance-based instrument to assess functional capacity in dementia: the texas functional living scale. Neuropsychiatry Neuropsychol. Behav. Neurol. 14, 103–108. 11417663

[B7] Diefenbach T. (2009). Are case studies more than sophisticated storytelling?: methodological problems of qualitative empirical research mainly based on semi-structured interviews. Qual. Quant. 43, 875–894. 10.1007/s1s1135-008-91644-0

[B8] Fellows L. K. (2004). The cognitive neuroscience of human decision making: a review and conceptual framework. Behav. Cognit. Neurosci. Rev. 3, 159–172. 10.1177/1534582304273251 15653813

[B9] Fien S. Linton C. Mitchell J. S. Wadsworth D. P. Szabo H. Askew C. D. . (2022). Characteristics of community-based exercise programs for community-dwelling older adults in rural/regional areas: a scoping review. Aging Clin. Exp. Res. 34, 1511–1528. 10.1007/s4s0520-022-02079-y 35152393 PMC8852913

[B10] Finucane M. L. Gullion C. M. (2010). Developing a tool for measuring the decision-making competence of older adults. Psychol. Aging 25, 271–288. 10.1037/a0a019106 20545413 PMC2918639

[B11] Finucane M. L. Slovic P. Hibbard J. H. Peters E. Mertz C. K. MacGregor D. G. (2002). Aging and decision-making competence: an analysis of comprehension and consistency skills in older vs. younger adults considering health-plan options. J. Behav. Decis. Making 15, 141–164. 10.1002/bdm.407

[B12] Gaubert F. Chainay H. (2021). Decision-making competence in patients with Alzheimer's disease: a review of the literature. Neuropsychol. Rev. 31, 267–287. 10.1007/s1s1065-020-094722-2 33576942

[B13] Han S. D. Boyle P. A. James B. D. Yu L. Bennett D. A. (2015). Mild cognitive impairment is associated with poorer decision-making in community-based older persons. J. Am. Geriatr. Soc. 63, 676–683. 10.1111/jgs.13346 25850350 PMC4406791

[B14] Harvey P. D. Velligan D. I. Bellack A. S. (2007). Performance-based measures of functional skills: usefulness in clinical treatment studies. Schizophr. Bull. 33, 1138–1148. 10.1093/schbul/sbm0m40 17493956 PMC2632366

[B15] Ho E. H. Ece B. Novack M. A. Pila S. Karpouzian-Rogers T. Mather M. A. . (2024). Protocol for a multi-domain scoping review to identify measures of decision-making ability in an ageing population. BMJ Open 14:e0e84178. 10.1136/bmjopen-20244-084178 39806701 PMC11664363

[B16] Holm M. Rogers J. C. (2008). The performance assessment of self-care skills (PASS). Assess. Occup. Ther. Mental Health 101–110.

[B17] Johnson N. Barion A. Rademaker A. Rehkemper G. Weintraub S. (2004). The activities of daily living questionnaire: a validation study in patients with dementia. Alzheimer Dis. Assoc. Disord. 18, 223–230. 10.1037/t2t8752-000 15592135

[B18] Karatsareas P. (2022). “Semi-structured interviews,” in Research Methods in Language Attitudes, eds. R. Kircher and L. Zipp (Cambridge University Press), 99–113. 10.1017/9781108867788.010

[B19] Katz S. Ford A. B. Moskowitz R. W. Jackson B. A. Jaffe M. W. (1963). Studies of illness in the aged. the index of ADL: a standardized measure of biological and psychosocial function. JAMA 185, 914–919. 10.1001/jama.1963.03060120024016 14044222

[B20] Koyano W. Shibata H. Nakazato K. Haga H. Suyama Y. (1991). Measurement of competence: reliability and validity of the TMIG Index of Competence. Arch. Gerontol. Geriatr. 13, 103–116. 10.1016/01677-4943(91)90053-S 15374421

[B21] Lai J. M. Gill T. M. Cooney L. M. Bradley E. H. Hawkins K. A. Karlawish J. H. (2008). Everyday decision-making ability in older persons with cognitive impairment. Am. J. Geriatr. Psychiatry 16, 693–696. 10.1097/JGP.0b00b0b13e33e3e1816c66c7cb77b5b4 18669948 PMC2730037

[B22] Lawton M. P. Brody E. M. (1969). Assessment of older people: self-maintaining and instrumental activities of daily living1. Gerontologist 9, 179–186. 10.1093/geront/9.3_Part_1.179 5349366

[B23] Lichtenberg P. A. (2016). Financial exploitation, financial capacity, and Alzheimer's disease. Am. Psychol. 71, 312–320. 10.1037/a0a040192 27159438 PMC4872660

[B24] Loeb P. (1996). Independent Living Scales (ILS) Stimulus Booklet. San Antonio: Psychological Corp.

[B25] Maggio M. G. Cuzzola M. F. Latella D. Impellizzeri F. Todaro A. Rao G. . (2020). How personality traits affect functional outcomes in patients with multiple sclerosis: a scoping review on a poorly understood topic. Mult. Scler. Relat. Disord. 46:102560. 10.1016/j.msard.2020.102560 33049463

[B26] Mahoney F. Barthel D. W. (1965). Functional evaluation; the Barthel index. A simple index of the independence useful in scoring improvement in the rehabilitation of the chronically ill. Maryland State Med. J. 14, 61–66. 14258950

[B27] Marson D. (2015). Investigating functional impairment in preclinical Alzheimer's disease. J. Prev. Alzheimers Dis. 2, 4–6. 10.14283/jpad.2015.44 26855935 PMC4743883

[B28] Okonkwo O. Griffith H. R. Belue K. Lanza S. Zamrini E. Y. Harrell L. E. . (2007). Medical decision-making capacity in patients with mild cognitive impairment. Neurology 69, 1528–1535. 10.1212/01.wnl.0000277639.90611.d9d 17923615

[B29] Okonkwo O. C. Griffith H. R. Copeland J. N. Belue K. Lanza S. Zamrini E. Y. . (2008). Medical decision-making capacity in mild cognitive impairment: a 3-year longitudinal study. Neurology 71, 1474–1480. 10.1212/01.wnl.0000334301.32358.48 18981368 PMC2676965

[B30] Owsley C. McGwin G. J. Sloane M. E. Stalvey B. T. Wells J. (2001). Timed instrumental activities of daily living tasks: relationship to visual function in older adults. Optom. Vis. Sci. 78, 350–359. 10.1097/000063244-2001050000-00019 11384013

[B31] Perfect D. Griffiths A. W. Vasconcelos Da Silva M. Lemos Dekker N. McDermid J. Surr C. A. (2021). Collecting self-report research data with people with dementia within care home clinical trials: benefits, challenges and best practice. Dementia 20, 148–160. 10.1177/1471301219871168 31466468

[B32] Pfeffer R. I. Kurosaki T. T. Harrah C. H. J. Chance J. M. Filos S. (1982). Measurement of functional activities in older adults in the community. J. Gerontol. 37, 323–329. 10.1093/geronj/37.3.323 7069156

[B33] Porter Starr K. N. McDonald S. R. Bales C. W. (2014). Obesity and physical frailty in older adults: a scoping review of lifestyle intervention trials. J. Am. Med. Dir. Assoc. 15, 240–250. 10.1016/j.jamda.2013.11.008 24445063 PMC4023554

[B34] Rabin L. A. Borgos M. J. Saykin A. J. Wishart H. A. Crane P. K. Nutter-Upham K. E. . (2007). Judgment in older adults: development and psychometric evaluation of the test of practical judgment (TOP-J). J. Clin. Exp. Neuropsychol. 29, 752–767. 10.1080/13825580601025908 17896200 PMC3482485

[B35] Rosi A. Vecchi T. Cavallini E. (2019). Metacognitive-strategy training promotes decision-making ability in older adults. Open Psychol. 1, 200–214. 10.1515/psych-20188-001427409075

[B36] Ryan A. Wallace E. O'Hara P. Smith S. M. (2015). Multimorbidity and functional decline in community-dwelling adults: a systematic review. Health Qual. Life Outcomes 13:168. 10.1186/s1s2955-015-03555-9 26467295 PMC4606907

[B37] Sikkes S. A. M. de Lange-de Klerk E. S. M. Pijnenburg Y. A. L. Gillissen F. Romkes R. Knol D. L. . (2012). A new informant-based questionnaire for instrumental activities of daily living in dementia. Alzheimers Dement. 8, 536–543. 10.1016/j.jalz.2011.08.006 23102123

[B38] Tabira T. Hotta M. Ogawa N. Murata M. Yoshiura K. Maruta M. . (2019). Development of process analysis of daily activity for dementia (PADA-D) in community-dwelling patients with dementia. Jpn. J. Geriatr. Psychiatry 30, 923–931.

[B39] Tricco A. C. Lillie E. Zarin W. O'Brien K. K. Colquhoun H. Levac D. . (2018). PRISMA extension for scoping reviews (PRISMA-ScR): checklist and explanation. Ann. Intern. Med. 169, 467–473. 10.7326/M1M8-0850 30178033

[B40] Veritas Health Innovation (2024). Covidence - Better systematic review management. Covidence. Available at: https://www.covidence.org/ (accessed October 17, 2024).

[B41] Xu B. Y. Yan S. Low L. L. Vasanwala F. F. Low S. G. (2019). Predictors of poor functional outcomes and mortality in patients with hip fracture: a systematic review. BMC Musculoskelet. Disord. 20:568. 10.1186/s1s2891-019-29500-0 31775693 PMC6882152

