# Supplementary material for: Measures of decision-making ability and functional outcomes in older adults: results from a scoping review in the ARMCADA study
Source: Front Psychol. 2025 Mar 21;16:1540493. doi: 10.3389/fpsyg.2025.1540493 (PMC11968701; doi:10.3389/fpsyg.2025.1540493)
Supplement: Supplementary file 1 [file Supplementary_file_1.docx]

Supplementary Material

Index

| **Supplementary Material** | **Page(s)** |
| --- | --- |
| Table S1. Article-level Information of articles include in scoping review | 2-13 |
| Table S2. Measure-level information of unique functional outcomes measures included in scoping review | 13-24 |
| Table S3. Most used and relevant functional outcomes measures | 25-28 |
| Table S4. PRISMA-ScR Checklist | 28-30 |
| References of all 301 articles included in functional outcomes domain of scoping review | 31-61 |

# Table S1. Article-level information of the 301 articles included in the functional outcomes domain.

| **Authors** | **Language(s)** | **Sample Age** | **Clinical Groups** |
| --- | --- | --- | --- |
| Ahmed et al., 2023 | English | 18-65 | No special group |
| Aloi et al., 2020 | Italian | 18-65 | Binge Eating Disorder/Obesity/ Healthy Controls |
| Atkinson-Clement et al., 2019 | French | 45-84 | PD (w/DBS)/ Healthy controls |
| Attridge et al., 2019 | English/ Other- not indicated | 18-74 | Chronic pain |
| Ayers et al., 2019 | English | 45-84 | Hoarding disorder |
| Baeza-Velasco et al., 2020 | French | 18-84 | MDD |
| Bakhtiari et al., 2020 | English | 54-81 | No special group |
| Bang et al., 2022 | Korean | 65-83 | Colon cancer |
| Banks et al., 2019 | English | 47-50 | No special group |
| Barch et al., 2023 | English | Not provided | Schizophrenia/ Schizoaffective disorder/Bipolar Disorder 1/ MDD/ Healthy controls |
| Becker et al., 2021 | German | 50-85 | AD/MCI/PD + normal cognition/ PD + MCI/ Healthy controls |
| Bedwell et al., 2023 | English | 25-70 | No special group |
| Berezuk et al., 2018 | English | 45-85+ | MCI |
| Bernard-Arevalo et al., 2023 | French | 18-84 | No special group |
| Best & Freund, 2018 | German | 19-80 | No special group |
| Betz et al., 2022 | English | 65-84 | No special group |
| Biernacki et al., 2023 | English | 18-84 | Nicotine addiction |
| Bjork et al., 2021 | English | 25-70 | Substance use disorder/ Healthy controls |
| Blume et al., 2018 | German | 18-60 | Obesity/ Binge eating disorder |
| Boubekri et al., 2020 | English | 21-65 | No special group |
| Bowren et al., 2018 | English | 45-84 | Focal vmPFC lesions/ Brain damage/ Healthy controls |
| Brady et al., 2021 | English | 65-96 | No special group |
| Briere et al., 2019 | French | 18-84 | Alcohol use disorder/ Healthy controls |
| Bruce et al., 2018 | English | 18-64 | Relapsing Remitting MS (RRMS) |
| Burgdorf & Amjad, 2022 | English | 65-84 | No special group |
| Burgio et al., 2022 | Italian | 56-85 | MCI |
| Buys et al., 2021 | English | 45-95 | Patients w/ stroke |
| Caballero et al., 2022 | English | Not provided | PD |
| Case & Brown, 2023 | English/ French | 18-64 | Depressed mood/ DUI convictions/ Healthy controls |
| Caudle et al., 2023 | English | 19-50 | No special group |
| Chang et al., 2020 | Chinese | 40-70 | Obstructive sleep apnea |
| Chen et al, 2018 | English | 18-85+ | No special group |
| Chiu et al., 2019 | Chinese/ Taiwanese | 50-85+ | Dementia |
| Chu et al., 2022 | Chinese | 18-84 | Post-stroke CI/ Healthy controls |
| Colón-Semenza et al., 2021 | English | 45-84 | PD/ Healthy controls |
| Compagne et al, 2023 | French | 72-97 | Mild AD |
| Croft et al., 2022 | English | 18-77 | OCD |
| Culbreth et al., 2023 | English | 18-64 | Schizophrenia/ Schizoaffective disorder/ Healthy controls |
| Curley et al., 2019 | English | 18-64 | Psychiatric disorders |
| Cuypers et al., 2018 | Dutch/ French/ German | 45-76 | Prostate cancer/Healthy controls |
| Dammann et al., 2021 | German | 18-72 | Brainstem cavernous malformations |
| Danesin et al., 2022 | English | 45-85+ | MCI/ PD/ Left Stroke/ Right Stroke/ Healthy controls |
| de Souza et al., 2022 | Portuguese | 50-92 | AD |
| de Vries et al, 2021 | Dutch | 65-84 | Head/ Neck Cancer |
| Deal et al., 2022 | English | 45-84 | PD |
| Del Missier et al., 2020 | Swedish | 60-85 | No special group |
| Dinesh et al., 2022 | English | 18-60 | Alcohol dependence w/ ADHD |
| Dos Santos et al., 2021 | French | 70-93 | Cancer |
| Drinkwater et al., 2022 | English | 18-87 | No special group |
| Elshout et al., 2018 | Dutch | 26-75 | Chiasmatic stroke |
| Emmert et al., 2021 | English | 60-93 | Dementia |
| Faulkner et al., 2021 | English | 18-64 | MDD/ Healthy controls |
| Feinstein et al., 2021 | English | 18-64 | MS |
| Fenton et al., 2023 | English | Not provided | MCI |
| Fernandez da Lama & Brenlla, 2023 | Spanish | 19-64 | No special group |
| Fernandez et al., 2021 | Spanish | 18-64 | MS/ Healthy controls |
| Flegar et al., 2021 | German | 57-71 | Low-risk prostate cancer |
| Fowler et al., 2018 | English | 65-84 | CI/ Healthy controls |
| Fowler et al., 2021 | English | 18-84 | Metastatic cancer |
| Fujiyama et al., 2022 | English | 18-84 | No special group |
| Galandra et al., 2021 | Italian | Not provided | Alcohol use disorder |
| Gareri et al., 2020 | English | 65-84 | AD |
| Gaubert et al., 2022 | French | Not provided | Alcohol dependence |
| Gaubert et al., 2023 | French | 18-87 | No special group |
| Geng et al., 2020 | Chinese | 45-85+ | AD/ MCI/ Healthy controls |
| Gerrans et al., 2021 | English | Not provided | No special group |
| Gerstenecker et al., 2023 | English | Not provided | MCI |
| Giannouli & Tsolaki, 2021 | Greek | 45-84 | Vascular Dementia/ Depression/ Healthy controls |
| Giannouli & Tsolaki, 2022 | Greek | 45-84 | No special group |
| Giannouli & Tsolaki, 2022 | Greek | 54-89 | aMCI/ Healthy controls |
| Giannouli & Tsolaki, 2022 | Greek | 65-84 | Mild AD |
| Giannouli & Tsolaki, 2023 | Greek | 65-84 | Mild AD/ aMCI |
| Giustiniani et al., 2019 | French | 21-59 | No special group |
| Gomez-Andres et al., 2019 | Spanish | 39-65 | Aneurysmal subarachnoid hemorrhage (SAH) |
| Gowey et al., 2020 | English | 32-78 | Weight loss maintainers/ re-gainers |
| Grassi et al., 2018 | Dutch | 18-65 | OCD/ Healthy controls |
| Grassi et al., 2020 | Italian | 18-65 | OCD and GD |
| Gregorio et al., 2020 | Portuguese | 20-96 | No special group |
| Gu et al., 2022 | English | 45-85+ | AD/ LBD/ AD + LBD |
| Gungor et al., 2018 | Turkish | 18-65 | OCD/ Healthy controls |
| El Haj & Moustafa, 2023 | French | 45-84 | Korsakoff's syndrome/ Healthy controls |
| Hall et al., 2022 | English/ French | 18-54 | No special group |
| Hassani et al., 2022 | English | 60-84 | Pelvic organ prolapse surgery patients |
| Hayden et al., 2020 | English | 21-60 | No special group |
| Hegedus et al., 2021 | Hungarian | 18-65 | MDD/ Suicide attempters |
| Heim et al., 2020 | German | 18-84 | HD/ Healthy controls |
| Heintz et al., 2023 | English | 45-84 | No special group |
| Hidrus et al., 2021 | Malay | 18-84 | Type 2 diabetes mellitus |
| Ho et al., 2018 | Chinese | 20-60 | MDD |
| Hodgson et al., 2021 | English | 56-100 | Care home residents |
| Horne et al., 2020 | English | 45-84 | No special group |
| Hou et al., 2023 | Chinese | 18-85+ | Bladder cancer |
| Huang & Yang, 2023 | Taiwanese | 65-85+ | MCI/ Healthy controls |
| Husain et al., 2021 | English | 21-50 | MDD |
| Ikeda et al., 2019 | Japanese | 45-85+ | No special group |
| Imai et al., 2020 | Japanese | 45-85+ | ALS/ Healthy controls |
| Irmen et al., 2019 | German | 52-75 | Idiopathic PD/ Healthy controls |
| James et al., 2018 | English | 66-100 | No special group |
| Jamieson et al, 2020 | English/ Other- not indicated | 65-85+ | Heart failure |
| Jebraeili et al., 2021 | Persian | 18-64 | No special group |
| Jehu et al., 2022 | English | 70-85+ | No special group |
| Karamaouna et al., 2023 | Greek | 18-57 | Schizotypal/ Healthy controls |
| Karyadi et al., 2022 | English | 21-85 | Psychiatric inpatients |
| Kim et al., 2020 | English | 40-75 | Aphasia (Anomic/Wernicke's/Broca's)/ Healthy controls |
| Kolva et al., 2020 | English | 50-89 | Advanced cancer |
| Kreis et al., 2022 | German | 18-65 | Schizophrenia/ Healthy controls |
| Lai et al., 2023 | English | Not provided | Cerebral ataxia |
| Lande et al., 2022 | English | 26-102 | No special group |
| Lau et al., 2023 | Chinese/ Taiwanese | 20-65 | Chronic migraine/ Chronic migraine + medication overuse/ Healthy controls |
| Lebeau et al., 2022 | English | 45-84 | No special group |
| Lee et al., 2020 | Korean | 65-85+ | Dementia |
| Leo et al., 2019 | Italian | 65-84 | Metastatic breast cancer |
| Leon et al., 2023 | Spanish | Not provided | Impulsive compulsive diagnoses |
| Li et al., 2022 | German | 75-85+ | Gastrointestinal malignancy |
| Li et al., 2022 | German | 75-88 | Colorectal cancer |
| Li et al., 2022 | English | 18-86 | No special group |
| Lichtenberg et al, 2021 | English | Not provided | No special group |
| Limbrick-Oldfield et al., 2020 | English | 20-58 | GD/ Healthy controls |
| Ljunggren et al., 2023 | Swedish | 20-64 | Epilepsy |
| Lloyd et al., 2020 | English | 45-84 | Idiopathic PD |
| Logge et al., 2023 | English | 29-68 | Alcohol use disorder |
| Lohse et al., 2023 | Danish | 18-52 | No special group |
| Lotvonen et al., 2018 | Finnish | 59-93 | No special group |
| Lu, 2023 | Chinese | 45-85+ | No special group |
| Ma et al., 2021 | English | 18-62 | OCD/ Healthy controls |
| Mallorquí-Bagué et al., 2018 | Spanish | 18-65 | GD |
| Manguia et al., 2021 | Spanish | 18-64 | Binge eating disorder |
| Mao et al., 2023 | English | 21-50 | Sleep deprivation |
| Marazia et al., 2022 | Not provided | 18-65 | Schizophrenia/ Schizoaffective Disorder |
| Marques et al., 2022 | French | 45-84 | PD with and without REM sleep behavior disorder/ Healthy controls |
| Martin et al., 2019 | English | Not provided | MCI/ Mild AD/ Healthy controls |
| Martini et al., 2018 | English | 45-85 | PD/ Impulse Control Disorder/ Healthy controls |
| Marton et al., 2019 | English | 18-64 | Obsessive Compulsive Disorder/ Healthy controls |
| Maurage et al., 2018 | French | Not provided | Alcohol use disorder |
| McCarthy et al., 2018 | English | 65-85+ | Patients w/ tumors |
| McDermott et al., 2022 | English | 18-64 | Anxiety/ Depression/ Healthy controls |
| McWilliams et al., 2021 | English | 40-79 | No special group |
| Metz et al., 2018 | Dutch | 18-83 | No special group |
| Millroth et al., 2019 | English/ Swedish | 18-75 | No special group |
| Mimmack et al., 2023 | English | Not provided | MCI/ Healthy controls |
| Moccia et al., 2021 | Italian | 18-65 | GD/ Healthy controls |
| Moore et al., 2023 | English | 18-80 | No special group |
| Moran et al., 2023 | English | 18-64 | Schizophrenia/ Schizoaffective Disorder/ Healthy controls |
| Moreira et al., 2022 | Portuguese | 57-85 | MCI/Healthy controls |
| Moro et al., 2020 | Italian | 65-85 | AD/ Healthy controls |
| Moses-Payne et al., 2019 | English | 18-71 | No special group |
| Moynihan et al, 2018 | English | 22-73 | Schizophrenia/ Schizoaffective disorder |
| Mueller et al, 2019 | Spanish | 45-85+ | Mild AD/ Healthy controls |
| Mukherjee et al., 2020 | English | 18-84 | MDD/ Healthy controls |
| Muller et al., 2021 | German | 18-52 | No special group |
| Munir et al., 2022 | English | 65-89 | Non-metastatic breast cancer |
| Muslemani et al., 2022 | French | 23-57 | Myotonic dystrophy type 1 (DM1) |
| Nakamizo et al., 2020 | Japanese | 45-85+ | Patients w/ history of carotid endarterectomy |
| Nakao et al., 2020 | Japanese | 36-47 | HIV/ Healthy controls |
| Newton et al., 2023 | English | 45-84 | Arthritis and associated conditions |
| Nigro et al., 2021 | English | 22-51 | Cocaine users with HIV/ Cocaine users/ HIV/ Healthy controls |
| Noaman et al., 2019 | English | 60-85+ | No special group |
| Novais et al., 2021 | French | 65-84 | Advanced chronic kidney disease |
| Nowrangi et al., 2022 | English | 65-85+ | MCI/ Healthy controls |
| Noyes et al., 2022 | English | 18-84 | Patients w/ cancer |
| Nurmi et al., 2023 | English/ Spanish | 21-50 | Impulse control disorder/ Healthy controls |
| Oba et al., 2021 | Japanese | 64-94 | AD |
| Occhiogrosso et al., 2020 | English | 53-77 | Malignant Gliomas |
| Ojala et al., 2018 | Dutch | 18-52 | GD/ Healthy controls |
| Olie et al., 2023 | Spanish | 18-75 | Suicide attempters/ Healthy controls |
| Oliveira et al., 2018 | Danish | 18-84 | No special group |
| Ord et al., 2019 | English | 56-95 | CI |
| Orduz-Bastidas et al., 2020 | Spanish | 45-84 | PD/ Healthy controls |
| Ostendorf et al., 2020 | German | 16-56 | No special group |
| Ouerchefani et al., 2019 | English | 19-59 | Brain lesions (VMPFC/DLPFC)/ Healthy controls |
| Ouerchefani et al., 2022 | French | 19-57 | Frontal lobe lesions/ Healthy controls |
| Ouerfelli-Ethier et al., 2018 | English/ French | 19-85 | Mild to moderate idiopathic PD/ Healthy controls |
| Ozcan et al., 2020 | Turkish | 18-65 | Treatment resistant MDD |
| Ozdemir et al., 2023 | English/ Chinese/ Malay | 45-84 | Heart failure |
| Page et al., 2022 | English | 18-85+ | Chronic condition |
| Yusupova et al., 2023 | Uzbek/ Russian | 30-75 | Arterial hypertensions (grades 1-3) |
| Parr et al., 2023 | English | 43-85 | PD/ Healthy controls |
| Passler et al., 2020 | English | 45-85+ | No special group |
| Patai et al., 2022 | English | 44-70 | PD |
| Patt et al., 2023 | English | 51-79 | Hippocampal lesions/ Healthy controls |
| Paul et al., 2021 | English | 55-80 | HIV |
| Perez et al., 2018 | English | 28-79 | No special group |
| Pettorruso et al., 2019 | Italian | 18-65 | GD |
| Phan et al., 2023 | Vietnamese | 18-65 | No special group |
| Pikouli et al., 2023 | Greek | 62-80 | MCI |
| Prettyman et al., 2021 | English | 19-54 | Schizophrenia/ Healthy controls |
| Pugliese & Senne, 2018 | Portuguese | 27-58 | No special group |
| Pushkarskaya et al., 2018 | English | 18-64 | Hoarding Disorder/ Healthy controls |
| Quan et al., 2022 | English | 21-50 | No special group |
| Quinn et al., 2018 | English | Not provided | Rehab (PT) inpatients |
| Rabin et al., 2022 | English | 45-85+ | Subacute combined degeneration (SCD)/ MCI/ VaD/ AD/ bvFTD/ Mixed comorbidities |
| Raina et al., 2021 | English | Not provided | PD/ Healthy controls |
| Rajan et al., 2018 | English | Not provided | PD |
| Ramchandram et al., 2020 | English | 26-61 | Psychosis spectrum (PPS) |
| Ramchandran et al., 2020 | English | 55-89 | No special group |
| Reddy et al., 2018 | English | 18-60 | Schizophrenia |
| Renz & Lincoln, 2022 | German | 18-65 | Psychotic disorders |
| Rinaldi et al., 2020 | French | 18-60 | MDD |
| Rodriguez & Ellis, 2018 | English | 50-85 | No special group |
| Rogge, 2022 | Dutch/ French/ German | 18-72 | Autism/ Healthy controls |
| Ronneikko et al., 2023 | Finnish | 80-104 | Patients hospitalized between 1/2014-12/31/2015 |
| Ronnlund et al., 2019 | Swedish | 60-90 | No special group |
| Rosario et al., 2019 | English | 65-85 | Cancer diagnosis |
| Rosi et al., 2019 | Italian | 20-85 | No special group |
| Ruppin et al., 2023 | English | 19-69 | No special group |
| Saleh et al., 2021 | English | 45-85+ | Small Vessel Cerebrovascular Disease |
| Saleh et al., 2023 | English | 18-84 | Schizophrenia/ Healthy controls |
| Sandor et al., 2022 | Turkish | 18-64 | Mesial temporal lobe epilepsy w/ hippocampal sclerosis/ Posterior cortex epilepsy/ Healthy controls |
| Santos et al., 2022 | Portuguese | 45-85+ | Mild to moderate AD |
| Santoyo-Mora et al., 2022 | Spanish | 19-64 | COVID-19/ Healthy controls |
| Saragih et al., 2022 | English/ Dutch/ Korean/ Chinese/ Slovakian/ Taiwanese | 45-85+ | AD/ MCI/ Some form of dementia |
| Sasaki et al., 2023 | Japanese | 20-70 | Alcohol use disorder |
| Schellenberg & Bailis, 2018 | English | 18-65 | No special group |
| Schmicker et al., 2019 | German | 60-75 | No special group |
| Schmitz et al., 2021 | English | 18-55 | Cocaine-use disorder |
| Segura-Serralta et al., 2020 | Spanish | 18-64 | Eating disorders/ Healthy controls |
| Sehrig et al., 2021 | German | Not provided | Alcohol use disorder |
| Serra et al., 2020 | Italian | 18-84 | Myotonic Dystrophy Type-1/ Healthy controls |
| Seubert-Ravelo et al., 2021 | Spanish | 45-84 | Early-onset PD/ Healthy controls |
| Sharma et al., 2020 | English | 45-84 | Acute ischemic stroke |
| Sharman et al., 2019 | English | Not provided | GD |
| Shaverdian et al., 2018 | English | 44-87 | Prostate cancer |
| Shepherd et al., 2022 | English | 18-84 | No special group |
| Shi et al., 2020 | English | 65-85+ | No special group |
| Sinclair et al., 2021 | English | 72-78 | No special group |
| Sinclair et al., 2023 | English | 72-78 | MCI/Healthy controls |
| Slaughter et al., 2019 | English | 18-84 | Primary intracerebral hemorrhage |
| Smith et al., 2020 | English | 18-55 | Substance use disorder/ Healthy controls |
| Smith et al., 2021 | English | 18-55 | Depression/ Anxiety/ Substance use disorder/ Healthy controls |
| Smith et al., 2022 | English | 18-80 | Brain lesions/ Healthy controls |
| Sobkow et al., 2020 | Polish | 18-55 | No special group |
| Song et al., 2019 | English | 45-85+ | Dementia |
| Spataro & La Bella, 2021 | Italian | 54-68 | ALS/ Healthy controls |
| Spohn et al., 2022 | English | 18-75 | No special group |
| Steffen et al., 2023 | German | 17-75 | No special group |
| Steward et al., 2019 | English | 54-88 | MCI-ASD and MCI-AMD |
| Steward et al., 2019 | English | 54-88 | MCI/ Mild dementia |
| Steward et al., 2019 | English | 54-88 | MCI |
| Stewart et al., 2018 | English | Not provided | No special group |
| Stolz et al., 2021 | German | 18-84 | Narcissistic personality disorder/ Healthy controls |
| Strikwerda-Brown et al., 2021 | English | Not provided | bvFTD/ AD/ Healthy controls |
| Suetani et al., 2022 | English | 18-50 | Psychotic disorder/ Healthy controls |
| Sugden et al., 2019 | English | 18-55 | No special group |
| Sullivan et al., 2023 | English | 18-77 | No special group |
| Sulu et al., 2023 | English | 18-65 | Cushing's disease |
| Sun et al., 2020 | Mandarin | 55-85+ | AD/ MCI/ Healthy controls |
| Szanto et al., 2018 | English | 50-87 | Depression/ Suicide attempters/ Healthy controls |
| Tabira et al., 2022 | Japanese | 65-85+ | AD/ Healthy controls |
| Tannou et al., 2021 | English | 58-95 | PD |
| Tarantino et al., 2021 | Italian | 19-69 | No special group |
| Testa et al., 2021 | Spanish | 18-64 | Obesity/ Anorexia/ GD/ Healthy controls |
| Thiebaut et al., 2019 | French | 15-66 | Eating Disorders |
| Thrailkill et al., 2022 | English | 18-64 | Nicotine addiction/ Healthy controls |
| Tikasz et al., 2019 | English/ French | 18-64 | Schizophrenia/ Schizoaffective Disorder/ Healthy controls |
| Toi et al., 2022 | English/ French | 65-85+ | No special group |
| Tolbert et al., 2019 | English | 55-90 | MCI/ AD/ Healthy controls |
| Torroella Carney et al., 2018 | English | Not provided | No special group |
| Tsuda et al., 2019 | Japanese | 85+ | Home medical care patients |
| Urbiete et al., 2020 | Lithuanian | 65-85+ | No special group |
| van den Berg et al., 2020 | Dutch | 18-84 | AD/ FTD/ HD/ LBD/ Healthy controls |
| van den Berg et al., 2020 | Dutch | 43-76 | Stroke |
| van den Berk-Clark et al., 2018 | English | 19-64 | Psychological trauma/ Healthy controls |
| Ozdeniz Varan & Gurvit, 2023 | Turkish | 18-50 | Prolactinoma |
| Vella et al., 2018 | English | 16-65 | Autism Spectrum Disorder/ Healthy controls |
| Verrijp et al., 2022 | Dutch | 18-97 | No special group |
| Verveer et al., 2020 | Dutch | 18-65 | Cocaine users |
| Vila-Ballo et al., 2022 | Spanish | 21-71 | Epilepsy/ Healthy controls |
| Wadley et al., 2020 | English | 45-85+ | MCI |
| Wagenbreth et al., 2019 | German | 36-74 | PD |
| Walker and Brown, 2018 | English/ Portuguese/ Farsi | 18-84 | HIV/ Healthy controls |
| Wang et al., 2021 | Chinese | 20-65 | DLPFC tumors/ VPFC tumors/ Healthy controls |
| Wang et al., 2022 | Chinese | Not provided | Meth use disorder |
| Wei et al., 2022 | Mandarin/ Taiwanese | 20-84 | Some type of health condition/ Healthy controls |
| Weller et al., 2019 | English | 24-84 | No special group |
| Weygandt et al., 2018 | German | 18-84 | MS/ Healthy controls |
| Weygandt et al., 2019 | German | 21-64 | MS/ Healthy controls |
| Wheaton & Topilow, 2020 | English | 18-84 | No special group |
| Wild et al., 2022 | English | 70-85+ | No special group |
| Wilson & Vassileva, 2018 | Bulgarian | 18-50 | Abstinent substance users/ Healthy controls |
| Wilson et al., 2023 | English | 20-83 | Chronic lower back pain |
| Wolfling et al., 2020 | German | Not provided | GD/ Internet GD |
| Wong & Chow, 2021 | Chinese | 18-65 | Mild intellectual disabilities |
| Wu et al., 2018 | English | 18-70 | Autism/ Healthy controls |
| Wyld et al., 2021 | English | 69-102 | Breast cancer/ Healthy controls |
| Xu et al., 2021 | Chinese/ Taiwanese | 20-69 | Schizophrenia/ Schizoaffective Disorder/ Healthy controls |
| Yamashita et al., 2020 | Japanese | 65-85+ | Patients w/ heart failure |
| Yang et al., 2021 | Chinese | 60-85+ | Chronic conditions |
| Yazdi et al., 2019 | English | 24-57 | No special group |
| Ye et al., 2020 | Chinese | 50-80 | Age-related cataract/ Healthy controls |
| Yeo et al., 2021 | English/ Mandarin | 45-84 | No special group |
| Yildirim et al., 2020 | Turkish | 45-84 | PD/ Healthy controls |
| Yuan et al., 2022 | Chinese | 60-85+ | No special group |
| Zakrzewski et al., 2020 | English | 18-85+ | Hoarding Disorder |
| Zamarian et al., 2020 | German | 30-55 | MS/ Healthy controls |
| Zhang et al., 2022 | Chinese | 60-85+ | MCI/ Healthy controls |
| Zhang et al., 2022 | English | 23-63 | Chronic pain/ Healthy controls |
| Zhao et al., 2023 | Chinese | 24-65 | Obstructive sleep apnea syndrome/ Healthy controls |
| Zhao et al., 2023 | Chinese | 45-84 | No special group |
| Zhao et al., 2023 | Chinese | 60-118 | No special group |
| Zhu et al., 2018 | Chinese | 70-88 | No special group |
| Zilbershlag & Josman, 2019 | English/ Hebrew | 65-92 | No special group |

*Note. AD= Alzheimer’s Disease; FTD= Frontotemporal Dementia; HD= Huntington’s Disease; MCI= Mild Cognitive Impairment; CI= Cognitive Impairment; OCD= Obsessive Compulsive Disorder; GD= Gambling Disorder; PD= Parkinson’s Disease; MS= Multiple Sclerosis; vmPFC= ventromedial Prefrontal Cortex; DLPFC= Dorsolateral Prefrontal Cortex; MDD= Major Depressive Disorder; LBD= Lewy Body Dementia; ADHD= Attention Deficit Hyperactivity Disorder; MCI-ASD= Amnestic Single-domain Mild Cognitive Impairment; MCI-AMD= Amnestic Multiple-domain Mild Cognitive Impairment; VaD= Vascular Dementia; bvFTD= behavioral variant Frontotemporal Dementia; ALS= Amyotrophic Lateral Sclerosis.*

# Table S2. Measure-level information of 232 unique measures included in the functional outcomes domain (450 total).

| **Measure** | **Original Citation** | **In-person vs. Remote** | **Administration** | **Technology** |
| --- | --- | --- | --- | --- |
| 10-meter Walk Test | Kim et al., 2016 | In-person | Administered by an examiner/researcher/clinician | Not provided |
| Activities of Daily Living Hierarchy Scale (ADLHS) | Morris et al., 2013 | In-person | Administered by an examiner/researcher/clinician | Not provided |
| Adelaide Driving Self-Efficacy Scale (ADSES) | George et al., 2007 | In-person | Self-administered with no supervision | Not provided |
| Adherence Determination Questionnaire- modified | DiMatteo et al. 1993 | Remote | Self-administered with no supervision | Computer |
| Adjusting Amount Discounting Task | Du et al., 2002 | In-person | Not provided | Not provided |
| Adult Decision-making Competence Scale (A-DMC) (n=7) | Bruine de Bruin et al., 2007 | Hybrid | Self-administered with no supervision | Computer; Paper/pen |
| Adults and Older Adults Functional Assessment Inventory | Sousa et al., 2008 | In-person | Administered by an examiner/researcher/clinician | Not provided |
| Advance Care Planning Engagement Survey (ACP-ES)- modified to (ACP-TS) | Sudore et al., 2017 | In-person | Not provided | Not provided |
| Ambiguity Tolerance Task (Mukherjee et al., 2020) | Not provided | In-person | Self-administered under supervision | Computer |
| Amsterdam Instrumental Activities of Daily Living Questionnaire (A-IADL-Q) | Sikkes et al., 2012 | Remote | Self-administered with no supervision | Computer |
| Approach-Avoidance Conflict (AAC) Task (n=2) | Not provided | In-person | Self-administered under supervision | Computer |
| Assessment of Capacity for Everyday Decision Making (ACED) | Lai & Karlawish, 2007 | In-person | Administered by an examiner/researcher/clinician | Not provided |
| Assessment of Capacity for Everyday Decision Making- Shortened Version (SPACED) | Lai & Karlawish, 2007 | In-person | Administered by an examiner/researcher/clinician | Not provided |
| Attribute-Rating Task | Not provided | In-person | Self-administered under supervision | Computer |
| Balloon Analogue Risk Task (BART) (n=10) | Lejuez et al., 2002 | In-person | Self-administered under supervision | Computer |
| Balloon Task | Gold et al., 2013 | In-person | Self-administered under supervision | Computer |
| Bandit Task | Zhang and Yu, 2013 | In-person | Self-administered under supervision | Computer |
| Beads Task (n=2) | Djamshidian et al., 2012 | In-person | Administered by an examiner/researcher/clinician; Self-administered under supervision | Computer |
| Bedside Capacity Assessment Tool (BCAT) | Grisso & Appelbaum, 1988 | In-person | Administered by an examiner/researcher/clinician | Not provided |
| Behavior Rating Inventory of Executive Function - Adult (BRIEF-A) | Roth et al., 2005 | Not provided | Self-administered with no supervision | Not provided |
| Berg Balance Scale (BBS) (n=2) | Bilney et al., 2003 | In-person | Administered by an examiner/researcher/clinician | Not provided |
| Black Swan Decision Tasks | Perfors & van Dam, 2018 | Remote | Self-administered with no supervision | Computer |
| Blessed- Dementia Scale (BLS-D) | Blessed et al., 1968 | In-person | Administered by an examiner/researcher/clinician | Not provided |
| Cambridge Gambling Task (CGT) (n=8) | Rogers et al., 1999 | In-person | Self-administered under supervision | Computer |
| Capacity to Consent to Treatment Instrument (CCTI) | Marson et al., 1995 | In-person | Administered by an examiner/researcher/clinician | Not provided |
| CAPER TREATMENT Survey | Not provided | Remote | Self-administered with no supervision | Computer |
| Cards and Lottery Task (CLT) | Müller et al., 2017 | Remote | Self-administered with no supervision | Computer |
| Career State Inventory (CSI) | Leierer et al ., 2017 | Remote | Self-administered with no supervision | Computer |
| Career Thoughts Inventory (CTI) | Sampson et al ., 1996 | Remote | Self-administered with no supervision | Computer |
| Choice Task | Not provided | In-person | Self-administered under supervision | Computer |
| Choice Task of Dell Computers | Not provided | Remote | Self-administered with no supervision | Computer |
| Clinical Dementia Rating (CDR) | Morris, 1993 | In-person | Administered by an examiner/researcher/clinician | Not provided |
| Cognistat | Matsuda et al., 2004 | In-person | Administered by an examiner/researcher/clinician | Not provided |
| Cognitive Abilities Screening Instrument (CASI) | Teng et al., 1994 | In-person | Administered by an examiner/researcher/clinician | Not provided |
| Cognitive Effort Discounting (COGED) Task | Westbrook et al., 2013 | In-person | Self-administered under supervision | Computer |
| Color Judgment Task | Sarig et al., 2012 | Remote | Self-administered with no supervision | Computer |
| Columbia Card Task (CCT) | Figner & Voelki, 2004 | In-person | Self-administered under supervision | Computer |
| Combined Scale for Proxy Informed Consent Decisions (CONCORD) Scale | Shepherd et al., 2022 | Remote | Self-administered under supervision | Computer |
| Confidence Task | Not provided | Remote | Self-administered with no supervision | Computer |
| Consideration of Future Consequences Scale (CFC) (n=2) | Strathman et al., 1994 | In-person | Administered by an examiner/researcher/clinician; Self-administered with no supervision | Paper/pen |
| Culture Fair Task | Institute for Personality and Ability Testing, 1973 | In-person | Administered by an examiner/researcher/clinician | Not provided |
| Daily Living of Older Adults with Disabilities (DIDLD) | Sagari et al., 2020 | In-person | Administered by an examiner/researcher/clinician | Not provided |
| Decision-making Quality Index- Financial Competence | Ambuehl et al., 2014 | Remote | Self-administered with no supervision | Computer |
| Decision Making Tendency Inventory (DMTI) | Misuraca et al., 2015 | Remote | Self-administered with no supervision | Computer |
| Decision Readiness | Not provided | Hybrid | Self-administered with no supervision | Paper/pen |
| Decision Regret Scale (DRS) (n=6) | Brehaut et al., 2003 | Hybrid | Administered by an examiner/researcher/clinician; Self-administered with no supervision | Computer; Paper/pen |
| Decision to Drive (2 Scenarios) | Case & Brown, 2023 | In-person | Administered by an examiner/researcher/clinician | Not provided |
| Decisional Balance (DB) Scale | Plotnikoff et al., 2001 | Remote | Self-administered with no supervision | Paper/pen |
| Decisional Conflict Scale (DCS) (n=8) | O'Connor, 1995 | Hybrid | Administered by an examiner/researcher/clinician; Self-administered with no supervision | Computer; Telephone; Paper/pen |
| Decision-Making Patterns in (Dis)Agreement Situations | Wong et al., 2021 | In-person | Administered by an examiner/researcher/clinician | Not provided |
| Decision-Making Questionnaire (DMQ) | French et al., 1993 | Remote | Self-administered with no supervision | Computer |
| Social Cognition (COGSOC) Battery | Hernandez-Galvan & Yanez-Tellez, 2013 | In-person | Administered by an examiner/researcher/clinician | Not provided |
| Deck Choice Effort Task (n=2) | Reddy et al., 2015 | Hybrid | Administered by an examiner/researcher/clinician | Computer |
| Delay Discounting Task (DDISC) | Westbrook et al., 2013 | Remote | Self-administered with no supervision | Computer |
| Delay Discounting Task (DDT) (n=4) | Koffarnus & Bickel, 2014; Richards et al., 1999; Kirby et al., 1999 | Hybrid | Self-administered with no supervision; Self-administered under supervision | Computer |
| Dependence Scale (DS) | Stern et al., 1994 | In-person | Administered by an examiner/researcher/clinician | Not provided |
| Die Roll Decision-making Paradigm | Irmen et al., 2019 | In-person | Self-administered under supervision | Computer |
| Domain-Specific Risk-Taking Scale (DOSPERT) | Weber et al., 2002 | Remote | Self-administered with no supervision | Computer |
| DriveABLE Cognitive Assessment Tool (DCAT) | Dobbs et al., 1998 | In-person | Self-administered under supervision | Tablet |
| DriveABLE On-Road Evaluation (DORE) | Dobbs et al., 1998 | In-person | Administered by an examiner/researcher/clinician | Vehicle |
| Driving Behavior Questionnaire (DBQ) | Dula & Ballard, 2003 | Remote | Self-administered with no supervision | Computer |
| Driving Habits Questionnaire (DHQ) (n=2) | Owsley et al., 1999 | In-person | Self-administered under supervision; Self-administered with no supervision | Computer |
| Driving Scenarios Task | Andrews & Westerman, 2012 | In-person | Self-administered with no supervision | Computer |
| Driving Simulator Task | Dotzauer et al., 2013 | In-person | Self-administered under supervision | Computer |
| Dundrum Capacity Ladders (DCL) | Moynihan et al., 2018 | In-person | Self-administered with no supervision | Paper/pen |
| Eastern Cooperative Oncology Group (ECOG) Performance Status (n=3) | Oken et al., 1982 | In-person | Administered by an examiner/researcher/clinician | Paper/pen |
| Effort Expenditure for Reward Task (EEfRT) - modified | Treadway et al., 2009 | In-person | Self-administered under supervision | Computer/Stationary bike |
| Effort-based decision-making (EBDM) Apple Picking Task | Not provided | In-person | Self-administered under supervision | Computer |
| Emotional Arousal and Decision Strategies Questionnaire | Not provided | In-person | Self-administered under supervision | Computer |
| Emotional Go-No/Go Task (EGNG) (n=2) | Egashira et al., 2015; Tottenham et al., 2011 | In-person | Self-administered under supervision | Computer |
| Estate Planning Documents Questionnaire (EPDQ) | Sullivan et al., 2023 | Remote | Self-administered with no supervision | Computer |
| European Organisation for Research and Treatment of Cancer- QoL Questionnaire (EORTC- QLQC30) (n=2) | Aaronson et al., 1993 | Hybrid | Self-administered with no supervision | Paper/pen |
| EuroQol 5-dimensional questionnaire (EQ5D-5L) | Herdman et al., 2011 | Hybrid | Self-administered with no supervision | Tablet |
| Everyday Decision-making Competence task (EDMC) | Rosi et al. 2019 | Remote | Self-administered under supervision | Computer |
| Expanded Judgment Task | Leimbach et al., 2018 | In-person | Administered by an examiner/researcher/clinician | Computer; Whole-head magnetoencephalography (MEG) System |
| Experimental Effort Based Task | Atkinson-Clement et al., 2019 | In-person | Administered by an examiner/researcher/clinician | Tablet; Pressure bulb |
| Failure to Minimize Risk Assessment | Not provided | Remote | Self-administered with no supervision | Computer |
| Falls Efficacy Scale International (FES) Questionnaire | Figueiredo & Neves, 2018 | In-person | Not provided | Not provided |
| Fatigue Impact Scale (FIS) | Fisk et al., 1994 | In-person | Administered by an examiner/researcher/clinician | Not provided |
| Financial Capacity Instrument (FCI) (n=2) | Marson et al., 2000 | In-person | Administered by an examiner/researcher/clinician | Not provided |
| Financial Capacity Instrument-Short Form (FCI-SF) (n=7) | Marson et al., 2000; Gerstenecker et al., 2016 | In-person | Administered by an examiner/researcher/clinician | Not provided |
| Financial Competence Measure | Ambuehl et al., 2014 | Remote | Self-administered with no supervision | Computer |
| Financial Decision Tracker (FDT) | Lichtenberg et al., 2015 | In-person | Administered by an examiner/researcher/clinician | Not provided |
| Financial Judgment Scale | Earl et al., 2015 | In-person | Self-administered with no supervision | Computer |
| Financial Risky Choice Paradigm | De Martino et al., 2006 | In-person | Self-administered under supervision | Computer |
| Flashing Grid Task | Garton et al., 2019 | In-person | Self-administered under supervision | Computer |
| fMRI Effort-Discounting Task (EDT) | Not provided | In-person | Administered by an examiner/researcher/clinician | Computer; fMRI |
| fMRI Reward Task | Clark et al., 2009 | In-person | Self-administered under supervision | Computer; fMRI |
| Forced Choice Problems Task | Not provided | Remote | Self-administered with no supervision | Computer |
| Forced-Choice Preference Task | Not provided | In-person | Administered by an examiner/researcher/clinician | Computer |
| FRAIL Scale | Morley et al., 2012 | In-person | Self-administered with no supervision | Computer |
| Frenchay Activities Index (FAI) | Holbrook & Skilbeck, 1983 | Not provided | Self-administered with no supervision | Not provided |
| Functional Ability Index (n=3) | Not provided | Not provided | Not provided | Not provided |
| Functional Assessment of Cancer Therapy- Bladder Cancer (FACT-BL) | Chen et al., 2020 | Remote | Self-administered with no supervision | Computer |
| Functional Assessment of Verbal Reasoning and Executive Strategies (FAVRES) | MacDonald, 2010 | In-person | Administered by an examiner/researcher/clinician | Not provided |
| Functional Activities Questionnaire (FAQ) (n=5) | Pfeffer et al., 1982 | In-person | Administered by an examiner/researcher/clinician | Not provided |
| Functional Cognitive Evaluation (FCE) | Not provided | In-person | Administered by an examiner/researcher/clinician | Not provided |
| Functional Composite Score | Not provided | In-person | Self-administered with no supervision | Not provided |
| Functional Independence Measure (FIM) | Granger et al., 1986 | Not provided | Not provided | Not provided |
| G-8 Questionnaire | Bellera et al., 2012 | In-person | Administered by an examiner/researcher/clinician | Not provided |
| GAITRite Assessment (n=2) | Bilney et al., 2003 | In-person | Administered by an examiner/researcher/clinician | Moving walkway |
| Gambling Task (Ojala et al., 2018) | Abdellaoui et al., 2008 | In-person | Self-administered under supervision | Computer |
| Gambling Task (Gomez-Andres et al., 2019) | Camara et al., 2010; Marco-Pallarés et al., 2008; Padrão et al., 2013 | In-person | Self-administered under supervision | Computer |
| Game of Dice Task (GDT) (n=13) | Brand et al., 2005 | Hybrid | Self-administered under supervision; Self-administered with no supervision | Computer |
| General Decision-Making Scale (GDMS) | Scott & Bruce, 1995 | Remote | Self-administered with no supervision | Computer |
| Geriatric Assessment (GA) | Klepin et al., 2011 | In-person | Administered by an examiner/researcher/clinician | Not provided |
| Goal Attainment Scaling (GAS) | Not provided | In-person | Administered by an examiner/researcher/clinician | Not provided |
| Go-NoGo Task | Luijten et al., 2011 | Remote | Self-administered with no supervision | Computer |
| Decision-making Competence Assessment Tool (DMCAT) (12-item Assessment) | Finucane & Gullion, 2010 | In-person | Administered by an examiner/researcher/clinician | Not provided |
| Hotel Task | Smith et al., 2022 | In-person | Administered by an examiner/researcher/clinician | Not provided |
| Hyogo Activities of Daily Living Scale (HADLS) | Nobutsugu et al., 1997 | In-person | Administered by an examiner/researcher/clinician | Not provided |
| Indecisiveness Scale-Frost and Shows (IS-FS) | Frost & Shows, 1993 | Remote | Self-administered with no supervision | Computer |
| Independent Living Scale (ILS) (n=3) | Loeb, 1996. | In-person | Administered by an examiner/researcher/clinician | Not provided |
| Index of Recognition Memory | Sobkow et al., 2020 | In-person | Self-administered with no supervision | Computer |
| Information Sampling Task | Clark et al., 2006 | In-person | Self-administered under supervision | Computer |
| Informed Consent (IC) | Moro et al., 2020 | In-person | Administered by an examiner/researcher/clinician | Paper/pen |
| Interleaved Pro- Anti-Saccade Task | Not provided | In-person | Self-administered under supervision | Computer |
| interRAI Home Care (HC) Assessment Tool (n=2) | Hirdes et al., 1999 | In-person | Administered by an examiner/researcher/clinician | Not provided |
| Intertemporal Choice Task (Geng et al., 2020) | Figner et al., 2010 | In-person | Self-administered under supervision | Computer |
| Intertemporal Choice Task (Li et al., 2021) | Toubia et al., 2013 | Remote | Self-administered with no supervision | Computer |
| Experiential Intertemporal Choice Task | Kwan et al., 2012; Palombo et al., 2015 | In-person | Self-administered under supervision | Computer |
| Hypothetical Intertemporal Choice Task | Kwan et al., 2012; Palombo et al., 2015 | In-person | Self-administered under supervision | Computer |
| Iowa Gambling Task (IGT) (n=74) | Bechara et al., 1994 | Hybrid | Self-administered with no supervision; Self-administered under supervision | Computer; fMRI |
| Item Response Task | Not provided | Remote | Self-administered with no supervision | Computer |
| Judgment Assessment Tool (JAT) | Escudier et al., 2016 | In-person | Administered by an examiner/researcher/clinician | Not provided |
| Karnofsky Performance Scale (KPS) | Karnofsky & Burchenal, 1949 | Not provided | Self-administered with no supervision | Paper/pen |
| Katz Index of Independence in Activities of Daily Living (ADL) Scale (n=6) | Katz et al., 1963 | Hybrid | Administered by an examiner/researcher/clinician | Not provided |
| Legal Capacity for Property Law Transactions Assessment Scale (LCPLTAS) (n=4) | Giannouli et al., 2018 | In-person | Administered by an examiner/researcher/clinician | Paper/pen |
| Legal Capacity for Property Law Transactions Assessment Scale-Short Form (LCPLTAS-SF) (n=2) | Giannouli et al., 2018 | In-person | Administered by an examiner/researcher/clinician | Paper/pen |
| Lexical Decision Task (LDT) | Not provided | In-person | Self-administered under supervision | Computer |
| Loss Aversion Task | Tom et al., 2007 | In-person | Self-administered under supervision | Computer |
| Lottery Task (Phan et al., 2023) | Not provided | In-person | Administered by an examiner/researcher/clinician | Not provided |
| Lottery Task (Sobkow et al., 2020) | Cokely & Kelley, 2009 | Remote | Self-administered with no supervision | Computer |
| Lottery Task (Sugden et al., 2019) | Not provided | In-person | Self-administered under supervision | Computer |
| MacArthur Competence Assessment Tool for Clinical Research (MacCAT-CR) | Appelbaum & Grisso, 2001 | In-person | Administered by an examiner/researcher/clinician | Not provided |
| MacArthur Competence Assessment Tool for Treatment (MacCAT-T) (n=8) | Grisso et al., 1997 | In-person | Administered by an examiner/researcher/clinician | Not provided |
| Matching Pennies Task | Barraclough et al., 2004 | In-person | Self-administered under supervision | Computer |
| Maximization Scale | Nenkov et al., 2008 | Remote | Self-administered with no supervision | Computer |
| Medical Decision-making Task (MDMT) | Not provided | Remote | Self-administered with no supervision | Computer |
| Melbourne Decision Making Questionnaire (MDMQ) | Mann et al., 1997 | Remote | Self-administered with no supervision | Not provided |
| Minimum Data Set for Home Care (MDS-HC) | Morris et al., 1997 | In-person | Administered by an examiner/researcher/clinician | Not provided |
| Mobility Composite Score | Not provided | In-person | Self-administered with no supervision | Computer |
| Modified Rankin Scale (mRS) (n=2) | Not provided | In-person | Administered by an examiner/researcher/clinician | Not provided |
| Monetary Choice Questionnaire (MCQ) (n=3) | Kirby et al., 1999 | Hybrid | Self-administered with no supervision; Self-administered under supervision | Computer |
| Moral Reasoning Task | Koenigs et al., 2007 | In-person | Administered by an examiner/researcher/clinician | Computer |
| Motor Gambling Task | Not provided | Remote | Self-administered with no supervision | Smartphone; Tablet (App-based) |
| Multi-step Decision-making Task | Huys et al., 2012 | In-person | Not provided | Not provided |
| Neuropsychological Assessment Battery Daily Living Module (NAB-DLM) | Stern & White, 2003. | In-person | Administered by an examiner/researcher/clinician | Not provided |
| Numerical Activities of Daily Living–Financial (NADL-F) | Arcara et al., 2019 |  | Not provided | Not provided |
| Object Size Comparison Task | Panichello et al., 2017 | In-person | Administered by an examiner/researcher/clinician | Computer; fMRI |
| On-Road Driving Assessment (n=3) | Wadley et al., 2009 | In-person | Administered by an examiner/researcher/clinician | Vehicle |
| Outcome Assessment: Decision-making | Zhu et al., 2018 | In-person | Administered by an examiner/researcher/clinician | Not provided |
| Outcome-Specific Devaluation Task | Morris et al., 2015 | In-person | Self-administered under supervision | Computer |
| Parkinson's Disease Activities of Daily Living, Interference, and Dependence (PD-AID) | Deal et al. 2019 | Hybrid | Administered by an examiner/researcher/clinician; Self-administered with no supervision | Tablet |
| Parkinson's Disease Questionnaire-39 (PDQ-39) | Peto et al., 1998 | Hybrid | Self-administered with no supervision | Computer; Tablet |
| Perceptual and Value-Based Decision-Making Task (PVDM) | Not provided | In-person | Self-administered under supervision | Computer |
| Performance Assessment of Self-Care Skills- Home (PASS-H) | Holm & Rogers, 1989 | In-person | Administered by an examiner/researcher/clinician | Not provided |
| "Pig" Dice Game | Scarne, 1945; Meder et al., 2016 | In-person | Self-administered under supervision | Computer |
| Post-decision Evidence Integration Task | Not provided | Remote | Self-administered with no supervision | Computer |
| Prediction Question (Mukherjee et al., 2020) | Not provided | In-person | Self-administered under supervision | Computer |
| Price Search Task | Not provided | In-person | Self-administered under supervision | Computer |
| Probabilistic Gambling Task | Marco-Pallares et al., 2008 | In-person | Self-administered under supervision | Computer |
| Probabilistic Prediction Task | Kreis et al., 2020 | In-person | Self-administered under supervision | Computer |
| Probabilistic Reversal Learning (PRL) Task | Not provided | In-person | Self-administered under supervision | Computer |
| Probabilistic Reward Task (PRT) | Pizzagalli et al., 2005 | In-person | Self-administered under supervision | Computer |
| Problem Solving Decision Making (PSDM) Scale | Kraetschmer, 1994 | In-person | Administered by an examiner/researcher/clinician | Not provided |
| Process Analysis of Daily Activity for Dementia (PADA-D) (n=2) | Tabira et al., 2020 | In-person | Administered by an examiner/researcher/clinician | Not provided |
| Pro-social Decision-making Task | Stolz et al., 2021 | In-person | Self-administered under supervision | Computer |
| Prostate Cancer Decision-making Questionnaire | Shaverdian et al., 2018 | In-person | Administered by an examiner/researcher/clinician | Not provided |
| Punishment Learning Task | Not provided | In-person | Self-administered under supervision | Computer |
| Questionnaire for History of Mental Activity (QHMA) | Not provided | In-person | Administered by an examiner/researcher/clinician | Not provided |
| Random Dot Motion (RDM) Task | Banca et al., 2015 | In-person | Self-administered under supervision | Computer |
| Relapse Analogue Task (RAT) | McKee, 2009 | In-person | Self-administered under supervision | Not provided |
| Reversal Learning Task (RLT) | Fellows & Farah, 2003 | In-person | Self-administered with no supervision | Computer |
| Reward Learning Task | Not provided | In-person | Self-administered under supervision | Computer |
| Risk Choice Experiment Survey (25-items) | Carvalho & Silverman, 2017 | Remote | Self-administered with no supervision | Computer |
| Risk Discounting Task (RDISC) | Yu et al., 2017 | Remote | Self-administered with no supervision | Computer |
| Risk Tolerance Task | Not provided | In-person | Not provided | Not provided |
| Risk-Based Decision-making Task | Hagiwara et al., 2022 | In-person | Self-administered under supervision | Computer |
| Risky Decision Scenario Task | Not provided | Remote | Self-administered with no supervision | Computer |
| Risky Decision-Making Task | Lane et al., 2005 | In-person | Self-administered under supervision | Computer |
| Road Sign Test | Ball et al., 2002 | In-person | Self-administered with no supervision | Computer |
| Rookwood Driving Battery (RDB) | McKenna et al., 2007 | Not provided | Not provided | Not provided |
| Self-Assessment Questionnaire | Not provided | In-person | Not provided | Not provided |
| Self-Care Decisions Inventory | Not provided | Remote | Self-administered with no supervision | Computer |
| Self-Care Heart Failure Index (SCHFI) | Riegel et al., 2004 | In-person | Administered by an examiner/researcher/clinician | Not provided |
| Shopping-decisions Task | UK Money Advice Service, 2016 | In-person | Self-administered with no supervision | Not provided |
| Decision Outcomes Inventory (DOI) | Bruine de Bruin et al., 2007 | In-person | Self-administered with no supervision | Not provided |
| Short Physical Performance Battery (SPPB) | Guralnik et al., 1994 | In-person | Administered by an examiner/researcher/clinician | Not provided |
| Situations Task | Not provided | In-person | Self-administered under supervision | Computer |
| Space Adventure Task (SAT) | Huys et al., 2015 | In-person | Self-administered under supervision | Computer |
| Standard Gamble (SG) | Not provided | In-person | Administered by an examiner/researcher/clinician | Not provided |
| Stockings of Cambridge (SoC) | Robbins et al., 1998 | In-person | Self-administered under supervision | Computer |
| Strategic Management Simulation Assessment | Streufert, 1988 | In-person | Self-administered under supervision | Computer |
| Switch Time Task | Not provided | In-person | Self-administered under supervision | Computer |
| Temporal Discounting Task | Boyle et al., 2012 | In-person | Administered by an examiner/researcher/clinician | Not provided |
| Test of Practical Judgment (TOP-J) (n=3) | Rabin et al., 2007 | In-person | Administered by an examiner/researcher/clinician | Not provided |
| Texas Functional Living Scale (TFLS) | Cullum et al., 2009 | In-person | Administered by an examiner/researcher/clinician | Not provided |
| The Action Selection Test (AST) (n=2) | Vlakveld, 2011 | In-person | Administered by an examiner/researcher/clinician | Computer |
| The Activities of Daily Living (ADL) Profile | Bottari et al., 2020 | In-person | Administered by an examiner/researcher/clinician | Not provided |
| The Barthel Index for Activities of Daily Living (ADL) Scale (n=12) | Mahoney & Barthel, 1965 | In-person | Administered by an examiner/researcher/clinician; Self-administered with no supervision | Paper/pen |
| The Cups Task | Weller et al., 2007 | In-person | Self-administered under supervision | Computer |
| The Current Medical Decision-making Capacity Rating (CMDC) | Marson et al., 2002 | In-person | Self-administered under supervision | Not provided |
| The Decision Self-Efficacy Scale (DSE) (n=3) | O'Connor, 1995 | Hybrid | Administered by an examiner/researcher/clinician | Computer; Paper/pen |
| The Effort-Expenditure for Reward Task (EEfRT) (n=2) | Treadway et al., 2009 | In-person | Self-administered under supervision | Computer |
| The Functional Assessment of Cancer Therapy - General (FACT-G) | Cella et al., 1993 | Hybrid | Administered by an examiner/researcher/clinician | Telephone |
| The Functional Comorbidity Index (FCI) | Fortin et al., 2005 | In-person | Administered by an examiner/researcher/clinician | Not provided |
| The Functioning Assessment Short Test (FAST) | Moser et al., 2013; Rosa et al., 2007 | In-person | Administered by an examiner/researcher/clinician | Not provided |
| The Lawton Instrumental Activities of Daily Living (IADL) Scale (n=25) | Lawton & Brody, 1969 | Hybrid | Administered by an examiner/researcher/clinician; Self-administered with no supervision; Self-administered under supervision | Paper/pen |
| The Montreal Cognitive Assessment (MoCA) | Nasreddine et al., 2005 | In-person | Administered by an examiner/researcher/clinician | Not provided |
| The Numerical Activities of Daily Living- Financial (NADL-F) Short Version | Arcara et al., 2017 | In-person | Administered by an examiner/researcher/clinician | Not provided |
| The Outcome and Assessment Information Set (OASIS) | O’Connor & Davitt, 2012 | Not provided | Not provided | Not provided |
| The Past and Present Financial Capacity Form (PPFCF) | Wadley, et al., 2003 | In-person | Self-administered under supervision | Computer |
| Three Domains of Judgment Test (3DJT) | Not provided | In-person | Self-administered under supervision | Computer |
| Time Preference Elicitation Task (DEEP Time) | Toubia et al. 2013 | Remote | Self-administered with no supervision | Computer |
| Timed Instrumental Activities of Daily Living (TIADL) (n=4) | Owsley et al., 2001 | In-person | Administered by an examiner/researcher/clinician | Not provided |
| Tokyo Metropolitan Institute of Gerontology Index of Competence (TMIG-IC) (n=2) | Koyano et al., 1991 | Hybrid | Administered by an examiner/researcher/clinician; Self-administered with no supervision; Self-administered under supervision | Paper/pen |
| Trail-Making Test (TMT) | Reitan et al., 1958 | In-person | Self-administered under supervision | Paper/pen |
| Two-Alternative Forced Choice (2AFC) Task | Santoyo-Mora et al., 2022 | In-person | Self-administered under supervision | Computer |
| Two-Choice Gambling Task (TCGT) | Schuermann et al., 2012 | Remote | Self-administered with no supervision | Computer |
| Two-Stimulus Visual Oddball Task | Herrmann & Knight, 2001 | Hybrid | Self-administered under supervision; Self-administered with no supervision | Tablet |
| Ultimatum Game | Guth et al., 1982 | In-person | Self-administered under supervision | Computer |
| Unified Parkinson’s Disease Rating Scale (MDS-UPDRS) | Goetz et al., 2007 | Hybrid | Administered by an examiner/researcher/clinician; Self-administered with no supervision | Tablet |
| University of Alabama at Birmingham (UAB) Life Space Questionnaire | Baker et al., 2003 | In-person | Self-administered with no supervision | Not provided |
| Useful Field of View (UFOV) (n=2) | Edwards et al., 2005 | In-person | Self-administered with no supervision | Computer |
| Visual Analogue Preference Task | Not provided | In-person | Self-administered under supervision | Computer |
| Vividness of Decision Outcomes | Not provided | Remote | Self-administered with no supervision | Computer; fMRI |
| WAIS-IV Matrix Reasoning Test | Wechsler, 2008 | In-person | Administered by an examiner/researcher/clinician | Computer; fMRI |
| Walking Task | Oliveira et al., 2018 | In-person | Self-administered under supervision | Tablet; Walking Pad |
| Willingness-to-Wait (WTW) Task (Mukherjee et al., 2020) | Not provided | In-person | Self-administered under supervision | Computer |
| Wisconsin Card Sorting Test (WCST) (n=3) | Heaton et al., 1993 | In-person | Self-administered under supervision | Computer |

*Note. Some measures were not strictly relevant to the functional outcomes domain, however, they were included in articles that focused on functional outcomes (e.g., lab based tasks such as the Iowa Gambling Task (IGT), Game of Dice Task (GDT), and Balloon Analogue Risk Task (BART) were measures included in articles that focused on older-adult decision-making and their independent activities of daily living (IADLs).*

# Table S3. Characteristics of most frequent and most relevant functional outcomes measures.

| **Measure** | **Measure Frequency** | **Citing Articles** | **Original Citation** | **Clinical Groups** | **Age Range Covered** | **Psychometric Properties** | **Format** | **In-person vs. Remote** | **Examiner vs. Self-administered** |
| --- | --- | --- | --- | --- | --- | --- | --- | --- | --- |
| Lawton IADL | 25 (5.6%) | Bang et al., 2022; Burgio et al., 2022; Buys et al., 2021; de Vries et al., 2021; Dos Santos et al., 2021; Emmert et al., 2021; Gareri et al., 2020; Geng et al., 2020; James et al., 2018; Leo et al., 2019; Li et al., 2022; Li et al., 2022; Lotvonen et al., 2018; McCarthy et al., 2018; Moro et al., 2020; Munir et al., 2022; Novais et al., 2021; Oba et al., 2021; Passler et al., 2020; Steward et al., 2019; Tabira et al., 2022; Wyld et al., 2021; Yuan et al., 2022; Saragih et al., 2022; Shi et al., 2020 | Lawton & Brody, 1969 | MCI; AD; Various forms of dementia; Stroke; Various cancers (colon, breast, pancreatic, head and neck, prostate); Advanced chronic kidney disease | 45-102 | Cronbach’s alpha= 0.86  Inter-rater reliability r= 0.85  (Older cancer patients; McCarthy et al., 2018) | Semi-structured interview; Paper/pen | Hybrid | Administered by examiner  Self-administered no supervision  Self-administered under supervision |
| FAQ | 5 (1.1%) | Becker et al., 2021; Berezuk et al., 2018; Fowler et al., 2018; Nowrangi et al., 2022; Wadley et al., 2020 | Pfeffer et al., 1982 | MCI; AD | 45-85+ | Not reported | Semi-structured interview | In-person | Administered by examiner |
| Barthel ADL | 12 (2.7%) | Chu et al., 2022; Gareri et al., 2020; Hodgson et al., 2021; Lee et al., 2020; Leo et al., 2019; Li et al., 2022; Li et al., 2022; Munir et al., 2022; Saragih et al., 2022; Shi et al., 2020; Wyld et al., 2021; Yang et al., 2021 | Mahoney & Barthel, 1965 | MCI; AD; Dementia; Care home residents; Various cancers (colon, head and neck, breast); Stroke; Unspecified chronic conditions | 18-102 | Test-retest k coefficients= 0.63-1  (Older adults; Lee et al., 2020) | Semi-structured interview; Paper/pen | In-person | Administered by examiner  Self-administered no supervision |
| Katz ADL | 6 (1.3%) | Bang et al., 2022; de Vries et al., 2021; James et al., 2018; Moro et al., 2020; Novais et al., 2021; Tsuda et al., 2019 | Katz et al., 1963 | AD; Various cancers (head and neck cancer, colon); chronic kidney disease; Home medical care patients | 65-100 | Not reported | Semi-structured interview | Hybrid | Administered by examiner |
| TMIG-IC | 2 (0.4%) | Oba et al., 2021; Yamashita et al., 2020 | Koyano et al., 1991 | AD; Patients hospitalized with heart failure | 64-94 | Not reported | Semi-structured interview; Paper/pen | Hybrid | Administered by an examiner/ researcher/clinician  Self-administered with no supervision  Self-administered under supervision |
| A-IADL | 1 (0.2%) | Verrijp et al., 2022 | Sikkes et al., 2012 | N/A | 18-97 | Not reported | Computer | Remote | Self-administered no supervision |
| TOP-J | 3 (0.7%) | Ord et al., 2019; Quinn et al., 2018; Rabin et al., 2022 | Rabin et al., 2007 | Subjective cognitive decline; MCI; Vascular dementia (VaD); Frontotemporal dementia; Primary progressive aphasia; patients referred for neuropsychological assessments; Older adult rehabilitation inpatients | 45-95 | Cronbach’s alpha= 0.95 (Informants and patients w/ MCI and various dementias; Rabin et al, 2021)  Cronbach’s alpha= 0.63  Inter-rater reliability k= 0.95  Test-retest r=0.78 (Older adults; Ord et al., 2021) | Semi-structured interview | In-person | Administered by examiner |
| ACED/SPACED | 2 (0.4%) | Fenton et al., 2023; Zilbershlag & Josman, 2019 | Lai et al., 2008 | MCI | 60-92 | Not reported | Semi-structured interview | In-person | Administered by examiner |
| EDMC | 1 (0.2%) | Pikouli et al., 2023 | Rosi et al. 2019 | MCI | 62-80 | Not reported | Computer | Remote | Self-administered under supervision |
| TIADL | 4 (0.9%) | Steward et al., 2019; Steward et al., 2019; Wadley et al., 2020; | Owsley et al., 2001 | MCI-ASD; MCI-AMD; MCI; AD | 45-85+ | Test-retest r= 0.85 (Older adults; Wadley et al., 2020) | Semi-structured interview; Performance-based task | In-person | Administered by examiner |
| ILS | 3 (0.7%) | Emmert et al., 2021; Lande et al., 2022; Muslemani et al., 2022; | Loeb, 1996 | Various dementias; Myotonic Dystrophy Type 1 (DM1) | 23-102 | Inter-rater reliability k=0.99  Test-retest r=0.80  (Muslemani et al., 2022) | Semi-structured interview; Performance-based task | In-person | Administered by examiner |
| PADA-D | 2 (0.4%) | Ikeda et al., 2019; Tabira et al., 2022 | Tabira, 2019 | AD | 45-85+ | Cronbach’s alpha= 0.96 (Patients with AD; Tabira et al., 2022) | Semi-structured interview | In-person | Administered by examiner |
| Texas Functional Living Scale | 1 (0.2%) | Ord et al., 2019 | Cullom et al., 2009 | Patients referred for neuropsychological assessments | 56-95 | Not reported | Semi-structured interview;Performance-based task | In-person | Administered by examiner |
| PASS-H | 1 (0.2%) | Zilbershlag & Josman, 2019 | Holm & Rogers, 2008 | N/A | 65-92 | Cronbach’s alpha subscale range=0.80-0.99  Test-retest subscale range r=0.82-0.97  (Zilbershlag & Josman, 2019) | Semi-structured interview;Performance-based task | In-person | Administered by examiner |

*Note. (1) Measure frequency, shown in the third column, indicates how often a measure was mentioned among the 450 total measures identified in the scoping review. (2) Psychometric properties were not reported for most measures. While certain measures appeared across multiple articles, very few articles mentioned internal consistency, test-retest reliability, and inter-rater reliability. (3) See Table S2 for full measure name.*

**Table S4.** Preferred Reporting Items for Systematic reviews and Meta-Analyses extension for Scoping Reviews (PRISMA-ScR) Checklist which indicates where each PRISMA-ScR item can be found in the manuscript and published scoping review protocol (Ho et al., 2024).

| **SECTION** | **ITEM** | **PRISMA-ScR CHECKLIST ITEM** | **REPORTED ON PAGE #** |
| --- | --- | --- | --- |
| **TITLE** | | | |
| Title | 1 | Identify the report as a scoping review. | 1 |
| **ABSTRACT** | | | |
| Structured summary | 2 | Provide a structured summary that includes (as applicable): background, objectives, eligibility criteria, sources of evidence, charting methods, results, and conclusions that relate to the review questions and objectives. | 2 |
| **INTRODUCTION** | | | |
| Rationale | 3 | Describe the rationale for the review in the context of what is already known. Explain why the review questions/objectives lend themselves to a scoping review approach. | 2 and 3 |
| Objectives | 4 | Provide an explicit statement of the questions and objectives being addressed with reference to their key elements (e.g., population or participants, concepts, and context) or other relevant key elements used to conceptualize the review questions and/or objectives. | 3 |
| **METHODS** | | | |
| Protocol and registration | 5 | Indicate whether a review protocol exists; state if and where it can be accessed (e.g., a Web address); and if available, provide registration information, including the registration number. | 3 |
| Eligibility criteria | 6 | Specify characteristics of the sources of evidence used as eligibility criteria (e.g., years considered, language, and publication status), and provide a rationale. | 4 and Table 1 |
| Information sources* | 7 | Describe all information sources in the search (e.g., databases with dates of coverage and contact with authors to identify additional sources), as well as the date the most recent search was executed. | 4 and Ho et al.. 2024 (see references) |
| Search | 8 | Present the full electronic search strategy for at least 1 database, including any limits used, such that it could be repeated. | Ho et al., 2024 (see references) |
| Selection of sources of evidence† | 9 | State the process for selecting sources of evidence (i.e., screening and eligibility) included in the scoping review. | 4 and 5 |
| Data charting process‡ | 10 | Describe the methods of charting data from the included sources of evidence (e.g., calibrated forms or forms that have been tested by the team before their use, and whether data charting was done independently or in duplicate) and any processes for obtaining and confirming data from investigators. | 5 |
| Data items | 11 | List and define all variables for which data were sought and any assumptions and simplifications made. | 5 |
| Critical appraisal of individual sources of evidence§ | 12 | If done, provide a rationale for conducting a critical appraisal of included sources of evidence; describe the methods used and how this information was used in any data synthesis (if appropriate). | N/A |
| Synthesis of results | 13 | Describe the methods of handling and summarizing the data that were charted. | 5 |
| **RESULTS** | | | |
| Selection of sources of evidence | 14 | Give numbers of sources of evidence screened, assessed for eligibility, and included in the review, with reasons for exclusions at each stage, ideally using a flow diagram. | 5 |
| Characteristics of sources of evidence | 15 | For each source of evidence, present characteristics for which data were charted and provide the citations. | 6 and 7 |
| Critical appraisal within sources of evidence | 16 | If done, present data on critical appraisal of included sources of evidence (see item 12). | N/A |
| Results of individual sources of evidence | 17 | For each included source of evidence, present the relevant data that were charted that relate to the review questions and objectives. | 6 and 7 |
| Synthesis of results | 18 | Summarize and/or present the charting results as they relate to the review questions and objectives. | 6 and 7 |
| **DISCUSSION** | | | |
| Summary of evidence | 19 | Summarize the main results (including an overview of concepts, themes, and types of evidence available), link to the review questions and objectives, and consider the relevance to key groups. | 7 and 8 |
| Limitations | 20 | Discuss the limitations of the scoping review process. | 8 and 9 |
| Conclusions | 21 | Provide a general interpretation of the results with respect to the review questions and objectives, as well as potential implications and/or next steps. | 8 and 9 |
| **FUNDING** | | | |
| Funding | 22 | Describe sources of funding for the included sources of evidence, as well as sources of funding for the scoping review. Describe the role of the funders of the scoping review. | 10 |

JBI = Joanna Briggs Institute; PRISMA-ScR = Preferred Reporting Items for Systematic reviews and Meta-Analyses extension for Scoping Reviews.

* Where *sources of evidence* (see second footnote) are compiled from, such as bibliographic databases, social media platforms, and Web sites.

† A more inclusive/heterogeneous term used to account for the different types of evidence or data sources (e.g., quantitative and/or qualitative research, expert opinion, and policy documents) that may be eligible in a scoping review as opposed to only studies. This is not to be confused with *information sources* (see first footnote).

‡ The frameworks by Arksey and O’Malley (6) and Levac and colleagues (7) and the JBI guidance (4, 5) refer to the process of data extraction in a scoping review as data charting*.*

§ The process of systematically examining research evidence to assess its validity, results, and relevance before using it to inform a decision. This term is used for items 12 and 19 instead of "risk of bias" (which is more applicable to systematic reviews of interventions) to include and acknowledge the various sources of evidence that may be used in a scoping review (e.g., quantitative and/or qualitative research, expert opinion, and policy document).

*PRISMA-ScR Checklist:* Tricco AC, Lillie E, Zarin W, O'Brien KK, Colquhoun H, Levac D, et al. PRISMA Extension for Scoping Reviews (PRISMAScR): Checklist and Explanation. Ann Intern Med. 2018;169:467–473. [doi: 10.7326/M18-0850](http://annals.org/aim/fullarticle/2700389/prisma-extension-scoping-reviews-prisma-scr-checklist-explanation).

*Protocol:* Ho, E. H., Ece, B., Novack, M. A., Pila, S., Karpouzian-Rogers, T., Mather, M. A., et al. (2024). Protocol for a multi-domain scoping review to identify measures of decision-making ability in an ageing population. *BMJ Open* 14, e084178. doi: 10.1136/bmjopen-2024-084178

**References of Articles Included in Functional Outcomes Domain**

Ahmed, J., Ward, N., Otto, J., McMahill, A., and Miller, E. E. (2023). Identifying Measures of Emotional Intelligence and Dangerous Driving. *Transportation Research Record: Journal of the Transportation Research Board* 2678, 365–375. doi: [10.1177/03611981231179698](https://doi.org/10.1177/03611981231179698)

Aloi, M., Rania, M., De Filippis, R., and Segura-Garcia, C. (2020). Weight and age do not account for a worse executive functioning among BED-obese patients. *Eat Weight Disord* 25, 373–377. doi: [10.1007/s40519-018-0608-9](https://doi.org/10.1007/s40519-018-0608-9)

Atkinson‐Clement, C., Cavazzini, É., Zénon, A., Witjas, T., Fluchère, F., Azulay, J., et al. (2019). Effects of subthalamic nucleus stimulation and levodopa on decision‐making in Parkinson’s disease. *Movement Disorders* 34, 377–385. doi: [10.1002/mds.27625](https://doi.org/10.1002/mds.27625)

Attridge, N., Pickering, J., Inglis, M., Keogh, E., and Eccleston, C. (2019). People in pain make poorer decisions. *Pain*160, 1662–1669. doi: [10.1097/j.pain.0000000000001542](https://doi.org/10.1097/j.pain.0000000000001542)

Ayers, C. R., Davidson, E. J., Dozier, M. E., and Twamley, E. W. (2019). Cognitive Rehabilitation and Exposure/Sorting Therapy for Late-Life Hoarding: Effects on Neuropsychological Performance. *The Journals of Gerontology: Series B*75, 1193–1198. doi: [10.1093/geronb/gbz062](https://doi.org/10.1093/geronb/gbz062)

Baeza-Velasco, C., Guillaume, S., Olié, E., Alacreu-Crespo, A., Cazals, A., and Courtet, P. (2020). Decision-making in major depressive disorder: Subjective complaint, objective performance, and discrepancy between both. *J Affect Disord* 270, 102–107. doi: [10.1016/j.jad.2020.03.064](https://doi.org/10.1016/j.jad.2020.03.064)

Bakhtiari, R., Tomczak, M. V., Langor, S., Scanlon, J. E. M., Granley, A., and Singhal, A. (2020). Application of tablet-based cognitive tasks to predict unsafe drivers in older adults. *Transportation Research Interdisciplinary Perspectives* 4, 100105. doi: [10.1016/j.trip.2020.100105](https://doi.org/10.1016/j.trip.2020.100105)

Bang, H. J., Shim, H. J., Kim, G. R., Hwang, J. E., Bae, W. K., Chung, I. J., et al. (2022). Geriatric functional assessment for decision-making on adjuvant chemotherapy in older colon cancer patients. *Korean J Intern Med* 37, 660–672. doi: [10.3904/kjim.2021.324](https://doi.org/10.3904/kjim.2021.324)

Banks, J., Carvalho, L. S., and Perez-Arce, F. (2019). Education, Decision Making, and Economic Rationality. *The Review of Economics and Statistics* 101, 428–441. doi: [10.1162/rest_a_00785](https://doi.org/10.1162/rest_a_00785)

Barch, D. M., Culbreth, A. J., Ben Zeev, D., Campbell, A., Nepal, S., and Moran, E. K. (2023). Dissociation of Cognitive Effort–Based Decision Making and Its Associations With Symptoms, Cognition, and Everyday Life Function Across Schizophrenia, Bipolar Disorder, and Depression. *Biological Psychiatry* 94, 501–510. doi: [10.1016/j.biopsych.2023.04.007](https://doi.org/10.1016/j.biopsych.2023.04.007)

Becker, S., Boettinger, O., Sulzer, P., Hobert, M. A., Brockmann, K., Maetzler, W., et al. (2021). Everyday Function in Alzheimer’s and Parkinson’s Patients with Mild Cognitive Impairment. *JAD* 79, 197–209. doi: [10.3233/JAD-200256](https://doi.org/10.3233/JAD-200256)

Bedwell, S. A., Harrison, N., Fradley, S., and Brooks, M. (2023). The role of sibling aggression during childhood in decision-making during adulthood. *Curr Psychol* 43, 2264–2276. doi: [10.1007/s12144-023-04475-7](https://doi.org/10.1007/s12144-023-04475-7)

Berezuk, C., Ramirez, J., Black, S. E., Zakzanis, K. K., and for the Alzheimer’s Disease Neuroimaging Initiative (2018). Managing money matters: Managing finances is associated with functional independence in MCI. *Int J Geriat Psychiatry* 33, 517–522. doi: [10.1002/gps.4817](https://doi.org/10.1002/gps.4817)

Bernard-Arevalo, S.-P., Laforce, R. J., Khayat, O., Bouchard, V., Bruneau, M.-A., Brunelle, S., et al. (2023). Clinical Assessment of Judgment in Adults and the Elderly: Development and Validation of the Three Domains of Judgment Test—Clinical Version (3DJT-CV). *JCM* 12, 3740. doi: [10.3390/jcm12113740](https://doi.org/10.3390/jcm12113740)

Best, R., and Freund, A. M. (2018). Age, Loss Minimization, and the Role of Probability for Decision-Making. *Gerontology* 64, 475–484. doi: [10.1159/000487636](https://doi.org/10.1159/000487636)

Betz, M. E., Hill, L. L., Fowler, N. R., DiGuiseppi, C., Han, S. D., Johnson, R. L., et al. (2022). “Is it time to stop driving?”: A randomized clinical trial of an online decision aid for older drivers. *J American Geriatrics Society* 70, 1987–1996. doi: [10.1111/jgs.17791](https://doi.org/10.1111/jgs.17791)

Biernacki, K., Molokotos, E., Han, C., Dillon, D. G., Leventhal, A. M., and Janes, A. C. (2023). Enhanced decision-making in nicotine dependent individuals who abstain: A computational analysis using Hierarchical Drift Diffusion Modeling. *Drug and Alcohol Dependence* 250, 110890. doi: [10.1016/j.drugalcdep.2023.110890](https://doi.org/10.1016/j.drugalcdep.2023.110890)

Bjork, J. M., Keyser-Marcus, L., Vassileva, J., Ramey, T., Houghton, D. C., and Moeller, F. G. (2021). Social Information Processing in Substance Use Disorders: Insights From an Emotional Go-Nogo Task. *Front. Psychiatry* 12, 672488. doi: [10.3389/fpsyt.2021.672488](https://doi.org/10.3389/fpsyt.2021.672488)

Blume, M., Schmidt, R., and Hilbert, A. (2018). Executive Functioning in Obesity, Food Addiction, and Binge-Eating Disorder. *Nutrients* 11, 54. doi: [10.3390/nu11010054](https://doi.org/10.3390/nu11010054)

Boubekri, M., Lee, J., MacNaughton, P., Woo, M., Schuyler, L., Tinianov, B., et al. (2020). The Impact of Optimized Daylight and Views on the Sleep Duration and Cognitive Performance of Office Workers. *IJERPH* 17, 3219. doi: [10.3390/ijerph17093219](https://doi.org/10.3390/ijerph17093219)

Bowren, M. D., Croft, K. E., Reber, J., and Tranel, D. (2018). Choosing spouses and houses: Impaired congruence between preference and choice following damage to the ventromedial prefrontal cortex. *Neuropsychology* 32, 280–303. doi: [10.1037/neu0000421](https://doi.org/10.1037/neu0000421)

Brady, B., Eramudugolla, R., Wood, J. M., and Anstey, K. J. (2021). Association between decision-making under risk conditions and on-road driving safety among older drivers. *Neuropsychology* 35, 622–629. doi: [10.1037/neu0000754](https://doi.org/10.1037/neu0000754)

Brière, M., Tocanier, L., Allain, P., Le Gal, D., Allet, G., Gorwood, P., et al. (2019). Decision-Making Measured by the Iowa Gambling Task in Patients with Alcohol Use Disorders Choosing Harm Reduction versus Relapse Prevention Program. *Eur Addict Res* 25, 182–190. doi: [10.1159/000499709](https://doi.org/10.1159/000499709)

Bruce, J. M., Bruce, A. S., Lynch, S., Thelen, J., Lim, S.-L., Smith, J., et al. (2018). Probability discounting of treatment decisions in multiple sclerosis: associations with disease knowledge, neuropsychiatric status, and adherence. *Psychopharmacology* 235, 3303–3313. doi: [10.1007/s00213-018-5037-y](https://doi.org/10.1007/s00213-018-5037-y)

Burgdorf, J. G., and Amjad, H. (2022). Cognitive impairment among medicare home health patients: comparing available measures. *Home Health Care Services Quarterly* 41, 139–148. doi: [10.1080/01621424.2021.2009392](https://doi.org/10.1080/01621424.2021.2009392)

Burgio, F., Benavides-Varela, S., Toffano, R., Palmer, K., Meneghello, F., Arcara, G., et al. (2022). Predicting financial deficits from a standard neuropsychological assessment: preliminary evidence in mild cognitive impairment. *Neurol Sci* 43, 299–303. doi: [10.1007/s10072-021-05304-0](https://doi.org/10.1007/s10072-021-05304-0)

Buys, S., Gustafsson, L., Gullo, H., Grimley, R., Summers, M., and Campbell, A. (2021). Cognition in the first week after stroke: how does it relate to personal and instrumental activities of daily living at follow-up? *Brain Impairment* 23, 185–195. doi: [10.1017/BrImp.2021.3](https://doi.org/10.1017/BrImp.2021.3)

Caballero, J. A., Auclair Ouellet, N., Phillips, N. A., and Pell, M. D. (2022). Social decision-making in Parkinson’s disease. *Journal of Clinical and Experimental Neuropsychology* 44, 302–315. doi: [10.1080/13803395.2022.2112554](https://doi.org/10.1080/13803395.2022.2112554)

Case, N. F., and Brown, T. G. (2023). The effects of depressed mood and 0.05 % blood alcohol concentration on risky driving in males. *Transportation Research Part F: Traffic Psychology and Behaviour* 96, 171–184. doi: [10.1016/j.trf.2023.06.006](https://doi.org/10.1016/j.trf.2023.06.006)

Caudle, M. M., Spadoni, A. D., Schiehser, D. M., Simmons, A. N., and Bomyea, J. (2023). Neural activity and network analysis for understanding reasoning using the matrix reasoning task. *Cogn Process* 24, 585–594. doi: [10.1007/s10339-023-01152-2](https://doi.org/10.1007/s10339-023-01152-2)

Chang, Y., Chen, Y., Chen, Y., Hsu, S., Yang, F., Lee, C., et al. (2020). Functional connectivity in default mode network correlates with severity of hypoxemia in obstructive sleep apnea. *Brain and Behavior* 10, e01889. doi: [10.1002/brb3.1889](https://doi.org/10.1002/brb3.1889)

Chen, X., Rutledge, R. B., Brown, H. R., Dolan, R. J., Bestmann, S., and Galea, J. M. (2018). Age-dependent Pavlovian biases influence motor decision-making. *PLoS Comput Biol* 14, e1006304. doi: [10.1371/journal.pcbi.1006304](https://doi.org/10.1371/journal.pcbi.1006304)

Chiu, E.-C., Yip, P.-K., Woo, P., and Lin, Y.-T. (2019). Test-retest reliability and minimal detectable change of the Cognitive Abilities Screening Instrument in patients with dementia. *PLoS ONE* 14, e0216450. doi: [10.1371/journal.pone.0216450](https://doi.org/10.1371/journal.pone.0216450)

Chu, M., Zhang, Y., Chen, J., Chen, W., Hong, Z., Zhang, Y., et al. (2022). Efficacy of Intermittent Theta-Burst Stimulation and Transcranial Direct Current Stimulation in Treatment of Post-Stroke Cognitive Impairment. *J. Integr. Neurosci.* 21, 130. doi: [10.31083/j.jin2105130](https://doi.org/10.31083/j.jin2105130)

Colón-Semenza, C., Fulford, D., and Ellis, T. (2021). Effort-Based Decision-Making for Exercise in People with Parkinson’s Disease. *JPD* 11, 725–735. doi: [10.3233/JPD-202353](https://doi.org/10.3233/JPD-202353)

Compagne, C., Gabriel, D., Ferrero, L., Magnin, E., and Tannou, T. (2023). Tools for the Assessment of Risk-Taking Behavior in Older Adults with Mild Dementia: A Cross-Sectional Clinical Study. *Brain Sciences* 13, 967. doi: [10.3390/brainsci13060967](https://doi.org/10.3390/brainsci13060967)

Croft, J., Grisham, J. R., Perfors, A., and Hayes, B. K. (2022). Risking Everything in Obsessive–Compulsive Disorder: An Analogue Decision-Making Study. *J Psychopathol Behav Assess* 44, 364–375. doi: [10.1007/s10862-021-09901-3](https://doi.org/10.1007/s10862-021-09901-3)

Culbreth, A. J., Dershwitz, S. D., Barch, D. M., and Moran, E. K. (2023). Associations Between Cognitive and Physical Effort–Based Decision Making in People With Schizophrenia and Healthy Control Subjects. *Biological Psychiatry: Cognitive Neuroscience and Neuroimaging* 8, 695–702. doi: [10.1016/j.bpsc.2023.02.003](https://doi.org/10.1016/j.bpsc.2023.02.003)

Curley, A., Murphy, R., Fleming, S., and Kelly, B. D. (2019). Age, psychiatry admission status and linear mental capacity for treatment decisions. *International Journal of Law and Psychiatry* 66, 101469. doi: [10.1016/j.ijlp.2019.101469](https://doi.org/10.1016/j.ijlp.2019.101469)

Cuypers, M., Lamers, R. E. D., Cornel, E. B., Van De Poll-Franse, L. V., De Vries, M., and Kil, P. J. M. (2018). The impact of prostate cancer diagnosis and treatment decision-making on health-related quality of life before treatment onset. *Support Care Cancer* 26, 1297–1304. doi: [10.1007/s00520-017-3953-8](https://doi.org/10.1007/s00520-017-3953-8)

Dammann, P., Herten, A., Santos, A. N., Rauschenbach, L., Chen, B., Darkwah Oppong, M., et al. (2021). Multimodal outcome assessment after surgery for brainstem cavernous malformations. *Journal of Neurosurgery* 135, 401–409. doi: [10.3171/2020.6.JNS201823](https://doi.org/10.3171/2020.6.JNS201823)

Danesin, L., Giustiniani, A., Arcara, G., and Burgio, F. (2022). Financial Decision-Making in Neurological Patients. *Brain Sciences* 12, 529. doi: [10.3390/brainsci12050529](https://doi.org/10.3390/brainsci12050529)

De Souza, N. A. P., De Oliveira, F., De Carvalho, R. L. S., and Dourado, M. C. N. (2022). The Relationship Between Decision-making Capacity and the Domains of Awareness in Alzheimer Disease. *Alzheimer Disease & Associated Disorders* 36, 58–63. doi: [10.1097/WAD.0000000000000484](https://doi.org/10.1097/WAD.0000000000000484)

De Vries, J., Bras, L., Sidorenkov, G., Festen, S., Steenbakkers, R. J. H. M., Langendijk, J. A., et al. (2021). Association of Deficits Identified by Geriatric Assessment With Deterioration of Health-Related Quality of Life in Patients Treated for Head and Neck Cancer. *JAMA Otolaryngol Head Neck Surg* 147, 1089. doi: [10.1001/jamaoto.2021.2837](https://doi.org/10.1001/jamaoto.2021.2837)

Deal, L. S., Andrae, D. A., Myers, D. E., Johnson, N., Foster, B., and Evans, C. J. (2022). The Measurement Performance of the Parkinson’s Disease Activities of Daily Living, Interference, and Dependence Instrument. *Front. Neurol.* 13, 760174. doi: [10.3389/fneur.2022.760174](https://doi.org/10.3389/fneur.2022.760174)

Del Missier, F., Hansson, P., Parker, A. M., Bruine De Bruin, W., and Mäntylä, T. (2020). Decision-making competence in older adults: A rosy view from a longitudinal investigation. *Psychology and Aging* 35, 553–564. doi: [10.1037/pag0000443](https://doi.org/10.1037/pag0000443)

Dinesh, A., Rai, S., and Praharaj, S. K. (2022). Decision-making and impulsivity in patients with alcohol dependence syndrome with comorbid attention deficit hyperactivity disorder. *Clinical Psychologist* 26, 92–104. doi: [10.1080/13284207.2021.1985377](https://doi.org/10.1080/13284207.2021.1985377)

Dos Santos, M., Licaj, I., Bellera, C., Cany, L., Binarelli, G., Soubeyran, P., et al. (2021). Cognitive Impairment in Older Cancer Patients Treated with First-Line Chemotherapy. *Cancers* 13, 6171. doi: [10.3390/cancers13246171](https://doi.org/10.3390/cancers13246171)

Drinkwater, K. G., Dagnall, N., Denovan, A., Parker, A., and Escolà-Gascón, Á. (2022). Paranormal Experience Profiles and Their Association With Variations in Executive Functions: A Latent Profile Analysis. *Front. Psychol.* 12, 778312. doi: [10.3389/fpsyg.2021.778312](https://doi.org/10.3389/fpsyg.2021.778312)

El Haj, M., and Moustafa, A. A. (2023). “Ten dollars today or 50 dollars after one month?” Temporal discounting in Korsakoff syndrome. *Cognitive Neuropsychiatry* 28, 116–129. doi: [10.1080/13546805.2023.2173059](https://doi.org/10.1080/13546805.2023.2173059)

Elshout, J. A., Bergsma, D. P., Sibbel, J., Baars-Elsinga, A., Lubbers, P., Van Asten, F., et al. (2018). Improvement in activities of daily living after visual training in patients with homonymous visual field defects using Goal Attainment Scaling. *Restorative Neurology and Neuroscience* 36, 1–12. doi: [10.3233/RNN-170719](https://doi.org/10.3233/RNN-170719)

Emmert, N. A., Schwarz, L. R., Vander Wal, J. S., and Gfeller, J. D. (2021). Neuropsychological predictors of health and safety abilities in dementia. *Applied Neuropsychology: Adult* 28, 94–106. doi: [10.1080/23279095.2019.1599893](https://doi.org/10.1080/23279095.2019.1599893)

Faulkner, P., Huys, Q. J. M., Renz, D., Eshel, N., Pilling, S., Dayan, P., et al. (2021). A comparison of ‘pruning’ during multi-step planning in depressed and healthy individuals. *Psychol. Med.* 52, 3948–3956. doi: [10.1017/S0033291721000799](https://doi.org/10.1017/S0033291721000799)

Feinstein, A., Meza, C., Stefan, C., and Staines, W. R. (2021). Impaired awareness: Why people with multiple sclerosis continue using cannabis despite evidence to the contrary. *Brain and Behavior* 11, e2220. doi: [10.1002/brb3.2220](https://doi.org/10.1002/brb3.2220)

Fenton, L., Han, S. D., DiGuiseppi, C. G., Fowler, N. R., Hill, L., Johnson, R. L., et al. (2023). Mild Cognitive Impairment is Associated with Poorer Everyday Decision Making. *JAD* 94, 1607–1615. doi: [10.3233/JAD-230222](https://doi.org/10.3233/JAD-230222)

Fernández Da Lama, R. G., and Brenlla, M. E. (2023). Time Perspective and Decision-Making: A Study on Decisional Patterns in Argentinian Adults. *Av. Psicol. Latinoam.* 41. doi: [10.12804/revistas.urosario.edu.co/apl/a.12046](https://doi.org/10.12804/revistas.urosario.edu.co/apl/a.12046)

Fernández, R. S., Crivelli, L., Pedreira, M. E., Allegri, R. F., and Correale, J. (2021). Computational basis of decision-making impairment in multiple sclerosis. *Mult Scler* 28, 1267–1276. doi: [10.1177/13524585211059308](https://doi.org/10.1177/13524585211059308)

Flegar, L., Baunacke, M., Buerk, B. T., Proschmann, R., Zacharis, A., Propping, S., et al. (2021). Decision Regret and Quality of Life after Focal Therapy with Vascular-Targeted Photodynamic Therapy (TOOKAD®) for Localized Prostate Cancer. *Urol Int* 106, 903–908. doi: [10.1159/000520084](https://doi.org/10.1159/000520084)

Fowler, M. E., Marotta, D. A., Kennedy, R. E., Gerstenecker, A., Gammon, M., and Triebel, K. (2021). Reliability of self‐report versus the capacity to consent to treatment instrument to make medical decisions in brain metastasis and other metastatic cancers. *Brain and Behavior* 11, e2303. doi: [10.1002/brb3.2303](https://doi.org/10.1002/brb3.2303)

Fowler, N., Shaaban, C. E., M. Torke, A., A. Lane, K., Saba, S., and E. Barnato, A. (2018). Im Not Sure We Had A Choice?: Decision Quality and The Use of Cardiac Implantable Electronic Devices In Older Adults With Cognitive Impairment. *Cardiol Cardiovascmed* 02, 10–26. doi: [10.26502/fccm.92920032](https://doi.org/10.26502/fccm.92920032)

Fujiyama, H., Tan, J., Puri, R., and Hinder, M. R. (2022). Influence of tDCS over right inferior frontal gyrus and pre-supplementary motor area on perceptual decision-making and response inhibition: A healthy ageing perspective. *Neurobiology of Aging* 109, 11–21. doi: [10.1016/j.neurobiolaging.2021.09.014](https://doi.org/10.1016/j.neurobiolaging.2021.09.014)

Galandra, C., Crespi, C., Basso, G., Manera, M. R., Giorgi, I., Poggi, P., et al. (2021). Decreased information processing speed and decision-making performance in alcohol use disorder: combined neurostructural evidence from VBM and TBSS. *Brain Imaging and Behavior* 15, 205–215. doi: [10.1007/s11682-019-00248-8](https://doi.org/10.1007/s11682-019-00248-8)

Gareri, P., Cotroneo, A. M., Orsitto, G., and Putignano, S. (2020). The CITIMEM study: A pilot study. Optimizing pharmacological treatment in dementia. *Archives of Gerontology and Geriatrics* 89, 104073. doi: [10.1016/j.archger.2020.104073](https://doi.org/10.1016/j.archger.2020.104073)

Gaubert, F., Borg, C., and Chainay, H. (2022). Decision-Making in Alzheimer’s Disease: The Role of Working Memory and Executive Functions in the Iowa Gambling Task and in Tasks Inspired by Everyday Situations. *JAD* 90, 1793–1815. doi: [10.3233/JAD-220581](https://doi.org/10.3233/JAD-220581)

Gaubert, F., Borg, C., Roux, J.-C., and Chainay, H. (2023). Decision-making and ageing: everyday life situations under risk and under ambiguity. *Quarterly Journal of Experimental Psychology* 77, 747–766. doi: [10.1177/17470218231182403](https://doi.org/10.1177/17470218231182403)

Geng, Z., Wu, X., Wang, L., Zhou, S., Tian, Y., Wang, K., et al. (2020). Reduced delayed reward selection by Alzheimer’s disease and mild cognitive impairment patients during intertemporal decision-making. *Journal of Clinical and Experimental Neuropsychology* 42, 298–306. doi: [10.1080/13803395.2020.1711873](https://doi.org/10.1080/13803395.2020.1711873)

Gerrans, P., Asher, A., and Earl, J. K. (2021). Cognitive functioning, financial literacy, and judgment in older age. *Accounting & Finance* 62, 1637–1674. doi: [10.1111/acfi.12835](https://doi.org/10.1111/acfi.12835)

Gerstenecker, A., Kennedy, R., Zhang, Y., Martin, R. C., Mackin, R. S., Weiner, M. W., et al. (2023). Item Response Analysis of the Financial Capacity Instrument-Short Form. *Archives of Clinical Neuropsychology* 38, 739–758. doi: [10.1093/arclin/acac112](https://doi.org/10.1093/arclin/acac112)

Giannouli, V., and Tsolaki, M. (2021). Vascular Dementia, Depression, and Financial Capacity Assessment. *Alzheimer Dis Assoc Disord* 35, 84–87. doi: [10.1097/WAD.0000000000000374](https://doi.org/10.1097/WAD.0000000000000374)

Giannouli, V., and Tsolaki, M. (2022a). Is negative affect associated with deficits in financial capacity in nondepressed older adults? A preliminary study. *Journal of Affective Disorders Reports* 10, 100391. doi: [10.1016/j.jadr.2022.100391](https://doi.org/10.1016/j.jadr.2022.100391)

Giannouli, V., and Tsolaki, M. (2022b). Liberating older adults from the bonds of vascular risk factors: What is their impact on financial capacity in amnestic mild cognitive impairment? *Psychiatry Clin Neurosci* 76, 246–250. doi: [10.1111/pcn.13348](https://doi.org/10.1111/pcn.13348)

Giannouli, V., and Tsolaki, M. (2022c). What Biological Factors, Social Determinants, and Psychological and Behavioral Symptoms of Patients with Mild Alzheimer’s Disease Correlate with Caregiver Estimations of Financial Capacity? Bringing Biases Against Older Women Into Focus. *Journal of Alzheimer’s Disease Reports* 6, 503–507. doi: [10.3233/ADR-220037](https://doi.org/10.3233/ADR-220037)

Giannouli, V., and Tsolaki, M. (2023). In the Hands of Hypnos: Associations between Sleep, Cognitive Performance and Financial Capacity in aMCI and Mild AD. *Sleep Sci* 16, 231–236. doi: [10.1055/s-0043-1770796](https://doi.org/10.1055/s-0043-1770796)

Giustiniani, J., Joucla, C., Bennabi, D., Nicolier, M., Chabin, T., Masse, C., et al. (2019). Behavioral and Electrophysiological Arguments in Favor of a Relationship between Impulsivity, Risk-Taking, and Success on the Iowa Gambling Task. *Brain Sciences* 9, 248. doi: [10.3390/brainsci9100248](https://doi.org/10.3390/brainsci9100248)

Gomez-Andres, A., Suades, A., Cucurell, D., De Miquel, M. A., Juncadella, M., and Rodríguez-Fornells, A. (2019). Electrophysiological correlates of feedback processing in subarachnoid hemorrhage patients. *NeuroImage: Clinical*24, 102075. doi: [10.1016/j.nicl.2019.102075](https://doi.org/10.1016/j.nicl.2019.102075)

Gowey, M. A., Neumeier, W. H., Henry, S., Wadley, V. G., Phillips, J., Hayden, K. M., et al. (2020). Executive function in individuals with clinically significant weight loss via behavioral intervention. *Obesity Science &amp; Practice* 7, 25–34. doi: [10.1002/osp4.458](https://doi.org/10.1002/osp4.458)

Grassi, G., Figee, M., Ooms, P., Righi, L., Nakamae, T., Pallanti, S., et al. (2018). Impulsivity and decision-making in obsessive-compulsive disorder after effective deep brain stimulation or treatment as usual. *CNS Spectr.* 23, 333–339. doi: [10.1017/S1092852918000846](https://doi.org/10.1017/S1092852918000846)

Grassi, G., Makris, N., and Pallanti, S. (2020). Addicted to compulsion: assessing three core dimensions of addiction across obsessive-compulsive disorder and gambling disorder. *CNS Spectr.* 25, 392–401. doi: [10.1017/S1092852919000993](https://doi.org/10.1017/S1092852919000993)

Gregório, M., Teixeira, A., Páscoa, R., Baptista, S., Carvalho, R., and Martins, C. (2020). The Problem-Solving Decision-Making scale-translation and validation for the Portuguese language: a cross-sectional study. *BMJ Open* 10, e033625. doi: [10.1136/bmjopen-2019-033625](https://doi.org/10.1136/bmjopen-2019-033625)

Gu, Y., Kociolek, A., Fernandez, K. K., Cosentino, S. A., Zhu, C. W., Jin, Z., et al. (2022). Clinical Trajectories at the End of Life in Autopsy-Confirmed Dementia Patients With Alzheimer Disease and Lewy Bodies Pathologies. *Neurology*98. doi: [10.1212/WNL.0000000000200259](https://doi.org/10.1212/WNL.0000000000200259)

Güngör, B., Budak, E., Taymur, I., Zorlu, N., Ucgun, B., Akgul, A., et al. (2018). The comparison of risky and ambiguity decision making and cool executive functions between patients with obsessive compulsive disorder and healthy controls. *Arch. Clin. Psychiatry (São Paulo)* 45, 112–118. doi: [10.1590/0101-60830000000170](https://doi.org/10.1590/0101-60830000000170)

Hall, P. A., Meng, G., Hudson, A., Sakib, M. N., Hitchman, S. C., MacKillop, J., et al. (2022). Cognitive function following SARS-CoV-2 infection in a population-representative Canadian sample. *Brain, Behavior, & Immunity - Health* 21, 100454. doi: [10.1016/j.bbih.2022.100454](https://doi.org/10.1016/j.bbih.2022.100454)

Hassani, D., Koelper, N., Borodyanskaya, Y., Arya, N. G., Rao, H., and Andy, U. (2022). Cognitive function following surgery for pelvic organ prolapse. *Neurourology and Urodynamics* 41, 1853–1861. doi: [10.1002/nau.25035](https://doi.org/10.1002/nau.25035)

Hayden, S. C. W., and Osborn, D. S. (2020). Impact of Worry on Career Thoughts, Career Decision State, and Cognitive Information Processing Skills. *Journal of Employment Couns* 57, 163–177. doi: [10.1002/joec.12152](https://doi.org/10.1002/joec.12152)

Hegedűs, K. M., Gál, B. I., Szkaliczki, A., Andó, B., Janka, Z., and Álmos, P. Z. (2021). Temperament, character and decision-making characteristics of patients with major depressive disorder following a suicide attempt. *PLoS ONE*16, e0251935. doi: [10.1371/journal.pone.0251935](https://doi.org/10.1371/journal.pone.0251935)

Heim, B., Peball, M., Saft, C., Von Hein, S. M., Ellmerer, P., Piater, J. M., et al. (2020). Time will tell: Decision making in premanifest and manifest Huntington’s disease. *Brain and Behavior* 10, e01843. doi: [10.1002/brb3.1843](https://doi.org/10.1002/brb3.1843)

Heintz, H. L., Paik, J. M., Baird, L., Driver, J. A., and Moye, J. (2023). What matters most to older adults: Racial and ethnic considerations in values for current healthcare planning. *J American Geriatrics Society* 71, 3254–3266. doi: [10.1111/jgs.18525](https://doi.org/10.1111/jgs.18525)

Hidrus, A., Kueh, Y. C., Norsa’adah, B., Chang, Y.-K., and Kuan, G. (2021). Effects of Brain Breaks Video Intervention of Decisional Balance among Malaysians with Type 2 Diabetes Mellitus: A Randomised Controlled Trial. *IJERPH*18, 8972. doi: [10.3390/ijerph18178972](https://doi.org/10.3390/ijerph18178972)

Ho, M.-C., Hsu, Y.-C., Lu, M.-L., Gossop, M., and Chen, V. C.-H. (2018). ‘Cool’ and ‘Hot’ executive functions in suicide attempters with major depressive disorder. *Journal of Affective Disorders* 235, 332–340. doi: [10.1016/j.jad.2018.04.057](https://doi.org/10.1016/j.jad.2018.04.057)

Hodgson, P., Greaves, J., Cook, G., Fraser, A., and Bainbridge, L. (2021). A study to introduce National Early Warning Scores (NEWS) in care homes: Influence on decision‐making and referral processes. *Nursing Open* 9, 519–526. doi: [10.1002/nop2.1091](https://doi.org/10.1002/nop2.1091)

Horne, K. S., Filmer, H. L., Nott, Z. E., Hawi, Z., Pugsley, K., Mattingley, J. B., et al. (2020). Evidence against benefits from cognitive training and transcranial direct current stimulation in healthy older adults. *Nat Hum Behav* 5, 146–158. doi: [10.1038/s41562-020-00979-5](https://doi.org/10.1038/s41562-020-00979-5)

Hou, Y., Chen, Y., Lai, S., Seery, S., Wang, L., Li, X., et al. (2023). Decision regret related to urinary diversion choices after cystectomy among Chinese bladder cancer patients. *Cancer Medicine* 12, 4786–4793. doi: [10.1002/cam4.5281](https://doi.org/10.1002/cam4.5281)

Huang, L.-C., and Yang, Y.-H. (2023). The Association Between Subjective Mental Impairment and Objective Cognitive Performance in Non-Demented, Very Mild and Mild Demented Individuals. *Am J Alzheimers Dis Other Demen* 38, 15333175231196061. doi: [10.1177/15333175231196061](https://doi.org/10.1177/15333175231196061)

Husain, S. F., Ong, S. K., Cuizhen, L., Tran, B., Ho, R. C., and Ho, C. S. (2021). Functional near-infrared spectroscopy during a decision-making task in patients with major depressive disorder. *Aust N Z J Psychiatry* 55, 485–493. doi: [10.1177/0004867420976856](https://doi.org/10.1177/0004867420976856)

Ikeda, Y., Ogawa, N., Yoshiura, K., Han, G., Maruta, M., Hotta, M., et al. (2019). Instrumental Activities of Daily Living: The Processes Involved in and Performance of These Activities by Japanese Community-Dwelling Older Adults with Subjective Memory Complaints. *IJERPH* 16, 2617. doi: [10.3390/ijerph16142617](https://doi.org/10.3390/ijerph16142617)

Imai, K., Masuda, M., Watanabe, H., Ogura, A., Ohdake, R., Tanaka, Y., et al. (2020). The neural network basis of altered decision‐making in patients with amyotrophic lateral sclerosis. *Ann Clin Transl Neurol* 7, 2115–2126. doi: [10.1002/acn3.51185](https://doi.org/10.1002/acn3.51185)

Irmen, F., Horn, A., Meder, D., Neumann, W., Plettig, P., Schneider, G., et al. (2019). Sensorimotor subthalamic stimulation restores risk‐reward trade‐off in Parkinson’s disease. *Movement Disorders* 34, 366–376. doi: [10.1002/mds.27576](https://doi.org/10.1002/mds.27576)

J. Zakrzewski, J., A. Gillett, D., R. Vigil, O., C. Smith, L., Komaiko, K., Chou, C.-Y., et al. (2020). Visually mediated functioning improves following treatment of hoarding disorder. *Journal of Affective Disorders* 264, 310–317. doi: [10.1016/j.jad.2019.12.030](https://doi.org/10.1016/j.jad.2019.12.030)

James, B. D., Wilson, R. S., Shah, R. C., Yu, L., Arvanitakis, Z., Bennett, D. A., et al. (2018). Association of Financial Literacy With Hospitalization in Community-dwelling Older Adults. *Medical Care* 56, 596–602. doi: [10.1097/MLR.0000000000000932](https://doi.org/10.1097/MLR.0000000000000932)

Jamieson, H. A., Abey-Nesbit, R., and Pickering, J. W. (2020). Effect of Capacity to Undertake Instrumental Activities of Daily Living on Entry to Aged Residential Care in Older People With Heart Failure. *Front. Med.* 7, 386. doi: [10.3389/fmed.2020.00386](https://doi.org/10.3389/fmed.2020.00386)

Jebraeili, H., Jafari, Z., and Feizi, S. (2021). Psychometric properties and factor structure of the Persian version of consideration of future consequences scale. *Advances in Cognitive Sciences* 23. doi: [10.30514/icss.23.3.14](https://doi.org/10.30514/icss.23.3.14)

Jehu, D. A., Davis, J. C., Barha, C. K., Vesely, K., Cheung, W., Ghag, C., et al. (2022). Sex Differences in Subsequent Falls and Falls Risk: A Prospective Cohort Study in Older Adults. *Gerontology* 68, 272–279. doi: [10.1159/000516260](https://doi.org/10.1159/000516260)

Karamaouna, P., Zouraraki, C., Economou, E., Kafetsios, K., Bitsios, P., and Giakoumaki, S. G. (2023). *Cold* executive function processes and their *hot* analogs in schizotypy. *J Int Neuropsychol Soc* 30, 285–294. doi: [10.1017/S1355617723000590](https://doi.org/10.1017/S1355617723000590)

Karyadi, K. A., Nitch, S. R., Kinney, D. I., and Britt, W. G. (2022). Decision making of forensic psychiatric inpatients deemed incompetent to stand trial. *Applied Neuropsychology: Adult* 29, 66–76. doi: [10.1080/23279095.2019.1709847](https://doi.org/10.1080/23279095.2019.1709847)

Kim, E. S., Suleman, S., and Hopper, T. (2020). Decision Making by People With Aphasia: A Comparison of Linguistic and Nonlinguistic Measures. *J Speech Lang Hear Res* 63, 1845–1860. doi: [10.1044/2020_JSLHR-19-00182](https://doi.org/10.1044/2020_JSLHR-19-00182)

Kolva, E., Rosenfeld, B., and Saracino, R. M. (2020). Neuropsychological Predictors of Decision-Making Capacity in Terminally Ill Patients with Advanced Cancer. *Archives of Clinical Neuropsychology* 35, 1–9. doi: [10.1093/arclin/acz027](https://doi.org/10.1093/arclin/acz027)

Kreis, I., Zhang, L., Moritz, S., and Pfuhl, G. (2022). Spared performance but increased uncertainty in schizophrenia: Evidence from a probabilistic decision-making task. *Schizophrenia Research* 243, 414–423. doi: [10.1016/j.schres.2021.06.038](https://doi.org/10.1016/j.schres.2021.06.038)

Lai, R.-Y., Desai, N. A., Amlang, C. J., Lin, C.-Y. R., Chen, T. X., Minyetty, M. J., et al. (2023). Gambling associated risk-taking decision in cerebellar ataxia. *Parkinsonism & Related Disorders* 107, 105252. doi: [10.1016/j.parkreldis.2022.105252](https://doi.org/10.1016/j.parkreldis.2022.105252)

Lande, E. S., Gauthier, J. R., and Stanley-Olson, A. (2022). Utilizing comprehensive neuropsychological assessment in adult protective service intervention. *Professional Psychology: Research and Practice* 53, 466–476. doi: [10.1037/pro0000403](https://doi.org/10.1037/pro0000403)

Lau, C. I., Chen, W., Wang, H., and Walsh, V. (2023). Decision‐making impairment under ambiguity but not under risk may underlie medication overuse in patients with chronic migraine. *Headache* 63, 822–833. doi: [10.1111/head.14513](https://doi.org/10.1111/head.14513)

Lebeau, J.-C., Mason, J., Roque, N., and Tenenbaum, G. (2022). The effects of acute exercise on driving and executive functions in healthy older adults. *International Journal of Sport and Exercise Psychology* 20, 283–301. doi: [10.1080/1612197X.2020.1849353](https://doi.org/10.1080/1612197X.2020.1849353)

Lee, N. G., Kang, T. W., and Park, H. J. (2020). Relationship Between Balance, Gait, and Activities of Daily Living in Older Adults With Dementia. *Geriatr Orthop Surg Rehabil* 11, 2151459320929578. doi: [10.1177/2151459320929578](https://doi.org/10.1177/2151459320929578)

Leo, S., Arnoldi, E., Repetto, L., Coccorullo, Z., Cinieri, S., Fedele, P., et al. (2019). Eribulin Mesylate as Third or Subsequent Line Chemotherapy for Elderly Patients with Locally Recurrent or Metastatic Breast Cancer: A Multicentric Observational Study of GIOGer (Italian Group of Geriatric Oncology)-ERIBE. *The Oncologist* 24, e232–e240. doi: [10.1634/theoncologist.2017-0676](https://doi.org/10.1634/theoncologist.2017-0676)

León, J. J., Fernández-Martin, P., González-Rodríguez, A., Rodríguez-Herrera, R., García-Pinteño, J., Pérez-Fernández, C., et al. (2023). Decision-making and frontoparietal resting-state functional connectivity among impulsive-compulsive diagnoses. Insights from a Bayesian approach. *Addictive Behaviors* 143, 107683. doi: [10.1016/j.addbeh.2023.107683](https://doi.org/10.1016/j.addbeh.2023.107683)

Li, M., Schulte, N., Elting, F., Winkler, E. C., Hetjens, S., Berger, A. K., et al. (2022a). Sequential Geriatric Assessment in Older Patients with Colorectal Cancer during Chemotherapy: Subgroup Analysis of a Prospective, Multicenter Study EpiReal 75. *Oncol Res Treat* 45, 670–680. doi: [10.1159/000525101](https://doi.org/10.1159/000525101)

Li, M., Schulte, N., Elting, F., Winkler, E. C., Hetjens, S., Berger, A. K., et al. (2022b). Sequential Geriatric Assessment in Older Patients with Colorectal Cancer during Chemotherapy: Subgroup Analysis of a Prospective, Multicenter Study EpiReal 75. *Oncol Res Treat* 45, 670–680. doi: [10.1159/000525101](https://doi.org/10.1159/000525101)

Li, Y., Krefeld-Schwalb, A., Wall, D. G., Johnson, E. J., Toubia, O., and Bartels, D. M. (2022c). The More You Ask, the Less You Get: When Additional Questions Hurt External Validity. *Journal of Marketing Research* 59, 963–982. doi: [10.1177/00222437211073581](https://doi.org/10.1177/00222437211073581)

Lichtenberg, P. A., Tocco, M., Campbell, R., and Shipp, M. (2021). Which Items of the Financial Decision Tracker Differentiate Those with Decision-making Deficits from Those with No Deficits? Data from the Michigan APS Implementation Project. *Clinical Gerontologist* 44, 577–584. doi: [10.1080/07317115.2021.1901167](https://doi.org/10.1080/07317115.2021.1901167)

Limbrick-Oldfield, E. H., Mick, I., Cocks, R. E., Flechais, R. S. A., Turton, S., Lingford-Hughes, A., et al. (2020). Neural and neurocognitive markers of vulnerability to gambling disorder: a study of unaffected siblings. *Neuropsychopharmacol.* 45, 292–300. doi: [10.1038/s41386-019-0534-1](https://doi.org/10.1038/s41386-019-0534-1)

Ljunggren, S., Winblad, S., Samuelsson, H., and Malmgren, K. (2023). Decision-making under ambiguity after frontal lobe resection for epilepsy. *Epilepsy & Behavior* 142, 109215. doi: [10.1016/j.yebeh.2023.109215](https://doi.org/10.1016/j.yebeh.2023.109215)

Lloyd, K., Gaunt, D., Haunton, V., Skelly, R., Mann, H., Ben-Shlomo, Y., et al. (2020). Driving in Parkinson’s disease: a retrospective study of driving and mobility assessments. *Age and Ageing* 49, 1097–1101. doi: [10.1093/ageing/afaa098](https://doi.org/10.1093/ageing/afaa098)

Logge, W. B., Morley, K. C., Haber, P. S., and Baillie, A. J. (2023). Impaired Decision-Making and Skin Conductance Responses Are Associated with Reward and Punishment Sensitivity in Individuals with Severe Alcohol Use Disorder. *Neuropsychobiology* 82, 117–129. doi: [10.1159/000529156](https://doi.org/10.1159/000529156)

Lohse, A., Løkkegaard, A., Siebner, H. R., and Meder, D. (2023). Linking Impulsivity to Activity Levels in Pre-Supplementary Motor Area during Sequential Gambling. *J. Neurosci.* 43, 1414–1421. doi: [10.1523/JNEUROSCI.1287-22.2023](https://doi.org/10.1523/JNEUROSCI.1287-22.2023)

Lotvonen, S., Kyngäs, H., Koistinen, P., Bloigu, R., and Elo, S. (2018). Mental Well-Being of Older People in Finland during the First Year in Senior Housing and Its Association with Physical Performance. *IJERPH* 15, 1331. doi: [10.3390/ijerph15071331](https://doi.org/10.3390/ijerph15071331)

Lu, S. (2023). Functional Ability and Its Associated Factors Among Chinese People Aged 50 Years and Above from the Perspective of Healthy Aging: A 7-Year Longitudinal Study. *Journal of Aging & Social Policy*, 1–16. doi: [10.1080/08959420.2023.2226286](https://doi.org/10.1080/08959420.2023.2226286)

Ma, X., Megli, A., Pittenger, C., and Pushkarskaya, H. (2021). OCD Influences Evidence Accumulation During Decision Making in Males but Not Females During Perceptual and Value-Driven Choice. *Front. Psychiatry* 12, 687680. doi: [10.3389/fpsyt.2021.687680](https://doi.org/10.3389/fpsyt.2021.687680)

Mallorquí-Bagué, N., Tolosa-Sola, I., Fernández-Aranda, F., Granero, R., Fagundo, A. B., Lozano-Madrid, M., et al. (2018). Cognitive Deficits in Executive Functions and Decision-Making Impairments Cluster Gambling Disorder Sub-types. *J Gambl Stud* 34, 209–223. doi: [10.1007/s10899-017-9724-0](https://doi.org/10.1007/s10899-017-9724-0)

Mao, T., Fang, Z., Chai, Y., Deng, Y., Rao, J., Quan, P., et al. (2023). Sleep deprivation attenuates neural responses to outcomes from risky decision‐making. *Psychophysiology* 61, e14465. doi: [10.1111/psyp.14465](https://doi.org/10.1111/psyp.14465)

Marazia, C., Rucci, P., Fangerau, H., Voßberg, D., Rolfes, V., Iozzino, L., et al. (2022). Treatment Decision-Making Capacity in Forensic vs Non-forensic Psychiatric Patients: A European Comparison. *Schizophrenia Bulletin Open* 3, sgac037. doi: [10.1093/schizbullopen/sgac037](https://doi.org/10.1093/schizbullopen/sgac037)

Marques, A., Pereira, B., Figorilli, M., Vidal, T., Deffarges, P., Durif, F., et al. (2022). Decision making under uncertainty in Parkinson’s disease with Rem sleep behavior disorder. *Sleep Medicine* 90, 214–221. doi: [10.1016/j.sleep.2022.01.025](https://doi.org/10.1016/j.sleep.2022.01.025)

Martin, R. C., Gerstenecker, A., Triebel, K. L., Falola, M., McPherson, T., Cutter, G., et al. (2019). Declining Financial Capacity in Mild Cognitive Impairment: A Six-Year Longitudinal Study. *Archives of Clinical Neuropsychology* 34, 152–161. doi: [10.1093/arclin/acy030](https://doi.org/10.1093/arclin/acy030)

Martini, A., Ellis, S. J., Grange, J. A., Tamburin, S., Dal Lago, D., Vianello, G., et al. (2018). Risky decision-making and affective features of impulse control disorders in Parkinson’s disease. *J Neural Transm* 125, 131–143. doi: [10.1007/s00702-017-1807-7](https://doi.org/10.1007/s00702-017-1807-7)

Marton, T., Samuels, J., Nestadt, P., Krasnow, J., Wang, Y., Shuler, M., et al. (2019). Validating a dimension of doubt in decision-making: A proposed endophenotype for obsessive-compulsive disorder. *PLoS ONE* 14, e0218182. doi: [10.1371/journal.pone.0218182](https://doi.org/10.1371/journal.pone.0218182)

Maurage, P., Lannoy, S., Dormal, V., Blanco, M., and Trabut, J. (2018). Clinical Usefulness of the Iowa Gambling Task in Severe Alcohol Use Disorders: Link with Relapse and Cognitive‐Physiological Deficits. *Alcoholism Clin &amp; Exp Res* 42, 2266–2273. doi: [10.1111/acer.13873](https://doi.org/10.1111/acer.13873)

McCarthy, A. L., Peel, N. M., Gillespie, K. M., Berry, R., Walpole, E., Yates, P., et al. (2018). Validation of a frailty index in older cancer patients with solid tumours. *BMC Cancer* 18, 892. doi: [10.1186/s12885-018-4807-6](https://doi.org/10.1186/s12885-018-4807-6)

McDermott, T. J., Berg, H., Touthang, J., Akeman, E., Cannon, M. J., Santiago, J., et al. (2022). Striatal reactivity during emotion and reward relates to approach–avoidance conflict behaviour and is altered in adults with anxiety or depression. *jpn* 47, E311–E322. doi: [10.1503/jpn.220083](https://doi.org/10.1503/jpn.220083)

McWilliams, E. C., Barbey, F. M., Dyer, J. F., Islam, M. N., McGuinness, B., Murphy, B., et al. (2021). Feasibility of Repeated Assessment of Cognitive Function in Older Adults Using a Wireless, Mobile, Dry-EEG Headset and Tablet-Based Games. *Front. Psychiatry* 12, 574482. doi: [10.3389/fpsyt.2021.574482](https://doi.org/10.3389/fpsyt.2021.574482)

Metz, M. J., Veerbeek, M. A., Van Der Feltz-Cornelis, C. M., De Beurs, E., and Beekman, A. T. F. (2018). Decisional conflict in mental health care: a cross-sectional study. *Soc Psychiatry Psychiatr Epidemiol* 53, 161–169. doi: [10.1007/s00127-017-1467-9](https://doi.org/10.1007/s00127-017-1467-9)

Millroth, P., Nilsson, H., and Juslin, P. (2019). The decision paradoxes motivating Prospect Theory: The prevalence of the paradoxes increases with numerical ability. *Judgm. decis. mak.* 14, 513–533. doi: [10.1017/S1930297500006161](https://doi.org/10.1017/S1930297500006161)

Mimmack, K. J., Sprague, E. H., Amariglio, R. E., Vannini, P., and Marshall, G. A. (2023). Longitudinal Evolution of Financial Capacity and Cerebral Tau and Amyloid Burden in Older Adults with Normal Cognition or Mild Cognitive Impairment. *The Journal of Prevention of Alzheimer’s Disease* 11, 966–974. doi: [10.14283/jpad.2023.121](https://doi.org/10.14283/jpad.2023.121)

Moccia, L., Quintigliano, M., Janiri, D., De Martin, V., Rogier, G., Sani, G., et al. (2021). Heart rate variability and interoceptive accuracy predict impaired decision-making in Gambling Disorder. *J Behav Addict* 10, 701–710. doi: [10.1556/2006.2021.00067](https://doi.org/10.1556/2006.2021.00067)

Moore, T. M., Di Sandro, A., Scott, J. C., Lopez, K. C., Ruparel, K., Njokweni, L. J., et al. (2023). Construction of a computerized adaptive test (CAT-CCNB) for efficient neurocognitive and clinical psychopathology assessment. *Journal of Neuroscience Methods* 386, 109795. doi: [10.1016/j.jneumeth.2023.109795](https://doi.org/10.1016/j.jneumeth.2023.109795)

Moran, E. K., Prevost, C., Culbreth, A. J., and Barch, D. M. (2023). Effort-cost decision-making in psychotic and mood disorders. *Journal of Psychopathology and Clinical Science* 132, 490–498. doi: [10.1037/abn0000822](https://doi.org/10.1037/abn0000822)

Moreira, H. S., Costa, A. S., Machado, Á., Castro, S. L., Vicente, S. G., and Lima, C. F. (2022). Impaired Recognition of Facial and Vocal Emotions in Mild Cognitive Impairment. *J Int Neuropsychol Soc* 28, 48–61. doi: [10.1017/S135561772100014X](https://doi.org/10.1017/S135561772100014X)

Moro, V., Valbusa, V., Corsi, N., Bonazzi, A., Condoleo, M. T., Broggio, E., et al. (2020). Comprehension of written texts for the assessment of clinical competence and decision making in people with mild to moderate Alzheimer disease. *Neurol Sci* 41, 1225–1231. doi: [10.1007/s10072-019-04228-0](https://doi.org/10.1007/s10072-019-04228-0)

Moses-Payne, M. E., Rollwage, M., Fleming, S. M., and Roiser, J. P. (2019). Postdecision Evidence Integration and Depressive Symptoms. *Front. Psychiatry* 10, 639. doi: [10.3389/fpsyt.2019.00639](https://doi.org/10.3389/fpsyt.2019.00639)

Moynihan, G., O’Reilly, K., O’Connor, J., and Kennedy, H. G. (2018). An evaluation of functional mental capacity in forensic mental health practice: the Dundrum capacity ladders validation study. *BMC Psychiatry* 18, 78. doi: [10.1186/s12888-018-1658-2](https://doi.org/10.1186/s12888-018-1658-2)

Mueller, S. M., Arias, M. G., Mejuto Vázquez, G., Schiebener, J., Brand, M., and Wegmann, E. (2019). Decision support in patients with mild Alzheimer’s disease. *Journal of Clinical and Experimental Neuropsychology* 41, 484–496. doi: [10.1080/13803395.2019.1585517](https://doi.org/10.1080/13803395.2019.1585517)

Mukherjee, D., Lee, S., Kazinka, R., D. Satterthwaite, T., and Kable, J. W. (2020). Multiple Facets of Value-Based Decision Making in Major Depressive Disorder. *Sci Rep* 10, 3415. doi: [10.1038/s41598-020-60230-z](https://doi.org/10.1038/s41598-020-60230-z)

Müller, S. M., Schiebener, J., Brand, M., and Liebherr, M. (2021). Decision-making, cognitive functions, impulsivity, and media multitasking expectancies in high versus low media multitaskers. *Cogn Process* 22, 593–607. doi: [10.1007/s10339-021-01029-2](https://doi.org/10.1007/s10339-021-01029-2)

Munguía, L., Lucas, I., Jiménez‐Murcia, S., Mora‐Maltas, B., Granero, R., Miranda‐Olivos, R., et al. (2021). Executive functions in binge spectrum eating disorders with comorbid compulsive buying. *Euro Eating Disorders Rev* 29, 854–867. doi: [10.1002/erv.2855](https://doi.org/10.1002/erv.2855)

Munir, A., Huws, A., Khan, S., Sharaiha, Y., Holt, S., and Khawaja, S. (2022). Geriatric assessment tool application in treatment recommendations for older women with breast cancer. *The Breast* 63, 101–107. doi: [10.1016/j.breast.2022.03.012](https://doi.org/10.1016/j.breast.2022.03.012)

Muslemani, S., Gagnon, C., and Gallais, B. (2022). Instrumental activities of daily living in adults with the DM1 childhood phenotype: going beyond motor impairments. *Neuromuscular Disorders* 32, 313–320. doi: [10.1016/j.nmd.2022.02.004](https://doi.org/10.1016/j.nmd.2022.02.004)

Nakamizo, A., Amano, T., Matsuo, S., Kuwashiro, T., Yasaka, M., and Okada, Y. (2020). Common carotid flow velocity is associated with cognitive function after carotid endarterectomy. *Journal of Clinical Neuroscience* 76, 53–57. doi: [10.1016/j.jocn.2020.04.050](https://doi.org/10.1016/j.jocn.2020.04.050)

Nakao, A., Yamanouchi, J., Takenaka, K., and Takada, K. (2020). The Iowa Gambling Task on HIV-infected subjects. *Journal of Infection and Chemotherapy* 26, 240–244. doi: [10.1016/j.jiac.2019.09.008](https://doi.org/10.1016/j.jiac.2019.09.008)

Newton, F. J., Haregu, T. N., Newton, J. D., Donovan, R., Mahal, A., Mackenzie-Stewart, R., et al. (2023). Effects of customer relationship management (CRM) strategies and socio-cognitive constructs on the physical activity of individuals with arthritis over time. *PLoS ONE* 18, e0292692. doi: [10.1371/journal.pone.0292692](https://doi.org/10.1371/journal.pone.0292692)

Nigro, S. E., Wu, M., C. Juliano, A., Flynn, B., Lu, L. H., Landay, A. L., et al. (2021). Effects of cocaine and HIV on decision-making abilities. *J. Neurovirol.* 27, 422–433. doi: [10.1007/s13365-021-00965-1](https://doi.org/10.1007/s13365-021-00965-1)

Noaman, S., Al-Mukhtar, O., Abramovic, S., Mohammed, H., Goh, C. Y., Long, C., et al. (2019). Changes in Statin Prescription Patterns in Patients Admitted to an Australian Geriatric Subacute Unit. *Heart, Lung and Circulation* 28, 423–429. doi: [10.1016/j.hlc.2017.12.009](https://doi.org/10.1016/j.hlc.2017.12.009)

Novais, T., Pongan, E., Gervais, F., Coste, M.-H., Morelon, E., Krolak-Salmon, P., et al. (2021). Pretransplant Comprehensive Geriatric Assessment in Older Patients with Advanced Chronic Kidney Disease. *Nephron* 145, 692–701. doi: [10.1159/000517342](https://doi.org/10.1159/000517342)

Nowrangi, M. A., Outen, J. D., Naaz, F., Chen, L., Bakker, A., Munro, C. A., et al. (2022). Altered Angular Gyrus Resting State Functional Connectivity Associated with Financial Capacity in Mild Cognitive Impairment. *JAD* 86, 763–771. doi: [10.3233/JAD-215148](https://doi.org/10.3233/JAD-215148)

Noyes, K., Zapf, A. L., Depner, R. M., Flores, T., Huston, A., Rashid, H. H., et al. (2022). Problem-solving skills training in adult cancer survivors: Bright IDEAS-AC pilot study. *Cancer Treatment and Research Communications* 31, 100552. doi: [10.1016/j.ctarc.2022.100552](https://doi.org/10.1016/j.ctarc.2022.100552)

Nurmi, E. L., Laughlin, C. P., De Wit, H., Palmer, A. A., MacKillop, J., Cannon, T. D., et al. (2023). Polygenic contributions to performance on the Balloon Analogue Risk Task. *Mol Psychiatry* 28, 3524–3530. doi: [10.1038/s41380-023-02123-x](https://doi.org/10.1038/s41380-023-02123-x)

Oba, H., Kadoya, Y., Okamoto, H., Matsuoka, T., Abe, Y., Shibata, K., et al. (2021). The Economic Burden of Dementia: Evidence from a Survey of Households of People with Dementia and Their Caregivers. *IJERPH* 18, 2717. doi: [10.3390/ijerph18052717](https://doi.org/10.3390/ijerph18052717)

Occhiogrosso, J., Hemminger, L., Burke, J., Ibegbu, C., Serventi, J., and Mohile, N. (2020). Treatment-Related Decisions in Malignant Gliomas: A Feasibility Study. *Journal of Palliative Medicine* 23, 264–267. doi: [10.1089/jpm.2019.0242](https://doi.org/10.1089/jpm.2019.0242)

Ojala, K. E., Janssen, L. K., Hashemi, M. M., Timmer, M. H. M., Geurts, D. E. M., Ter Huurne, N. P., et al. (2018). Dopaminergic Drug Effects on Probability Weighting during Risky Decision Making. *eNeuro* 5, ENEURO.0330-18.2018. doi: [10.1523/ENEURO.0330-18.2018](https://doi.org/10.1523/ENEURO.0330-18.2018)

Olié, E., Catanzaro, T., Malestroit, M., Guija, J. A., Giner, L., and Courtet, P. (2023). The capacity to consent to treatment is altered in suicidal patients. *Ann Gen Psychiatry* 22, 35. doi: [10.1186/s12991-023-00459-w](https://doi.org/10.1186/s12991-023-00459-w)

Oliveira, A. S., Reiche, M. S., Vinescu, C. I., Thisted, S. A. H., Hedberg, C., Castro, M. N., et al. (2018). The cognitive complexity of concurrent cognitive-motor tasks reveals age-related deficits in motor performance. *Sci Rep* 8, 6094. doi: [10.1038/s41598-018-24346-7](https://doi.org/10.1038/s41598-018-24346-7)

Ord, A. S., Phillips, J. I., Wolterstorff, T., Kintzing, R., Slogar, S.-M., and Sautter, S. W. (2019). Can deficits in functional capacity and practical judgment indicate cognitive impairment in older adults? *Applied Neuropsychology: Adult* 28, 737–744. doi: [10.1080/23279095.2019.1698582](https://doi.org/10.1080/23279095.2019.1698582)

Orduz-Bastidas, L., Martínez-Martínez, A., Hurtado-Parrado, C., López-López, W., and Acevedo-Triana, C. (2020). Parkinson disease patients; performance in Theory of Mind (ToM) and decision-making tasks with and without Deep Brain Stimulation (DBS). *Ter Psicol* 38, 259–282. doi: [10.4067/S0718-48082020000200259](https://doi.org/10.4067/S0718-48082020000200259)

Ostendorf, S., Müller, S. M., and Brand, M. (2020). Neglecting Long-Term Risks: Self-Disclosure on Social Media and Its Relation to Individual Decision-Making Tendencies and Problematic Social-Networks-Use. *Front. Psychol.* 11, 543388. doi: [10.3389/fpsyg.2020.543388](https://doi.org/10.3389/fpsyg.2020.543388)

Ouerchefani, R., Ouerchefani, N., Allain, P., Ben Rejeb, M. R., and Le Gall, D. (2019). Relationships between executive function, working memory, and decision‐making on the Iowa Gambling Task: Evidence from ventromedial patients, dorsolateral patients, and normal subjects. *Journal of Neuropsychology* 13, 432–461. doi: [10.1111/jnp.12156](https://doi.org/10.1111/jnp.12156)

Ouerchefani, R., Ouerchefani, N., Ben Rejeb, M. R., and Le Gall, D. (2022). Impaired Perception of Unintentional Transgression of Social Norms after Prefrontal Cortex Damage: Relationship to Decision Making, Emotion Recognition, and Executive Functions. *Archives of Clinical Neuropsychology* 37, 249–273. doi: [10.1093/arclin/acab078](https://doi.org/10.1093/arclin/acab078)

Ouerfelli-Ethier, J., Elsaeid, B., Desgroseilliers, J., Munoz, D. P., Blohm, G., and Khan, A. Z. (2018). Anti-saccades predict cognitive functions in older adults and patients with Parkinson’s disease. *PLoS ONE* 13, e0207589. doi: [10.1371/journal.pone.0207589](https://doi.org/10.1371/journal.pone.0207589)

Ozcan, S., Gica, S., and Gulec, H. (2020). Suicidal behavior in treatment resistant major depressive disorder patients treated with transmagnetic stimulation(TMS) and its relationship with cognitive functions. *Psychiatry Research* 286, 112873. doi: [10.1016/j.psychres.2020.112873](https://doi.org/10.1016/j.psychres.2020.112873)

Ozdemir, S., Lee, J. J., Yeo, K. K., Sim, K. L. D., Finkelstein, E. A., and Malhotra, C. (2023). A Prospective Cohort Study of Medical Decision-Making Roles and Their Associations with Patient Characteristics and Patient-Reported Outcomes among Patients with Heart Failure. *Med Decis Making* 43, 863–874. doi: [10.1177/0272989X231201609](https://doi.org/10.1177/0272989X231201609)

Ozdeniz Varan, E., and Gurvit, H. (2023). Effect of Dopaminergic Therapy on Impulse Control Disorders in Patients With a Prolactinoma. *Cognitive and Behavioral Neurology* 36, 1–8. doi: [10.1097/WNN.0000000000000320](https://doi.org/10.1097/WNN.0000000000000320)

Page, S. D., Lee, C., Aryal, S., Freedland, K., Stromberg, A., Vellone, E., et al. (2022). Development and testing of an instrument to measure contextual factors influencing self-care decisions among adults with chronic illness. *Health Qual Life Outcomes* 20, 83. doi: [10.1186/s12955-022-01990-2](https://doi.org/10.1186/s12955-022-01990-2)

Parr, A. C., Riek, H. C., Coe, B. C., Pari, G., Masellis, M., Marras, C., et al. (2023). Genetic variation in the dopamine system is associated with mixed‐strategy decision‐making in patients with Parkinson’s disease. *Eur J of Neuroscience* 58, 4523–4544. doi: [10.1111/ejn.15875](https://doi.org/10.1111/ejn.15875)

Passler, J. S., Kennedy, R. E., Clay, O. J., Crowe, M., Howard, V. J., Cushman, M., et al. (2020). The relationship of longitudinal cognitive change to self-reported IADL in a general population. *Aging, Neuropsychology, and Cognition*27, 125–139. doi: [10.1080/13825585.2019.1597008](https://doi.org/10.1080/13825585.2019.1597008)

Patai, E. Z., Foltynie, T., Limousin, P., Akram, H., Zrinzo, L., Bogacz, R., et al. (2022). Conflict Detection in a Sequential Decision Task Is Associated with Increased Cortico-Subthalamic Coherence and Prolonged Subthalamic Oscillatory Response in the β Band. *J. Neurosci.* 42, 4681–4692. doi: [10.1523/JNEUROSCI.0572-21.2022](https://doi.org/10.1523/JNEUROSCI.0572-21.2022)

Patt, V. M., Hunsberger, R., Jones, D. A., and Verfaellie, M. (2023). The Hippocampus Contributes to Temporal Discounting When Delays and Rewards Are Experienced in the Moment. *J. Neurosci.* 43, 5710–5722. doi: [10.1523/JNEUROSCI.2250-22.2023](https://doi.org/10.1523/JNEUROSCI.2250-22.2023)

Paul, R., Tsuei, T., Cho, K., Belden, A., Milanini, B., Bolzenius, J., et al. (2021). Ensemble machine learning classification of daily living abilities among older people with HIV. *EClinicalMedicine* 35, 100845. doi: [10.1016/j.eclinm.2021.100845](https://doi.org/10.1016/j.eclinm.2021.100845)

Perez, A. M., Spence, J. S., Kiel, L. D., Venza, E. E., and Chapman, S. B. (2018). Influential Cognitive Processes on Framing Biases in Aging. *Front. Psychol.* 9, 661. doi: [10.3389/fpsyg.2018.00661](https://doi.org/10.3389/fpsyg.2018.00661)

Pettorruso, M., Martinotti, G., Cocciolillo, F., De Risio, L., Cinquino, A., Di Nicola, M., et al. (2019). Striatal presynaptic dopaminergic dysfunction in gambling disorder: A ^123^ I‐FP‐CIT SPECT study. *Addiction Biology* 24, 1077–1086. doi: [10.1111/adb.12677](https://doi.org/10.1111/adb.12677)

Phan, T. N. T., Bertrand, P., Vo, X. V., and Jones, K. (2023). Investigating financial decision-making when facing skewed distributions of return: A survey study in Vietnam. *The Quarterly Review of Economics and Finance* 87, 318–329. doi: [10.1016/j.qref.2021.04.015](https://doi.org/10.1016/j.qref.2021.04.015)

Pikouli, F. A., Moraitou, D., Papantoniou, G., Sofologi, M., Papaliagkas, V., Kougioumtzis, G., et al. (2023). Metacognitive Strategy Training Improves Decision-Making Abilities in Amnestic Mild Cognitive Impairment. *J. Intell.* 11, 182. doi: [10.3390/jintelligence11090182](https://doi.org/10.3390/jintelligence11090182)

Prettyman, G. E., Kable, J. W., Didier, P., Shankar, S., Satterthwaite, T. D., Davatzikos, C., et al. (2021). Relationship of ventral striatum activation during effort discounting to clinical amotivation severity in schizophrenia. *npj Schizophr*7, 48. doi: [10.1038/s41537-021-00178-9](https://doi.org/10.1038/s41537-021-00178-9)

Pugliese, D., and Senna, H. (2018). Business Decision Making: Studying the Competence of Leaders. *GeP* 09, 01–19. doi: [10.5585/gep.v9i2.640](https://doi.org/10.5585/gep.v9i2.640)

Pushkarskaya, H., Tolin, D. F., Henick, D., Levy, I., and Pittenger, C. (2018). Unbending mind: Individuals with hoarding disorder do not modify decision strategy in response to feedback under risk. *Psychiatry Research* 259, 506–513. doi: [10.1016/j.psychres.2017.11.001](https://doi.org/10.1016/j.psychres.2017.11.001)

Quan, P., He, L., Mao, T., Fang, Z., Deng, Y., Pan, Y., et al. (2022). Cerebellum anatomy predicts individual risk-taking behavior and risk tolerance. *Neuroimage* 254, 119148. doi: [10.1016/j.neuroimage.2022.119148](https://doi.org/10.1016/j.neuroimage.2022.119148)

Quinn, C. G., Rabin, L. A., and Sprehn, G. C. (2018). The Association of Judgement Ability and Functional Status in Older Adult Rehabilitation Inpatients. *Brain Impairment* 19, 235–245. doi: [10.1017/BrImp.2018.8](https://doi.org/10.1017/BrImp.2018.8)

Rabin, L. A., Guayara-Quinn, C. G., Nester, C. O., Ellis, L., and Paré, N. (2022). Informant report of practical judgment ability in a clinical sample of older adults with subjective cognitive decline, mild cognitive impairment, and dementia. *Aging, Neuropsychology, and Cognition* 29, 139–157. doi: [10.1080/13825585.2020.1859081](https://doi.org/10.1080/13825585.2020.1859081)

Raina, A., Rajan, R., Sarma, G., Krishnan, S., Kesavapisharady, K., and Kishore, A. (2021). Learning from negative consequences is impaired by STN-DBS and levodopa in Parkinson’s disease. *Annals of Movement Disorders* 4, 66–72. doi: [10.4103/AOMD.AOMD_54_20](https://doi.org/10.4103/AOMD.AOMD_54_20)

Rajan, R., Krishnan, S., Sarma, G., Sarma, S. P., and Kishore, A. (2018). Dopamine Receptor D3 rs6280 is Associated with Aberrant Decision‐Making in Parkinson’s Disease. *Movement Disord Clin Pract* 5, 413–416. doi: [10.1002/mdc3.12631](https://doi.org/10.1002/mdc3.12631)

Ramchandran, K., Fiedorowicz, J., Chen, Z., Bu, Y., Bechara, A., and Andreasen, N. C. (2020a). Patients on the psychosis spectrum employ an alternate brain network to engage in complex decision-making. *PLoS ONE* 15, e0238774. doi: [10.1371/journal.pone.0238774](https://doi.org/10.1371/journal.pone.0238774)

Ramchandran, K., Tranel, D., Duster, K., and Denburg, N. L. (2020b). The Role of Emotional vs. Cognitive Intelligence in Economic Decision-Making Amongst Older Adults. *Front. Neurosci.* 14, 497. doi: [10.3389/fnins.2020.00497](https://doi.org/10.3389/fnins.2020.00497)

Reddy, L. F., Reavis, E. A., Wynn, J. K., and Green, M. F. (2018). Pupillary responses to a cognitive effort task in schizophrenia. *Schizophrenia Research* 199, 53–57. doi: [10.1016/j.schres.2018.03.005](https://doi.org/10.1016/j.schres.2018.03.005)

Renz, K. E., and Lincoln, T. M. (2022). The effect of salience of rewards on effort-based decision making in psychotic disorders. *BMC Psychiatry* 22, 643. doi: [10.1186/s12888-022-04274-7](https://doi.org/10.1186/s12888-022-04274-7)

Rinaldi, R., Lefebvre, L., Joachim, A., and Rossignol, M. (2020). Decision-making of patients with major depressive disorder in the framework of action control. *Cognitive Neuropsychiatry* 25, 71–83. doi: [10.1080/13546805.2019.1685481](https://doi.org/10.1080/13546805.2019.1685481)

Rodriguez, M., and Ellis, A. (2018). The Neuropsychological Function of Older First-Time Child Exploitation Material Offenders: A Pilot Study. *Int J Offender Ther Comp Criminol* 62, 2357–2373. doi: [10.1177/0306624X17703406](https://doi.org/10.1177/0306624X17703406)

Rogge, N. (2022). Exploring maximizing, satisficing and minimizing tendency in decision-making among autistic and neurotypical individuals. *Research in Autism Spectrum Disorders* 92, 101935. doi: [10.1016/j.rasd.2022.101935](https://doi.org/10.1016/j.rasd.2022.101935)

Rönneikkö, J., Huhtala, H., Finne-Soveri, H., Valvanne, J., and Jämsen, E. (2023). The role of geriatric syndromes in predicting unplanned hospitalizations: a population-based study using Minimum Data Set for Home Care. *BMC Geriatr* 23, 696. doi: [10.1186/s12877-023-04408-w](https://doi.org/10.1186/s12877-023-04408-w)

Rönnlund, M., Del Missier, F., Mäntylä, T., and Carelli, M. G. (2019). The Fatalistic Decision Maker: Time Perspective, Working Memory, and Older Adults’ Decision-Making Competence. *Front. Psychol.* 10, 2038. doi: [10.3389/fpsyg.2019.02038](https://doi.org/10.3389/fpsyg.2019.02038)

Rosario, C. O., Puts, M., Jang, R., Bezjak, A., Yokom, D., and Alibhai, S. M. H. (2019). Exploring the geriatric needs of oncology inpatients at an academic cancer centre. *Journal of Geriatric Oncology* 10, 824–828. doi: [10.1016/j.jgo.2018.12.001](https://doi.org/10.1016/j.jgo.2018.12.001)

Rosi, A., Bruine De Bruin, W., Del Missier, F., Cavallini, E., and Russo, R. (2019). Decision-making competence in younger and older adults: which cognitive abilities contribute to the application of decision rules? *Aging, Neuropsychology, and Cognition* 26, 174–189. doi: [10.1080/13825585.2017.1418283](https://doi.org/10.1080/13825585.2017.1418283)

Ruppin, S., Arias, O., and Dar, R. (2023). OCD symptoms are related to seeking and relying on external information even in neutral perceptual decisions. *Journal of Obsessive-Compulsive and Related Disorders* 39, 100843. doi: [10.1016/j.jocrd.2023.100843](https://doi.org/10.1016/j.jocrd.2023.100843)

Saleh, Y., Jarratt-Barnham, I., Petitet, P., Fernandez-Egea, E., Manohar, S. G., and Husain, M. (2023). Negative symptoms and cognitive impairment are associated with distinct motivational deficits in treatment resistant schizophrenia. *Mol Psychiatry* 28, 4831–4841. doi: [10.1038/s41380-023-02232-7](https://doi.org/10.1038/s41380-023-02232-7)

Saleh, Y., Le Heron, C., Petitet, P., Veldsman, M., Drew, D., Plant, O., et al. (2021). Apathy in small vessel cerebrovascular disease is associated with deficits in effort-based decision making. *Brain* 144, 1247–1262. doi: [10.1093/brain/awab013](https://doi.org/10.1093/brain/awab013)

Şandor, S., Yağcı Kurdish, S., Delil, Ş., Türk, B. G., and Yeni, S. N. (2022). The comparison of decision-making in ambiguous situations and galvanic skin responses as somatic markers in patients with posterior cortex epilepsy and mesial temporal lobe epilepsy with hippocampal sclerosis. *Journal of Clinical and Experimental Neuropsychology*44, 743–754. doi: [10.1080/13803395.2022.2164256](https://doi.org/10.1080/13803395.2022.2164256)

Santos, R. L., Simões Neto, J. P., Belfort, T., Lacerda, I. B., and Dourado, M. C. N. (2022). Patterns of impairment in decision-making capacity in Alzheimer’s disease and its relationship with cognitive and clinical variables. *Braz. J. Psychiatry* 44, 271–278. doi: [10.1590/1516-4446-2021-2180](https://doi.org/10.1590/1516-4446-2021-2180)

Santoyo-Mora, M., Villaseñor-Mora, C., Cardona-Torres, L. M., Martínez-Nolasco, J. J., Barranco-Gutiérrez, A. I., Padilla-Medina, J. A., et al. (2022). COVID-19 Long-Term Effects: Is There an Impact on the Simple Reaction Time and Alternative-Forced Choice on Recovered Patients? *Brain Sciences* 12, 1258. doi: [10.3390/brainsci12091258](https://doi.org/10.3390/brainsci12091258)

Saragih, I. D., Everard, G., and Lee, B.-O. (2022). A systematic review and meta-analysis of randomized controlled trials on the effect of serious games on people with dementia. *Ageing Research Reviews* 82, 101740. doi: [10.1016/j.arr.2022.101740](https://doi.org/10.1016/j.arr.2022.101740)

Sasaki, J., Matsubara, T., Chen, C., Fujii, Y., Fujita, Y., Nakamuta, M., et al. (2023). Prefrontal activity during the emotional go/no-go task and computational markers of risk-based decision-making predict future relapse in alcohol use disorder. *Front. Psychiatry* 13, 1048152. doi: [10.3389/fpsyt.2022.1048152](https://doi.org/10.3389/fpsyt.2022.1048152)

Schellenberg, B. J. I., and Bailis, D. S. (2018). When decisions are clouded by passion: A look at casino patrons. *Motivation Science* 4, 274–279. doi: [10.1037/mot0000086](https://doi.org/10.1037/mot0000086)

Schmicker, M., Menze, I., Koch, D., Rumpf, U., Müller, P., Pelzer, L., et al. (2019). Decision-Making Deficits in Elderly Can Be Alleviated by Attention Training. *JCM* 8, 1131. doi: [10.3390/jcm8081131](https://doi.org/10.3390/jcm8081131)

Schmitz, J. M., Suchting, R., Green, C. E., Webber, H. E., Vincent, J., Moeller, F. G., et al. (2021). The effects of combination levodopa-ropinirole on cognitive improvement and treatment outcome in individuals with cocaine use disorder: A bayesian mediation analysis. *Drug and Alcohol Dependence* 225, 108800. doi: [10.1016/j.drugalcdep.2021.108800](https://doi.org/10.1016/j.drugalcdep.2021.108800)

Segura-Serralta, M., Ciscar, S., Blasco, L., Oltra-Cucarella, J., Roncero, M., Espert, R., et al. (2020). Contribution of executive functions to eating behaviours in obesity and eating disorders. *Behav. Cogn. Psychother.* 48, 725–733. doi: [10.1017/S1352465820000260](https://doi.org/10.1017/S1352465820000260)

Sehrig, S., Odenwald, M., and Rockstroh, B. (2021). Feedback-Related Brain Potentials Indicate the Influence of Craving on Decision-Making in Patients with Alcohol Use Disorder: An Experimental Study. *Eur Addict Res* 27, 216–226. doi: [10.1159/000511417](https://doi.org/10.1159/000511417)

Serra, L., Scocchia, M., Meola, G., D’Amelio, M., Bruschini, M., Silvestri, G., et al. (2020). Ventral tegmental area dysfunction affects decision-making in patients with myotonic dystrophy type-1. *Cortex* 128, 192–202. doi: [10.1016/j.cortex.2020.03.022](https://doi.org/10.1016/j.cortex.2020.03.022)

Seubert-Ravelo, A. N., Yáñez-Téllez, M. G., Lazo-Barriga, M. L., Calderón Vallejo, A., Martínez-Cortés, C. E., and Hernández-Galván, A. (2021). Social Cognition in Patients with Early-Onset Parkinson’s Disease. *Parkinson’s Disease* 2021, 1–13. doi: [10.1155/2021/8852087](https://doi.org/10.1155/2021/8852087)

Sharma, R., Mallick, D., Llinas, R. H., and Marsh, E. B. (2020). Early Post-stroke Cognition: In-hospital Predictors and the Association With Functional Outcome. *Front. Neurol.* 11, 613607. doi: [10.3389/fneur.2020.613607](https://doi.org/10.3389/fneur.2020.613607)

Sharman, S., Clark, L., Roberts, A., Michalczuk, R., Cocks, R., and Bowden-Jones, H. (2019). Heterogeneity in Disordered Gambling: Decision-Making and Impulsivity in Gamblers Grouped by Preferred Form. *Front. Psychiatry*10, 588. doi: [10.3389/fpsyt.2019.00588](https://doi.org/10.3389/fpsyt.2019.00588)

Shaverdian, N., Kishan, A. U., Veruttipong, D., Demanes, D. J., Kupelian, P., McCloskey, S., et al. (2018). Impact of the Primary Information Source Used for Decision Making on Treatment Perceptions and Regret in Prostate Cancer. *American Journal of Clinical Oncology* 41, 898–904. doi: [10.1097/COC.0000000000000387](https://doi.org/10.1097/COC.0000000000000387)

Shepherd, V., Hood, K., Gillies, K., and Wood, F. (2022). Development of a measure to assess the quality of proxy decisions about research participation on behalf of adults lacking capacity to consent: the Combined Scale for Proxy Informed Consent Decisions (CONCORD scale). *Trials* 23, 843. doi: [10.1186/s13063-022-06787-8](https://doi.org/10.1186/s13063-022-06787-8)

Shi, S. M., McCarthy, E. P., Mitchell, S. L., and Kim, D. H. (2020). Predicting Mortality and Adverse Outcomes: Comparing the Frailty Index to General Prognostic Indices. *J GEN INTERN MED* 35, 1516–1522. doi: [10.1007/s11606-020-05700-w](https://doi.org/10.1007/s11606-020-05700-w)

Sinclair, C., Eramudugolla, R., Brady, B., Cherbuin, N., and Anstey, K. J. (2021). The role of cognition and reinforcement sensitivity in older adult decision-making under explicit risk conditions. *Journal of Clinical and Experimental Neuropsychology* 43, 238–254. doi: [10.1080/13803395.2021.1909709](https://doi.org/10.1080/13803395.2021.1909709)

Sinclair, C., Eramudugolla, R., Cherbuin, N., Mortby, M. E., and Anstey, K. J. (2023). The impact of mild cognitive impairment on decision-making under explicit risk conditions: Evidence from the Personality and Total Health (PATH) Through Life longitudinal study. *J Int Neuropsychol Soc* 29, 594–604. doi: [10.1017/S1355617722000765](https://doi.org/10.1017/S1355617722000765)

Slaughter, K. B., Meyer, E. G., Bambhroliya, A. B., Meeks, J. R., Ahmed, W., Bowry, R., et al. (2019). Direct Assessment of Health Utilities Using the Standard Gamble Among Patients With Primary Intracerebral Hemorrhage. *Circ: Cardiovascular Quality and Outcomes* 12, e005606. doi: [10.1161/CIRCOUTCOMES.119.005606](https://doi.org/10.1161/CIRCOUTCOMES.119.005606)

Smith, R., Kirlic, N., Stewart, J. L., Touthang, J., Kuplicki, R., Khalsa, S. S., et al. (2021). Greater decision uncertainty characterizes a transdiagnostic patient sample during approach-avoidance conflict: a computational modelling approach. *jpn* 46, E74–E87. doi: [10.1503/jpn.200032](https://doi.org/10.1503/jpn.200032)

Smith, R., Schwartenbeck, P., Stewart, J. L., Kuplicki, R., Ekhtiari, H., and Paulus, M. P. (2020). Imprecise action selection in substance use disorder: Evidence for active learning impairments when solving the explore-exploit dilemma. *Drug and Alcohol Dependence* 215, 108208. doi: [10.1016/j.drugalcdep.2020.108208](https://doi.org/10.1016/j.drugalcdep.2020.108208)

Smith, V., Pinasco, C., Achterberg, J., Mitchell, D. J., Das, T., Roca, M., et al. (2022). Fluid intelligence and naturalistic task impairments after focal brain lesions. *Cortex* 146, 106–115. doi: [10.1016/j.cortex.2021.09.020](https://doi.org/10.1016/j.cortex.2021.09.020)

Sobkow, A., Olszewska, A., and Traczyk, J. (2020). Multiple numeric competencies predict decision outcomes beyond fluid intelligence and cognitive reflection. *Intelligence* 80, 101452. doi: [10.1016/j.intell.2020.101452](https://doi.org/10.1016/j.intell.2020.101452)

Song, M.-K., Ward, S. E., Hepburn, K., Paul, S., Kim, H., Shah, R. C., et al. (2019). Can Persons with Dementia Meaningfully Participate in Advance Care Planning Discussions? A Mixed-Methods Study of SPIRIT. *Journal of Palliative Medicine* 22, 1410–1416. doi: [10.1089/jpm.2019.0088](https://doi.org/10.1089/jpm.2019.0088)

Spataro, R., and La Bella, V. (2021). The capacity to consent to treatment in amyotrophic lateral sclerosis: a preliminary report. *J Neurol* 268, 219–226. doi: [10.1007/s00415-020-10136-7](https://doi.org/10.1007/s00415-020-10136-7)

Spohn, D. L., Devore‐Suazo, I. F., Bernarding, M. L., and Güss, C. D. (2022). The role of social context in risky decision‐making: Presence of friend and low resistance to peer influence increase risky decision‐making. *Int J Psychol* 57, 717–726. doi: [10.1002/ijop.12864](https://doi.org/10.1002/ijop.12864)

Steffen, J., Marković, D., Glöckner, F., Neukam, P. T., Kiebel, S. J., Li, S.-C., et al. (2023). Shorter planning depth and higher response noise during sequential decision-making in old age. *Sci Rep* 13, 7692. doi: [10.1038/s41598-023-33274-0](https://doi.org/10.1038/s41598-023-33274-0)

Steward, K. A., Bull, T. P., Kennedy, R., Crowe, M., and Wadley, V. G. (2019a). Neuropsychological Correlates of Anosognosia for Objective Functional Difficulties in Older Adults on the Mild Cognitive Impairment Spectrum. *Archives of Clinical Neuropsychology* 35, 365–376. doi: [10.1093/arclin/acz065](https://doi.org/10.1093/arclin/acz065)

Steward, K. A., Bull, T. P., and Wadley, V. G. (2019b). Differences in self-awareness of functional deficits between amnestic single- and multidomain mild cognitive impairment. *Journal of Clinical and Experimental Neuropsychology* 41, 544–553. doi: [10.1080/13803395.2019.1586839](https://doi.org/10.1080/13803395.2019.1586839)

Steward, K. A., Kennedy, R., Erus, G., Nasrallah, I. M., and Wadley, V. G. (2019c). Poor awareness of IADL deficits is associated with reduced regional brain volume in older adults with cognitive impairment. *Neuropsychologia* 129, 372–378. doi: [10.1016/j.neuropsychologia.2019.04.023](https://doi.org/10.1016/j.neuropsychologia.2019.04.023)

Stewart, C. C., Yu, L., Wilson, R. S., Bennett, D. A., and Boyle, P. A. (2018). Correlates of healthcare and financial decision making among older adults without dementia. *Health Psychology* 37, 618–626. doi: [10.1037/hea0000610](https://doi.org/10.1037/hea0000610)

Stolz, D. S., Vater, A., Schott, B. H., Roepke, S., Paulus, F. M., and Krach, S. (2021). Reduced frontal cortical tracking of conflict between self-beneficial versus prosocial motives in Narcissistic Personality Disorder. *NeuroImage: Clinical*32, 102800. doi: [10.1016/j.nicl.2021.102800](https://doi.org/10.1016/j.nicl.2021.102800)

Strikwerda-Brown, C., Ramanan, S., Goldberg, Z.-L., Mothakunnel, A., Hodges, J. R., Ahmed, R. M., et al. (2021). The interplay of emotional and social conceptual processes during moral reasoning in frontotemporal dementia. *Brain*144, 938–952. doi: [10.1093/brain/awaa435](https://doi.org/10.1093/brain/awaa435)

Suetani, S., Baker, A., Garner, K., Cosgrove, P., Mackay-Sim, M., Siskind, D., et al. (2022). Impairments in goal-directed action and reversal learning in a proportion of individuals with psychosis. *Cogn Affect Behav Neurosci* 22, 1390–1403. doi: [10.3758/s13415-022-01026-8](https://doi.org/10.3758/s13415-022-01026-8)

Sugden, R., Wang, M., and Zizzo, D. J. (2019). Take it or leave it: Experimental evidence on the effect of time-limited offers on consumer behaviour. *Journal of Economic Behavior & Organization* 168, 1–23. doi: [10.1016/j.jebo.2019.09.008](https://doi.org/10.1016/j.jebo.2019.09.008)

Sullivan, K. A., Purser, K., Graham, K., and Parkinson, L. (2023). Public awareness of legal decision-making capacity and planning instruments in dementia: implications for health care practitioners. *Psychiatry, Psychology and Law* 30, 565–578. doi: [10.1080/13218719.2022.2060364](https://doi.org/10.1080/13218719.2022.2060364)

Sulu, C., Gul, N., Tanrikulu, S., Ciftci, S., Yener Ozturk, F., Sarac, B., et al. (2023). Risk of impulse control disorders in patients with Cushing’s disease: do not blame cabergoline but do not give up caution. *Pituitary* 26, 495–509. doi: [10.1007/s11102-023-01342-3](https://doi.org/10.1007/s11102-023-01342-3)

Sun, T., Xie, T., Wang, J., Zhang, L., Tian, Y., Wang, K., et al. (2020). Decision-Making Under Ambiguity or Risk in Individuals With Alzheimer’s Disease and Mild Cognitive Impairment. *Front. Psychiatry* 11, 218. doi: [10.3389/fpsyt.2020.00218](https://doi.org/10.3389/fpsyt.2020.00218)

Szanto, K., Galfalvy, H., Vanyukov, P. M., Keilp, J. G., and Dombrovski, A. Y. (2018). Pathways to Late-Life Suicidal Behavior: Cluster Analysis and Predictive Validation of Suicidal Behavior in a Sample of Older Adults With Major Depression. *J. Clin. Psychiatry* 79, 17m11611. doi: [10.4088/JCP.17m11611](https://doi.org/10.4088/JCP.17m11611)

Tabira, T., Hotta, M., Maruta, M., Ikeda, Y., Shimokihara, S., Han, G., et al. (2022). Characteristic of process analysis on instrumental activities of daily living according to the severity of cognitive impairment in community-dwelling older adults with Alzheimer’s disease. *International Psychogeriatrics* 36, 188–199. doi: [10.1017/S1041610222000552](https://doi.org/10.1017/S1041610222000552)

Tannou, T., Magnin, E., Comte, A., Aubry, R., and Joubert, S. (2021). Neural Activation in Risky Decision-Making Tasks in Healthy Older Adults: A Meta-Analysis of fMRI Data. *Brain Sciences* 11, 1043. doi: [10.3390/brainsci11081043](https://doi.org/10.3390/brainsci11081043)

Tarantino, V., Tasca, I., Giannetto, N., Mangano, G. R., Turriziani, P., and Oliveri, M. (2021). Impact of Perceived Stress and Immune Status on Decision-Making Abilities during COVID-19 Pandemic Lockdown. *Behavioral Sciences* 11, 167. doi: [10.3390/bs11120167](https://doi.org/10.3390/bs11120167)

Testa, G., Mora-Maltas, B., Camacho-Barcia, L., Granero, R., Lucas, I., Agüera, Z., et al. (2021). Transdiagnostic Perspective of Impulsivity and Compulsivity in Obesity: From Cognitive Profile to Self-Reported Dimensions in Clinical Samples with and without Diabetes. *Nutrients* 13, 4426. doi: [10.3390/nu13124426](https://doi.org/10.3390/nu13124426)

Thiebaut, S., Jaussent, I., Maimoun, L., Beziat, S., Seneque, M., Hamroun, D., et al. (2019). Impact of bipolar disorder on eating disorders severity in real-life settings. *Journal of Affective Disorders* 246, 867–872. doi: [10.1016/j.jad.2018.12.128](https://doi.org/10.1016/j.jad.2018.12.128)

Thrailkill, E. A., DeSarno, M., and Higgins, S. T. (2022). Loss aversion and risk for cigarette smoking and other substance use. *Drug and Alcohol Dependence* 232, 109307. doi: [10.1016/j.drugalcdep.2022.109307](https://doi.org/10.1016/j.drugalcdep.2022.109307)

Tikàsz, A., Dumais, A., Lipp, O., Stip, E., Lalonde, P., Laurelli, M., et al. (2019). Reward-related decision-making in schizophrenia: A multimodal neuroimaging study. *Psychiatry Research: Neuroimaging* 286, 45–52. doi: [10.1016/j.pscychresns.2019.03.007](https://doi.org/10.1016/j.pscychresns.2019.03.007)

Toi, A. K., Ben Charif, A., Lai, C., Ngueta, G., Plourde, K. V., Stacey, D., et al. (2022). Difficult Decisions for Older Canadians Receiving Home Care, and Why They Are So Difficult: A Web-Based Decisional Needs Assessment. *MDM Policy & Practice* 7, 23814683221124090. doi: [10.1177/23814683221124090](https://doi.org/10.1177/23814683221124090)

Tolbert, S., Liu, Y., Hellegers, C., Petrella, J. R., Weiner, M. W., Wong, T. Z., et al. (2019). Financial Management Skills in Aging, MCI and Dementia: Cross Sectional Relationship to 18F-Florbetapir PET Cortical β-amyloid Deposition. *The Journal of Prevention of Alzheimer’s Disease* 6, 274–282. doi: [10.14283/jpad.2019.26](https://doi.org/10.14283/jpad.2019.26)

Torroella Carney, M., Emmert, B., and Keefe, B. (2018). The Bedside Capacity Assessment Tool: Further Development of a Clinical Tool to Assist with a Growing Aging Population with Increased Healthcare Complexities. *The Journal of Clinical Ethics* 29, 43–51. doi: [10.1086/JCE2018291043](https://doi.org/10.1086/JCE2018291043)

Tsuda, S., Nakamura, M., Miyachi, J., Matsui, Y., Takagi, M., Ohashi, H., et al. (2019). Decisional Conflict in Home Medical Care in a Family-Oriented Society: Family Members’ Perspectives on Surrogate Decision Making from a Multicenter Cohort Study. *Journal of Palliative Medicine* 22, 814–822. doi: [10.1089/jpm.2018.0493](https://doi.org/10.1089/jpm.2018.0493)

Urbietė, L., Lesauskaitė, V., and Macijauskienė, J. (2020). Discharge Planning and Home Care Needs Assessment for Older Patients in a Nursing Hospital. *Medicina* 56, 60. doi: [10.3390/medicina56020060](https://doi.org/10.3390/medicina56020060)

Van Den Berg, N. S., Huitema, R. B., Spikman, J. M., Luijckx, G.-J., and De Haan, E. H. F. (2020a). Impairments in Emotion Recognition and Risk-Taking Behavior After Isolated, Cerebellar Stroke. *Cerebellum* 19, 419–425. doi: [10.1007/s12311-020-01121-x](https://doi.org/10.1007/s12311-020-01121-x)

Van Den Berg, N. S., Reesink, F. E., De Haan, E. H. F., Kremer, H. P. H., Spikman, J. M., and Huitema, R. B. (2020b). Emotion Recognition and Traffic-Related Risk-Taking Behavior in Patients with Neurodegenerative Diseases. *J Int Neuropsychol Soc* 27, 136–145. doi: [10.1017/S1355617720000740](https://doi.org/10.1017/S1355617720000740)

van den Berk-Clark, C., Myerson, J., Green, L., and Grucza, R. A. (2018). Past trauma and future choices: differences in discounting in low-income, urban African Americans. *Psychol Med* 48, 2702–2709. doi: [10.1017/S0033291718000326](https://doi.org/10.1017/S0033291718000326)

Vella, L., Ring, H. A., Aitken, M. R., Watson, P. C., Presland, A., and Clare, I. C. (2018). Understanding self-reported difficulties in decision-making by people with autism spectrum disorders. *Autism* 22, 549–559. doi: [10.1177/1362361316687988](https://doi.org/10.1177/1362361316687988)

Verrijp, M., Dubbelman, M. A., Visser, L. N. C., Jutten, R. J., Nijhuis, E. W., Zwan, M. D., et al. (2022). Everyday Functioning in a Community-Based Volunteer Population: Differences Between Participant- and Study Partner-Report. *Front. Aging Neurosci.* 13, 761932. doi: [10.3389/fnagi.2021.761932](https://doi.org/10.3389/fnagi.2021.761932)

Verveer, I., Van Der Veen, F. M., Shahbabaie, A., Remmerswaal, D., and Franken, I. H. A. (2020). Multi-session electrical neuromodulation effects on craving, relapse and cognitive functions in cocaine use disorder: A randomized, sham-controlled tDCS study. *Drug and Alcohol Dependence* 217, 108429. doi: [10.1016/j.drugalcdep.2020.108429](https://doi.org/10.1016/j.drugalcdep.2020.108429)

Vilà-Balló, A., De La Cruz-Puebla, M., López-Barroso, D., Miró, J., Sala-Padró, J., Cucurell, D., et al. (2022). Reward-based decision-making in mesial temporal lobe epilepsy patients with unilateral hippocampal sclerosis pre- and post-surgery. *NeuroImage: Clinical* 36, 103251. doi: [10.1016/j.nicl.2022.103251](https://doi.org/10.1016/j.nicl.2022.103251)

Wadley, V. G., Bull, T. P., Zhang, Y., Barba, C., Bryan, R. N., Crowe, M., et al. (2020). Cognitive Processing Speed Is Strongly Related to Driving Skills, Financial Abilities, and Other Instrumental Activities of Daily Living in Persons With Mild Cognitive Impairment and Mild Dementia. *The Journals of Gerontology: Series A* 76, 1829–1838. doi: [10.1093/gerona/glaa312](https://doi.org/10.1093/gerona/glaa312)

Wagenbreth, C., Kuehne, M., Voges, J., Heinze, H.-J., Galazky, I., and Zaehle, T. (2019). Deep Brain Stimulation of the Subthalamic Nucleus Selectively Modulates Emotion Recognition of Facial Stimuli in Parkinson’s Patients. *JCM* 8, 1335. doi: [10.3390/jcm8091335](https://doi.org/10.3390/jcm8091335)

Walker, K. A., and Brown, G. G. (2018). HIV-associated executive dysfunction in the era of modern antiretroviral therapy: A systematic review and meta-analysis. *Journal of Clinical and Experimental Neuropsychology* 40, 357–376. doi: [10.1080/13803395.2017.1349879](https://doi.org/10.1080/13803395.2017.1349879)

Wang, W., Zhu, Y., Wang, L., Mu, L., Zhu, L., Ding, D., et al. (2022). High-frequency repetitive transcranial magnetic stimulation of the left dorsolateral prefrontal cortex reduces drug craving and improves decision-making ability in methamphetamine use disorder. *Psychiatry Research* 317, 114904. doi: [10.1016/j.psychres.2022.114904](https://doi.org/10.1016/j.psychres.2022.114904)

Wang, Y., Wang, X., Wang, K., Zhao, B., and Chen, X. (2021). Decision‐making impairments under ambiguous and risky situations in patients with prefrontal tumor: A neuropsychological study. *Brain and Behavior* 11, e01951. doi: [10.1002/brb3.1951](https://doi.org/10.1002/brb3.1951)

Wei, F.-C., Hsu, C.-K., Wu, Y.-L., Liao, J.-Y., Huang, C.-H., Hsiung, C. A., et al. (2022). Reliability and Validity of the Traditional Chinese Version of the Advance Care Planning Engagement Survey: A Pilot Evaluation in Taiwanese Outpatients. *J Palliat Care* 37, 273–279. doi: [10.1177/08258597211051208](https://doi.org/10.1177/08258597211051208)

Weller, J. A., King, M. L., Figner, B., and Denburg, N. L. (2019). Information use in risky decision making: Do age differences depend on affective context? *Psychology and Aging* 34, 1005–1020. doi: [10.1037/pag0000397](https://doi.org/10.1037/pag0000397)

Weygandt, M., Behrens, J., Brasanac, J., Söder, E., Meyer-Arndt, L., Wakonig, K., et al. (2019). Neural mechanisms of perceptual decision-making and their link to neuropsychiatric symptoms in multiple sclerosis. *Multiple Sclerosis and Related Disorders* 33, 139–145. doi: [10.1016/j.msard.2019.05.025](https://doi.org/10.1016/j.msard.2019.05.025)

Weygandt, M., Wakonig, K., Behrens, J., Meyer-Arndt, L., Söder, E., Brandt, A. U., et al. (2018). Brain activity, regional gray matter loss, and decision-making in multiple sclerosis. *Mult Scler* 24, 1163–1173. doi: [10.1177/1352458517717089](https://doi.org/10.1177/1352458517717089)

Wheaton, M. G., and Topilow, K. (2020). Maximizing decision-making style and hoarding disorder symptoms. *Comprehensive Psychiatry* 101, 152187. doi: [10.1016/j.comppsych.2020.152187](https://doi.org/10.1016/j.comppsych.2020.152187)

Wild, K., Marcoe, J., Mattek, N., Sharma, N., Loewy, E., Tischler, H., et al. (2022). Online monitoring of financial capacity in older adults: Feasibility and initial findings. *Alz &amp; Dem Diag Ass &amp; Dis Mo* 14, e12282. doi: [10.1002/dad2.12282](https://doi.org/10.1002/dad2.12282)

Wilson, L., Zheng, P., Ionova, Y., Denham, A., Yoo, C., Ma, Y., et al. (2023). CAPER: patient preferences to inform nonsurgical treatment of chronic low back pain: a discrete-choice experiment. *Pain Medicine* 24, 963–973. doi: [10.1093/pm/pnad038](https://doi.org/10.1093/pm/pnad038)

Wilson, M. J., and Vassileva, J. (2018). Decision-Making Under Risk, but Not Under Ambiguity, Predicts Pathological Gambling in Discrete Types of Abstinent Substance Users. *Front. Psychiatry* 9, 239. doi: [10.3389/fpsyt.2018.00239](https://doi.org/10.3389/fpsyt.2018.00239)

Wölfling, K., Duven, E., Wejbera, M., Beutel, M. E., and Müller, K. W. (2020). Discounting delayed monetary rewards and decision making in behavioral addictions – A comparison between patients with gambling disorder and internet gaming disorder. *Addictive Behaviors* 108, 106446. doi: [10.1016/j.addbeh.2020.106446](https://doi.org/10.1016/j.addbeh.2020.106446)

Wong, P. K. S., and Chow, A. Y. M. (2021). Self-Determination Competencies, (Dis)Agreement in Decision-Making, and Personal Well-Being of Adults with Mild Intellectual Disabilities in Hong Kong. *IJERPH* 18, 10721. doi: [10.3390/ijerph182010721](https://doi.org/10.3390/ijerph182010721)

Wu, H.-C., White, S., Rees, G., and Burgess, P. W. (2018). Executive function in high-functioning autism: Decision-making consistency as a characteristic gambling behaviour. *Cortex* 107, 21–36. doi: [10.1016/j.cortex.2018.01.013](https://doi.org/10.1016/j.cortex.2018.01.013)

Wyld, L., Reed, M. W. R., Collins, K., Burton, M., Lifford, K., Edwards, A., et al. (2021). Bridging the age gap in breast cancer: cluster randomized trial of two decision support interventions for older women with operable breast cancer on quality of life, survival, decision quality, and treatment choices. *British Journal of Surgery* 108, 499–510. doi: [10.1093/bjs/znab005](https://doi.org/10.1093/bjs/znab005)

Xu, M., Lee, W.-K., Ko, C.-H., Chiu, Y.-C., and Lin, C.-H. (2021). The Prominent Deck B Phenomenon in Schizophrenia: An Empirical Study on Iowa Gambling Task. *Front. Psychol.* 12, 619855. doi: [10.3389/fpsyg.2021.619855](https://doi.org/10.3389/fpsyg.2021.619855)

Yamashita, M., Kamiya, K., Hamazaki, N., Matsuzawa, R., Nozaki, K., Ichikawa, T., et al. (2020). Prognostic value of instrumental activity of daily living in initial heart failure hospitalization patients aged 65 years or older. *Heart Vessels* 35, 360–366. doi: [10.1007/s00380-019-01490-2](https://doi.org/10.1007/s00380-019-01490-2)

Yang, Z., Hou, B., Chen, P., and Zhang, H. (2021). Preference and Influencing Factors of Advance Care Planning for Chinese Elderly Patients With Chronic Diseases: A Mixed-Methods Approach. *J Hosp Palliat Nurs* 23, 178–186. doi: [10.1097/NJH.0000000000000734](https://doi.org/10.1097/NJH.0000000000000734)

Yazdi, K., Rumetshofer, T., Gnauer, M., Csillag, D., Rosenleitner, J., and Kleiser, R. (2019). Neurobiological processes during the Cambridge gambling task. *Behavioural Brain Research* 356, 295–304. doi: [10.1016/j.bbr.2018.08.017](https://doi.org/10.1016/j.bbr.2018.08.017)

Ye, G., Qu, B., Shi, W., Chen, X., Ma, P., Zhong, Y., et al. (2020). Knowledge about benefits and risks of undergoing cataract surgery among cataract patients in Southern China. *Int Ophthalmol* 40, 2889–2899. doi: [10.1007/s10792-020-01473-7](https://doi.org/10.1007/s10792-020-01473-7)

Yeo, P. S., Nguyen, T. N., Ng, M. P. E., Choo, R. W. M., Yap, P. L. K., Ng, T. P., et al. (2021). Evaluation of the Implementation and Effectiveness of Community-Based Brain-Computer Interface Cognitive Group Training in Healthy Community-Dwelling Older Adults: Randomized Controlled Implementation Trial. *JMIR Form Res* 5, e25462. doi: [10.2196/25462](https://doi.org/10.2196/25462)

Yildirim, E., Altinayar, S., and Cakmur, R. (2020). Decision-Making and Impulse-Control Disorders in Parkinson’s Disease: Influence of Dopaminergic Treatment. *Neurological Sciences and Neurophysiology* 37, 11–17. doi: [10.4103/NSN.NSN_12_20](https://doi.org/10.4103/NSN.NSN_12_20)

Yuan, B., Li, J., and Lan, J. (2022). Labor Participation of Retirement-Aged Workers: Understanding the Influencing Mechanism of Health Status and Social Pension Insurance Participation. *Journal of Occupational & Environmental Medicine* 64, e60–e69. doi: [10.1097/JOM.0000000000002444](https://doi.org/10.1097/JOM.0000000000002444)

Yusupova, H., Abdullaeva, G., Khamidullaeva, G., and Alikhodzhaeva, F. (2023). Effect of Nitrendipine and Amlodipine on Cognitive Functions of Patients with Arterial Hypertension. *IJBM* 13, 217–223. doi: [10.21103/Article13(2)_OA2](https://doi.org/10.21103/Article13(2)_OA2)

Zamarian, L., Berger, T., Pertl, M., Bsteh, G., Djamshidian, A., Deisenhammer, F., et al. (2020). Decision making and framing effects in multiple sclerosis. *Euro J of Neurology* 28, 1292–1298. doi: [10.1111/ene.14669](https://doi.org/10.1111/ene.14669)

Zhang, L., Vashisht, H., Nethra, A., Slattery, B., and Ward, T. (2022a). Differences in Learning and Persistency Characterizing Behavior in Chronic Pain for the Iowa Gambling Task: Web-Based Laboratory-in-the-Field Study. *J Med Internet Res* 24, e26307. doi: [10.2196/26307](https://doi.org/10.2196/26307)

Zhang, Y., Wang, J., Sun, T., Wang, L., Li, T., Li, H., et al. (2022b). Decision-Making Profiles and Their Associations with Cognitive Performance in Mild Cognitive Impairment. *JAD* 87, 1215–1227. doi: [10.3233/JAD-215440](https://doi.org/10.3233/JAD-215440)

Zhao, I. Y., Ho, M.-H., Tyrovolas, S., Deng, S. Y., Montayre, J., and Molassiotis, A. (2023a). Constructing the concept of healthy ageing and examining its association with loneliness in older adults. *BMC Geriatr* 23, 325. doi: [10.1186/s12877-023-04019-5](https://doi.org/10.1186/s12877-023-04019-5)

Zhao, I. Y., Leung, A. Y. M., Deng, S. Y., Ho, M., Saravanakumar, P., Montayre, J., et al. (2023b). Intergenerational reciprocity and WHO function ability domains predict loneliness in older Chinese adults. *Australas J Ageing* 43, 112–122. doi: [10.1111/ajag.13250](https://doi.org/10.1111/ajag.13250)

Zhao, L., Zhao, Y., Su, D., Lv, Z., Xie, F., Hu, P., et al. (2023c). Cognitive Functions in Patients with Moderate-to-Severe Obstructive Sleep Apnea Syndrome with Emphasis on Executive Functions and Decision-Making. *Brain Sciences*13, 1436. doi: [10.3390/brainsci13101436](https://doi.org/10.3390/brainsci13101436)

Zhu, J., Xiang, Y.-B., Cai, H., Li, H., Gao, Y.-T., Zheng, W., et al. (2018). A Prospective Investigation of Dietary Intake and Functional Impairments Among the Elderly. *American Journal of Epidemiology*. doi: [10.1093/aje/kwy156](https://doi.org/10.1093/aje/kwy156)

Zilbershlag, Y., and Josman, N. (2019). The functional cognitive evaluation and model to assess older adults’ ability to function in their homes in the community. *Physical & Occupational Therapy In Geriatrics* 37, 151–170. doi: [10.1080/02703181.2019.1622620](https://doi.org/10.1080/02703181.2019.1622620)
